# Distribution of living coccolithophores in eastern Indian Ocean during spring intermonsoon

**DOI:** 10.1038/s41598-018-29688-w

**Published:** 2018-08-21

**Authors:** Haijiao Liu, Jun Sun, Dongxiao Wang, Xiaodong Zhang, Cuixia Zhang, Shuqun Song, Satheeswaran Thangaraj

**Affiliations:** 1 Institute of Marine Science and Technology, Shandong University, No. 27 Shanda Nan Road, Jinan, 250110 P.R. China; 2 Tianjin Key Laboratory of Marine Resources and Chemistry, Tianjin University of Science and Technology, No. 29 13th Avenue, Tianjin Economic-Technological Development Area, Tianjin, 300457 P.R. China; 3 College of Marine and Environmental Sciences, Tianjin University of Science and Technology, No. 29 13th Avenue, Tianjin Economic-Technological Development Area, Tianjin, 300457 P.R. China; 4 State Key Laboratory of Tropical Oceanography (LTO), South China Sea Institute of Oceanology, Chinese Academy of Sciences, Guangzhou, Guangdong 510301 P.R. China; 5 CAS Key Laboratory of Marine Ecology and Environmental Sciences, Institute of Oceanology, Chinese Academy of Sciences, No. 7 Nanhai Road, Qingdao, 266071 P.R. China

## Abstract

We studied the biodiversity of autotrophic calcareous coccolithophore assemblages at 30 locations in the Eastern Equatorial Indian Ocean (EEIO) (80°–94°E, 6°N–5°S) and evaluated the importance of regional hydrology. We documented 26 species based on the identification of coccospheres and coccoliths, respectively. The coccolithophore community was dominated by *Gephyrocapsa oceanica*, *Emiliania huxleyi*, *Florisphaera profunda*, *Umbilicosphaera sibogae*, and *Helicosphaera carteri*. The abundance of coccoliths and coccospheres ranged from 0.2 × 10^3^ to 160 × 10^3^ coccoliths l^−1^ and 0.2 × 10^3^ to 68 × 10^3^ cells l^−1^, averaged 23 × 10^3^ coccoliths l^−1^ and 9.4 × 10^3^ cells l^−1^, respectively. Biogenic PIC, POC, and rain ratio mean values were 0.50 μgC l^−1^, 1.047 μgC l^−1^, and 0.10 respectively. High abundances of both coccoliths and coccospheres in the surface ocean layer occurred on the north of the equator. Vertically, the great majority of coccoliths and coccospheres were concentrated in water taken from depths of <75 m. The ratios between the number of coccospheres and free coccoliths indicated that coccoliths experience different levels of dissolution when transported to deep water. Abundant coccolithophores mainly occurred at the west of 90°E, which is in accordance with the presence of Wyrtki jets. Patterns of coccolithosphores and of coccoliths have been reflected in hydrological processes.

## Introduction

Coccolithophores are thrived in the photic water column. They are the unicellular microalgal flagellates with diverse life cycles that (alternating diploid - haploid stage) belongs to marine nanoplankton^1,2^. Life phase transitions can easily occur in natural assemblages when nutrient level changes^3^. The coccolithophore cell is surrounded by one to several layers of coccoliths. Coccolithophores are globally distributed and contribute up to 10% of the global phytoplankton biomass^4–9^. In its dual functions of biomineralization and photoautotrophy, the coccolithophore community influences the global carbon cycle, sulphur cycle and other oceanographic parameters^3,10^. Inorganic calcareous coccoliths can serve as a ballast for organic carbon sequestration in the deep ocean^11–13^. As a consequence, the PIC/POC (particulate inorganic carbon to particulate organic carbon = “rain ratio”), is a factor modulating the biomineralization on the export of organic production. Coccolithophore assemblages are sensitive to climate variability^14,15^. The increased concentration of CO_2_ used to combined with other factors (e.g., nutrient elements, pH, irradiance, temperature) and stimulate the fixation of cell organic carbon by photosynthesis, thus the effect diminishing the rain ratio of coccolithophores^16–19^. These calcifying nanoplankton are negatively affected by ocean acidification with decreased availability of carbonate, especially in colder water realms^20,21^. The response of coccolithophore ecophysiology to environmental change has aroused much concern^22^. When detached coccoliths are exported to the deep sediment, they provide an ideal tool to record paleoenvironmental change, e.g. sea-surface temperatures, mixed layers and nutriclines^6,23–25^. Coccolithophore geographical distributions interact with physicochemical characteristics, thus making them useful in paleoenvironmental sediment records^−^ ^26^. Coccolithophore community structure and ecological distributions in the Atlantic Ocean have been documented by Brown and Yoder^5^, Baumann *et al*.^27^, Kinkel *et al*.^28^, and Shutler *et al*.^29^. Pacific Ocean studies have included by 9McIntyre *et al*.^30^, Okada and Honjo^31,32^, Okada and McIntyre^33^, Houghton and Guptha^34^, Saavedra-Pellitero *et al*.^35,36^, and López-Fuerte *et al*.^37^.

The Indian Ocean is the world’s third largest ocean basin, and it is strongly influenced by the South Asian monsoon system. The warm seawater area in the eastern equatorial Indian Ocean (EEIO) is a large region that influences worldwide climatology and El Niño/Southern Oscillation (ENSO) events^38,39^. The Indian Ocean dipole is another oceanic phenomenon influencing global oceanographic circulation^40^. Surface currents in the EEIO are seasonally dynamic due to the monsoon forces. Unlike most other ocean basins, the Indian Ocean experiences semiannual reversal of prevailing currents^41,42^. Many prevailing currents, however, persist in the EEIO during the summer and winter monsoon periods. These include the Equatorial undercurrent and the South Java Current^39,43^. Ocean currents also can exist throughout the year. One example is the Indonesian Through Flow (ITF), which is the passageway connecting the Pacific Ocean and Indian Ocean^44^. In the spring and fall inter monsoon periods, many surface circulations disappear, and Wyrtki jets (WJs) are the only semi-annual currents present at the equator. The equatorial Indian Ocean is controlled by the eastward WJs (also known as Equatorial Jets)^45^. Recently the studies on coccolithophores in the Indian Ocean have been relatively compared in Atlantic and Pacific Ocean studies. In the Indian Ocean the studies of coccolihophore have been made by Young^46^, Giraudeau and Bailey^47^, Broerse *et al*.^48^, Lees^49^, Andruleit^50^, Mohan *et al*.^51^, Mergulhao *et al*.^52^, about the nanofossil or living species biogeography in the monsoon season. Relatively few studies have evaluated the occurrence of living coccolithophores in the water column during the intermonsoon period in the eastern Indian Ocean. Our three main objectives were to (1) document the abundance, diversity and geographical patterns of living coccolithophores; (2) explain the variations occurring in nano flora assemblages; (3) correlate these variations to regional hydrographic parameters.

## Results

### Hydrographic features

**Figure 1:**
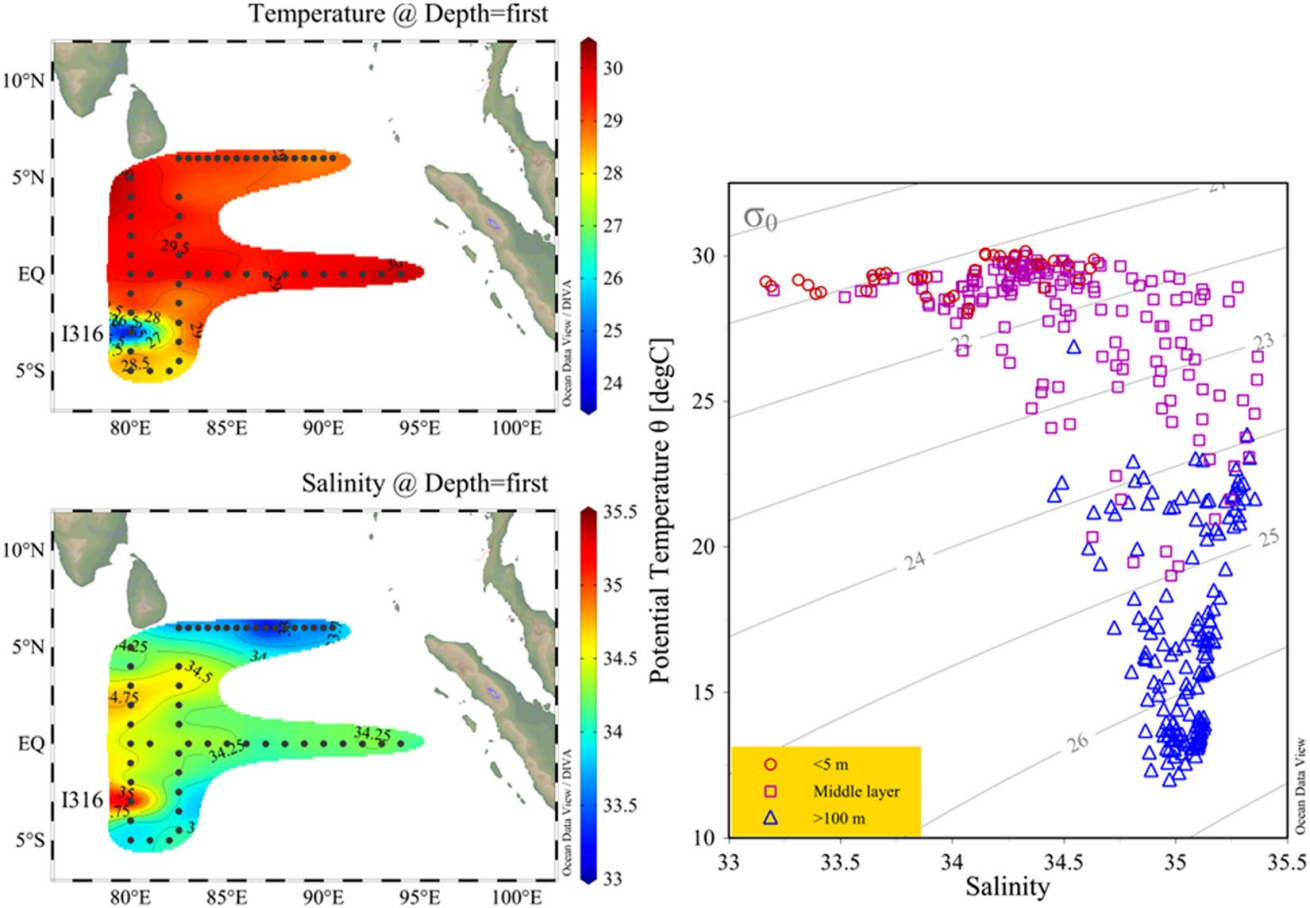
Sea surface temperature (°C) and salinity of the euphotic layer (0~200 m) in the surveyed area (left); Temperature-salinity (T-S) diagram in the surveyed area, three water regimes were characterized (right). Plotted using Ocean Data View (ODV) 4.7.6 software (https://odv.awi.de/en/).

The present investigation area is crossed by diverse hydrographic gradients as seen from the vertical profiles of temperature and salinity (data not shown). The temperature increased southwards along longitudinal section (Fig. 1a). Notably, there was an interesting phenomenon at St. I306 with the lowest temperature and highest salinity. High temperature and highly saline waters from the west equatorial zone were advected into the east equatorial zone (Fig. 1a,b). The temperature-salinity (T-S) curve can be divided into three regions: high temperature & low salinity surface water, intermediate temperature & salinity water, and low temperature & high salinity deep water (Fig. 1c). During the spring monsoon transition period, the water column was well stratified and quite stable, which is mainly attributed to weak wind-driven surface circulation compared to the monsoon period. Due to the well-stratified water column, the spring intermonsoon was considered to be the most oligotrophic period^53^.

### Taxonomic composition and characteristics

Samples of living coccolithophores from the EEIO during the spring intermonsoon period yielded 26 species. SEM photographs have shown some of the selected species in Plates I-V, including several predominant taxa. Among coccolith, *Gephyrocapsa oceanica*, *Emiliania huxleyi*, *Umbilicosphaera sibogae*, *Helicosphaera carteri*, and *H. hyalina* were most dominant. Coccosphere assemblages were dominated by *G. oceanica*, *Florisphaera profunda*, *E. huxleyi*, *Umbellosphaera irregularis*, and *U. sibogae*. *G. oceanica* was overwhelmingly dominant among the coccoliths, with occurrence frequency and relative abundance up to 96.5% and 71.76%, respectively. *G. oceanica* and *E. huxleyi* has high frequencies, with 44.5% and 31%, respectively. *F. profunda* has the highest (up to 40.78%) relative abundance (Supplementary Tables S2 and S3).

Coccolith and coccosphere density ranged from 0.19 × 10^3^ to 161.71 × 10^3^ coccoliths l^−1^ and 0.19 × 10^3^ to 68.37 × 10^3^ cells l^−1^, averaged at 22.66 × 10^3^ coccoliths l^−1^ and 9.39 × 10^3^ coccoliths l^−1^, respectively. The most predominant coccolith *G*. *oceanica* was ranged as 0~154.96 × 10^3^ coccoliths l^−1^, with a mean value of 16.26 × 10^3^ coccoliths l^−1^. The most predominant coccosphere was represented as *F*. *profunda*, which has its abundance ranged 0~53.85 × 10^3^ cells l^−1^, with the average value 3.83 × 10^3^ cells l^−1^ (Supplementary Table S2). The abundances of five dominant coccolith and five taxa of coccosphere were shown in Supplementary Fig. S1. The other dominant coccolith has similar abundances. For the remaining coccosphere, *G*. *oceanica* and *U*. *irregularis* were noted as more abundant than *E*. *huxleyi* and *U*. *sibogae*.

### Distribution and diversity pattern

**Figure 2:**
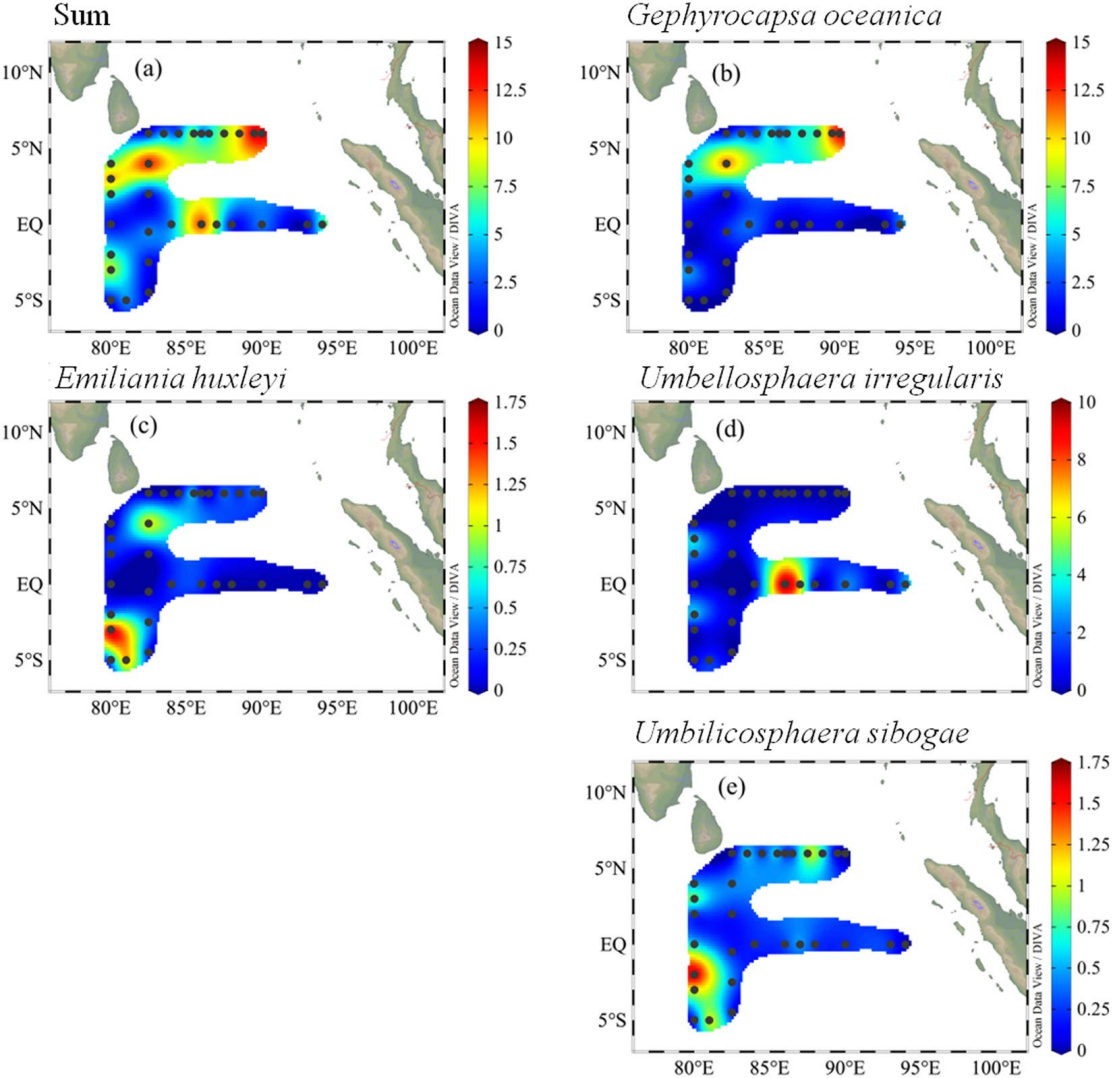
The surface distribution of dominant coccospheres (units: ×10^3^ cells l^−1^) in the surveyed area. Plotted using Ocean Data View (ODV) 4.7.6 software (https://odv.awi.de/en/).

The *H*′ and *J* values for coccospheres were slightly higher than the corresponding values of coccoliths (Supplementary Fig. S10). The horizontal distributions of dominant coccoliths and coccospheres were shown in Supplementary Figs S2 and 3. The greatest abundance of coccolith was noticed in three regions: south of Sri Lanka, easternmost Sri Lanka, and southernmost area. There was a peculiar oceanographic phenomenon at St. I316 characterized by surface lowest temperature and highest salinity, where the coccoliths of *U. sibogae* and *H. carteri* were predominant. Abundance was relatively low in the equatorial region. In contrast to the coccoliths, coccospheres were more homogeneous in their horizontal distributions (Fig. 2).

**Figure 3:**
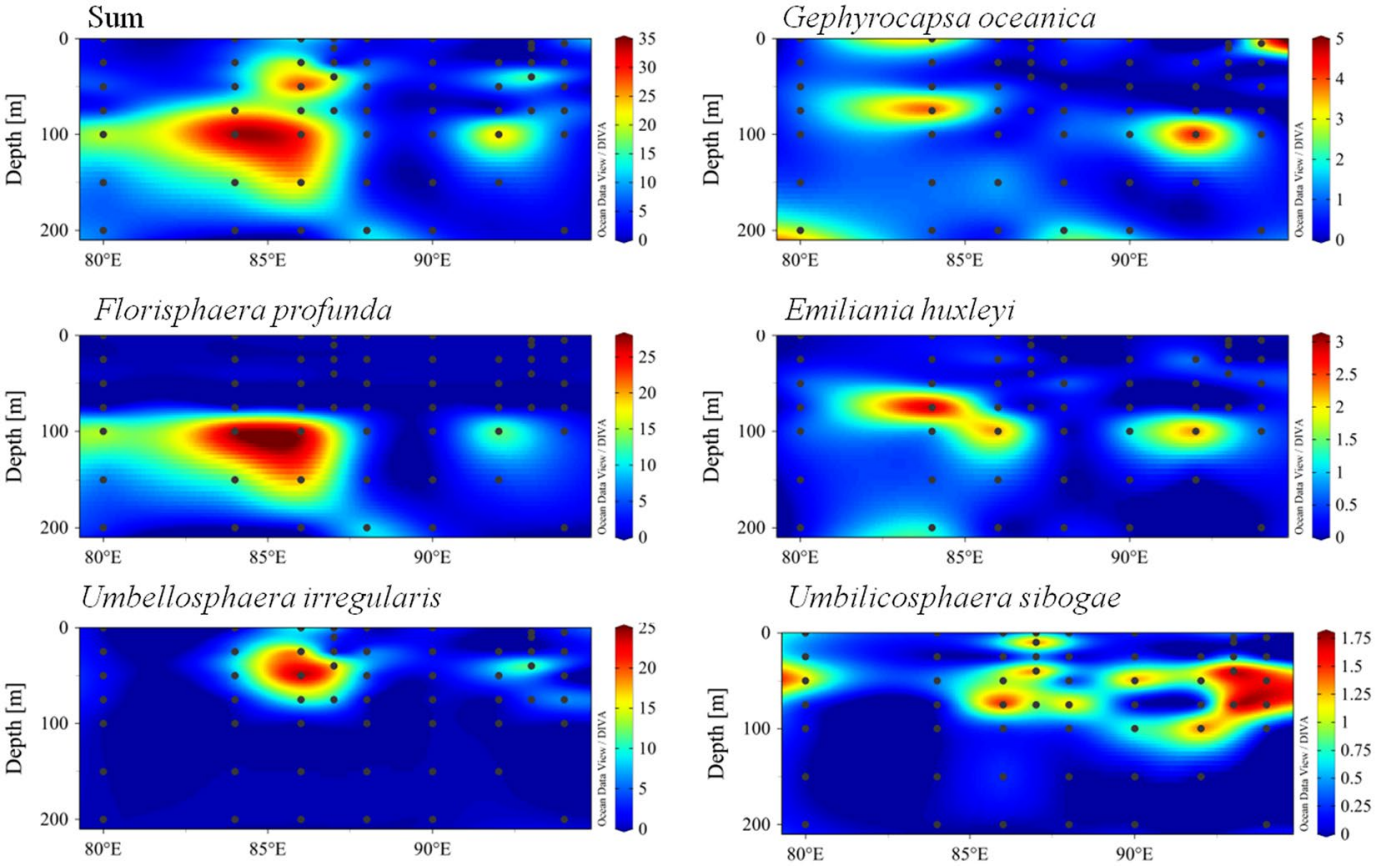
Dominant coccosphere distributions (units: ×10^3^ cells l^−1^) along section A of the surveyed area. Plotted using Ocean Data View (ODV) 4.7.6 software (https://odv.awi.de/en/).

Dominant coccolithophores abundances along two sections were illustrated in Figs S3–S5. More abundant coccolith was restricted to the water column west of 90°E (Supplementary Fig. S3). Nearly no coccoliths were distributed from the surface down to 50 m along east of 90°E. Dominant coccospheres abundance in section A were mainly represented by *F*. *profunda* and *U*. *irregularis* (Fig. 3). These two taxa followed trends similar to the coccoliths. For section B, coccolith abundance was primarily contributed by *G*. *oceanica* (Sup. Fig. S4) and abundance was concentrated in the easternmost region. *E*. *huxleyi* and *U*. *sibogae* were mainly distributed in deeper water. *H*. *hyalina* abundance decreased in deeper and open water and *H*. *carteri* showed a patchiness pattern. Supplementary Fig. S5 showed obvious coccosphere abundance in the 75 m water layer of section B, where a deep abundance maximum was located. *F*. *profunda* was the dominant coccosphere in the assemblage at section B.

**Figure 4:**
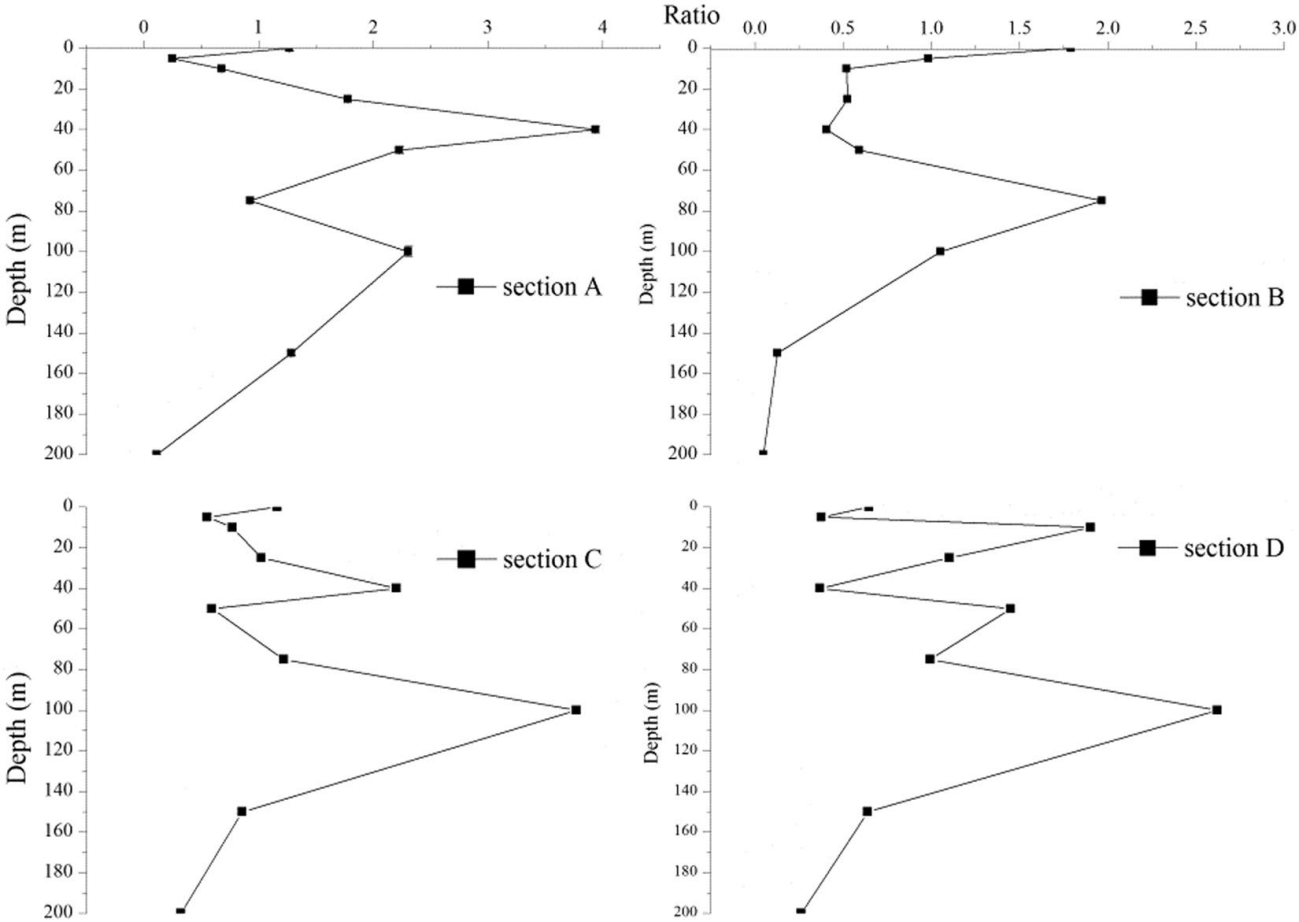
The ratio of coccosphere to free coccolith in upper ocean column in the surveyed area. (**a**) section A; (**b**) section B; (**c**) section C; (**d**): section D. Plotted using Origin 8.5 PRO software (http://www.originlab.com/).

Vertically, the dominant coccoliths were confined to the middle layer (25 m–75 m) of EEIO (Supplementary Fig. S6). Most of them reached peak values at the 50 m water layer, except for *E*. *huxleyi* and *H*. *carteri*, which peak values were located in the 200 m and 100 m water layers. Coccosphere were increased from the surface towards the middle water and then decreased towards the bottom water (Sup. Fig. S7). The ratios between coccospheres and free coccoliths were charted vertically through the depth profiles (Fig. 4). The ratio values basically coincided with coccosphere abundance. The ratio reached a maximum at 40 m layer along sections A and C. The ratio along section B exhibited a differed trend and its maximum was present at the surface layer. The ratio along section D was similar to that along section C. We presumed that coccospheres disintegrated into coccoliths after sinking at a short distance, then the coccoliths dissolved as the depths increased to about 100 m and the pH decreased. The ratio decreased to its minimum, 0.03 at a depth of 200 m, where attenuation of photosynthetically active radiation is estimated to have been 1%, which is unfavourable for the coccosphere proliferation.

### PIC, POC, and rain ratios

The mean PIC, POC, and rain ratios were 0.002~10.008, 0.498 μgC l^−^, 0.001~6.100, 1.047 μgC l^−1^, and 0.093~9.439, 0.990, respectively. The surface distributions and depth-integrated patterns of PIC, POC, and rain ratio were shown in Supplementary Fig. S8. We found a dominance of *Oolithotus fragilis* and *G*. *oceanica* in the biogenic PIC. Unlike PIC, POC was mainly contributed by cells of *U*. *sibogae* and *U*. *irregularis*. The pattern of PIC and POC appeared to be similar. The surface water around Sri Lanka section displayed two peaks. In the case of the integral value, PIC and POC were preferentially distributed to the west of 90°E. The depth averaged-rain ratio peak occurred at 80°E–85°E (Sup. Fig. S8).

Along section A, *O*. *fragilis* contributed about 48% of total PIC, with a maximum value at St. I405 accounting for 94%. The POC distribution pattern was similar to *U*. *irregularis* abundance. The maximum rain ratio value occurred east of 90°E. Along section B, PIC was represented by *F*. *profunda*. POC and cell abundance showed concurrent trends. Rain ratio had a clear pattern with higher values in the northern surface water and bottom layers.

### Coccosphere clustering and analysis

**Figure 5:**
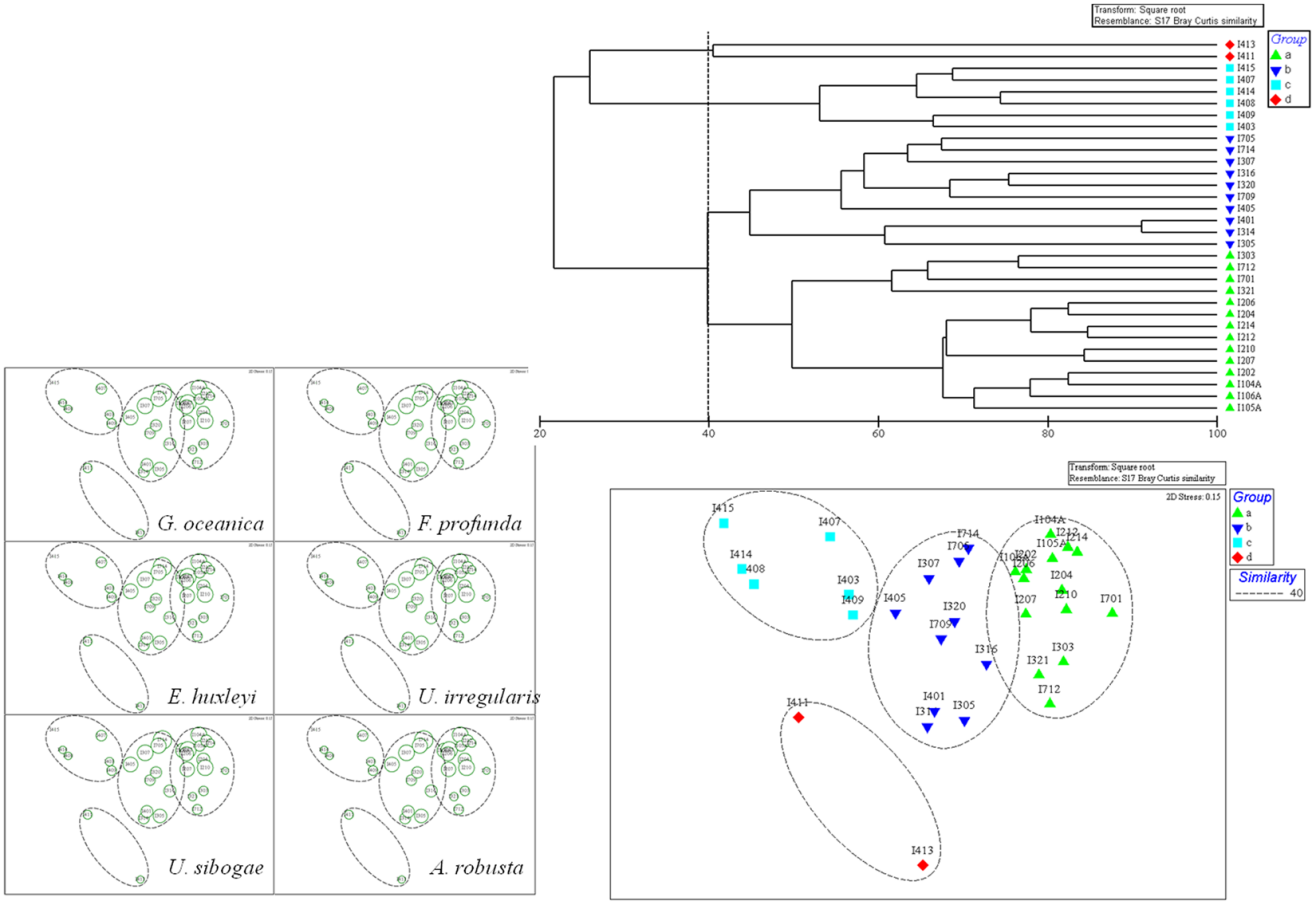
Stations clustered by Bray-Curtis rank similarities and group average linkage (upper): 4 groups were clustered; MDS ordination and its bubble plots for six dominant coccospheres with varied distributions in 4 groups (below). Plotted using PRIMER 6.0 software (Plymouth Routines In Multivariate Ecological Research, developed at the Plymouth Marine Laboratory, United Kingdom, http://www.primer-e.com/).

Coccosphere samples at 75 m layer (Deep Chlorophyll Maximum, DCM), where great quantities of coccosphere located, were chosen for the cluster and MDS analysis. The combinations of clustering technique and MDS method are usually conducive to obtain balanced and reliable conclusions in ecological studies^54,55^. All samples could be clustered into four groups (Group a, b, c, d). MDS stress values (0.15) lesser than 0.2 give an useful ordination picture, particularly at the lower end of this range^55,56^. ANOSIM analysis revealed remarkable difference (Global R = 0.85, p = 0.001) among group classification with the exception of Group b-d and Group c-d whose R value < p value^57^. It is accepted that Global R-value larger than 0.5 accounts for significant difference among groups^58^. Apparently, localities were basically classified along transects (e.g. Group c included the equatorial localities), whereas some exceptions existed (Fig. 5). Besides, MDS bubble plots for first six dominant coccospheres were presented in Fig. 5. It is apparently stated that the Groups a and b were mainly composed by dominant coccosphere *G*. *oceanica*, *F*. *profunda*, *E*. *huxleyi* and *A*. *robusta*. While Group c was primarily contributed by species *U*. *sibogae* and *U*. *irregularis*. Considering Group d only contained two localities, *G*. *oceanica* dominated the whole group. The SIMPER results were shown in Supplementary Table S4. It showed that the contribution rate of the dominant was coccospheres in each group.

## Discussion

**Figure 6:**
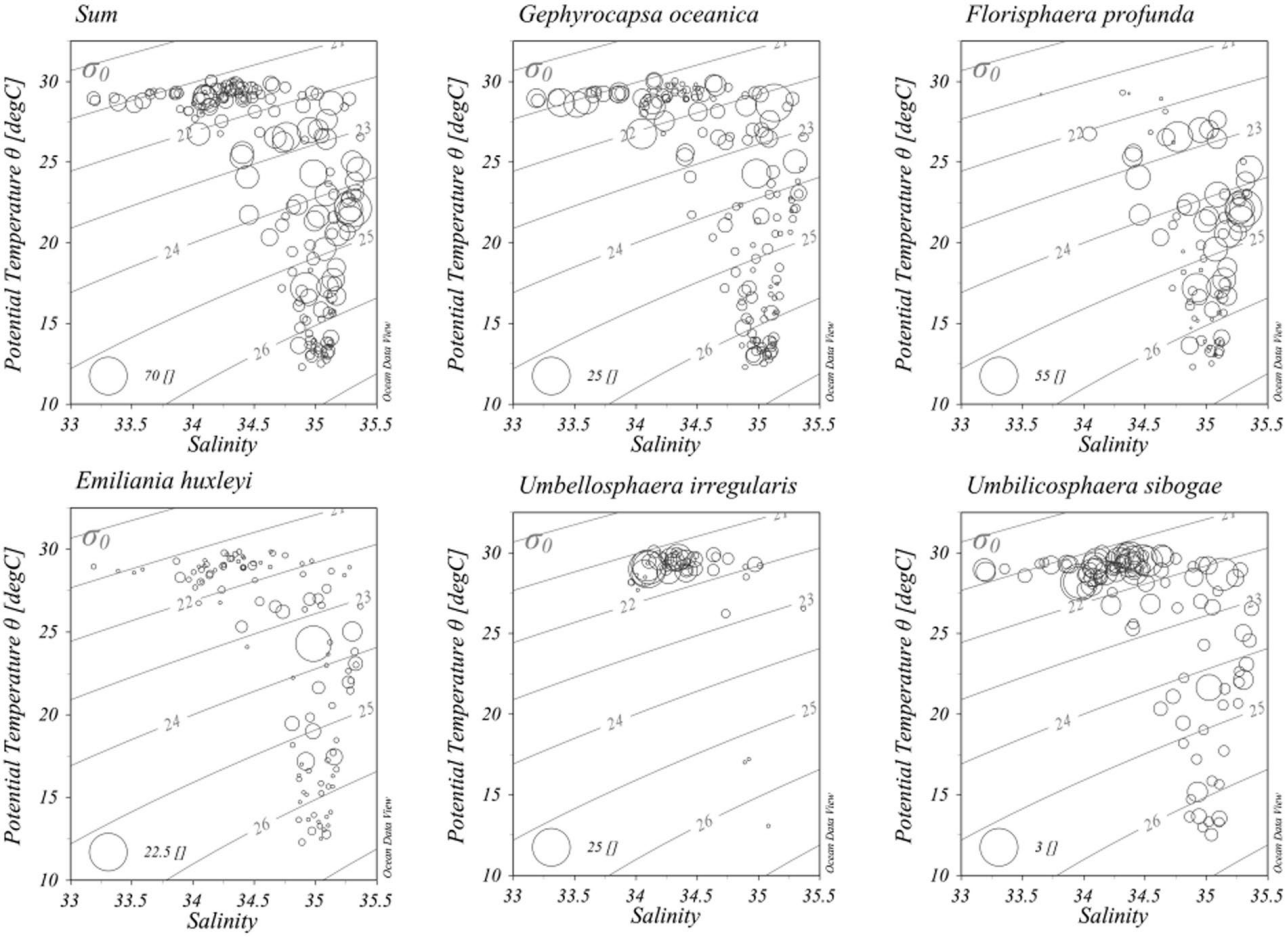
Scatter plots of coccosphere distribution under T-S properties in the surveyed area. Plotted using Ocean Data View (ODV) 4.7.6 software (https://odv.awi.de/)

**Figure 7:**
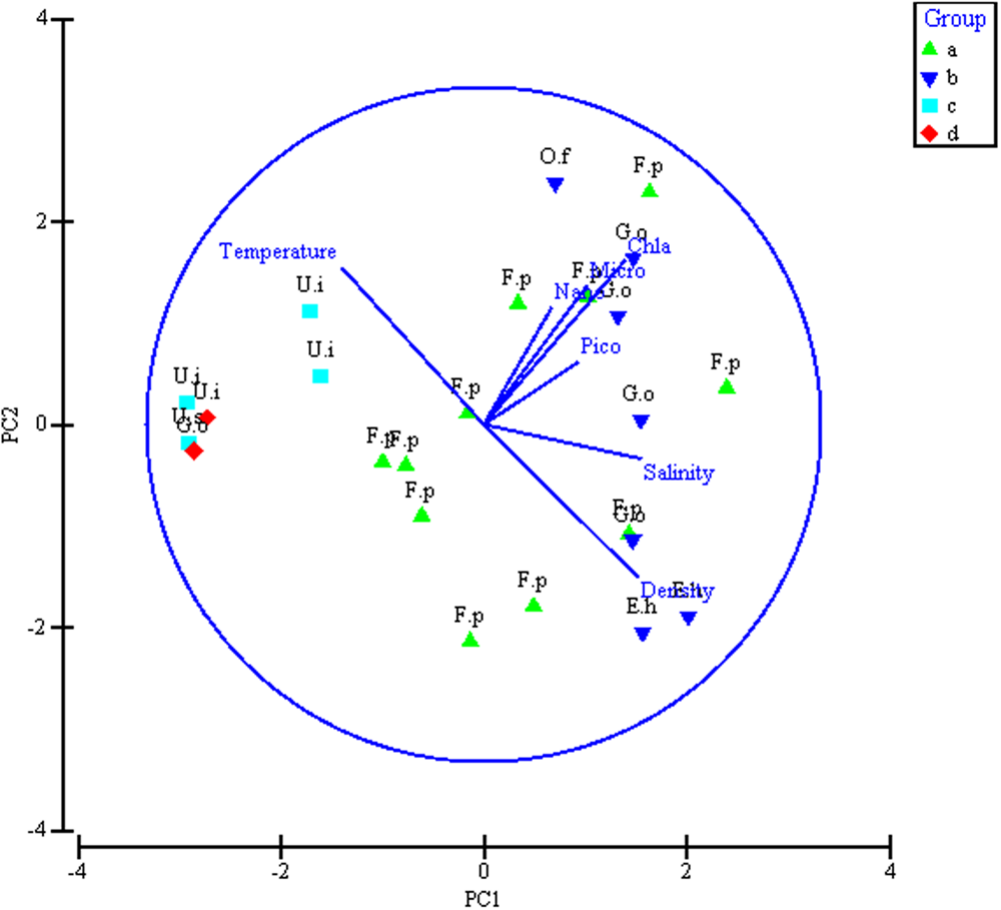
Ordination biplot based on PCA analysis between coccosphere and environmental variables of the surveyed area. Note: group partitions here refer to Supplementary Fig. S8; Chla: chlorophyll, Micro: micro-sized Chla, Nano: nano-sized Chla, Pico: Pico-sized Chla, G.o:*Gephyrocapsa oceanica*, F.p: *Florisphaera profunda*, E.h: *Emiliania huxleyi*, U.i: *Umbellosphaera irregularis*, U.s: *Umbilicosphaera sibogae*, A.r: *Algirosphaera robusta*. Plotted using PRIMER 6.0 software (Plymouth Routines In Multivariate Ecological Research, developed at the Plymouth Marine Laboratory, United Kingdom, http://www.primer-e.com/).

The surface water of eastern Sri Lanka (around St. I 104 A) had the greatest coccolith and coccosphere richness and abundance. The biodiversity indices were much lower around the waters of Sri Lanka (Sup. Fig. S9), suggesting that the local water in that system has lacked ecosystem stability. Therefore, coccosphere aggregations exhibited more diversity than coccoliths. This finding was consistent with that of Guptha *et al*.^6^. The physical distributions of coccolithophore assemblages in relation to the temperature-salinity were also shown (Figs 6 and 7). The coccoliths represented by *G*. *oceanica*, *U*. *sibogae*, *H*. *carteri* and *H*. *hyalina* were concentrated in the surface layer characterized by high temperature and low salinity. Furthermore, *E*. *huxleyi* was predominantly distributed in the intermediate layer with moderate temperature and salinity. The coccospheres, *F*. *profunda* and *E*. *huxleyi* were mainly found in the deeper euphotic layer where the DCM was located. *U*. *irregularis* and *U*. *sibogae* has greater abundances in the surface layer, confirming their preference for oligotrophic conditions. The peculiar oceanographic phenomenon at St. I316, characterized by the lowest surface temperature and the highest surface salinity, was occupied predominately by coccoliths of *U*. *sibogae* and *H*. *carteri* (Sup. Fig. S2). *F*. *profunda* was distributed only below 50 m at St. I316, indicating a stratified and stable water locally. This peculiar hydrology was therefore not caused by upwelling but may have been produced by lateral advection. It is very hard to identify what kinds of currents created this peculiar biophysical distribution after all, water currents are not prosperous during the intermonsoon. The POC pattern can be represented by coccosphere abundance. Varied allocation to calcification produced dissimilarities in the PIC/POC ratios. Large rain ratio values around Sri Lanka waters predicted a mineral ballast with a strong drawdown of biological carbon towards the deep seafloor^59,60^. We suggest that the rain ratio is of great importance in predicting biomineralization and photosynthetic production^12,61^.

Many coccolithophore indicator species were collected in this study although several were uncommon. *G*. *oceanica* is a representative dominant species that shows a preference for eutrophic water^62^. In the surface distribution of *G*. *oceanica*, both coccoliths and coccospheres were predominantly distributed in the easternmost waters of Sri Lanka. This may be due to the eutrophic water derived from the highly productive Andaman Sea which was linked to the Bay of Bengal through narrow channels^63,64^. The coccosphere of *U*. *irregularis* was only common in the equatorial zone, indicating oligotrophic water conditions overthere^65^. In the Indian Ocean, eight species of *Florisphaera* were discovered in deep water^66^. We found only one species of *Florisphaera* (*F*. *profunda*) and were typically occurred in the disphotic layer below at 100 m. As an inhabitant of deep water, *F*. *profunda* hardly occurred on the surface water layer unless associated with upwelling. Maxima of among the coccoliths of *U*. *sibogae* and *H*. *carteri* were found at St. I316 suggesting that these species showed affinities to low temperature and high salinity in water. The cosmopolitan taxa, *Calcidiscus leptoporus*, was detected and its coccoliths has peaked at a depth of 200 m at St. I705. *C*. *leptoporus* is sparsely distributed in the water column, whereas it predominates in the coccolithophore flora of the sediment owing to its resistance to disintegration^67^. Biogenic coccoliths are considered as an important carbon sink and experience different levels of dissolution in the context of varied hydrological condition^68^.

Coccolithophore abundance was relatively low during the low wind transition period compared to previous studies conducted during the monsoon period in the EEIO. The low abundance is due to the gentle associated with light winds and low nutrient availability during the spring intermonsoon season leading to low primary productivity and biomass in the EEIO^69^. The coccolithophores in surface water were most abundant in the northeast area where pockets of low-salinity water plume occur (Fig. 1). This resulted from the inflow of less saline water into the equatorial Indian Ocean from the Bay of the Bengal and Andaman Seas^70,71^. The outflows derived from the surface water of the Andaman Sea become concentrated between the south Nicobar Islands and Sumatra^72^. In contrast, a highly saline water tongue was observed along the equatorial Indian Ocean (west of 90°E), indicating that Wyrtki jets (WJs) prevailed during the spring intermonsoon period. There was consistency in the coccolithophore distribution pattern at the equator. The maximum abundance along the section west of 90°E was probably caused by inflow from WJs considering their ability to alter the oceanic layer structure. PCA was carried out to examine the relationships among the environmental variables, with the most abundant coccolithophores superposed on the PC1–PC2 hyperspace (Fig. 7). Coccolithophore abundance was driven primarily by temperature, salinity, density. The abundance of coccolithophorid phytoplankton will usually correspond to high Chl*a* levels. The clustering of environmental data from sample locations reflected the grouping of species data (except for a few isolated points). The most abundant species were shown above each locality symbol (Fig. 7). The first three principal components (PC1, PC2, PC3) were extracted based on eigenvalues larger than 1 and explained 42%, 24%, and 20.2% of the variation, respectively. The cumulative variances of the three components were reached up to 86.2% (PC3 not shown). The eigenvectors of all five principal components were shown in Supplementary Table S5. The results of PCA indicated that salinity, density, and pico-Chl*a* has a positive relationship with PC1, whereas a close correlation occurred in Group B that was dominated by *E*. *huxleyi* and *G*. *oceanica*. Similarly, temperature, Chl*a*, micro-Chl*a* and nano-Chl*a* were positively correlated to PC2. Groups C and D, characterized by *U*. *irregularis*, were associated with high temperature. The majority of localities in Group A (represented by *F*. *profunda*) were negatively related to Chl*a* and size-fractionated Chl*a*. Finally, the MDS ordination of coccosphere abundance and the PCA ordination of environmental variables are in good agreement. This high degree of matching in our study confirmed that the present explanatory variables^73^ are appropriate for explaining the biological response variables.

## Conclusions

The coccolithophore assemblage in the EEIO during the spring intermonsoon season was primarily comprised of the coccoliths (in order of mean abundance) such as *G*. *oceanica*, *E*. *huxleyi*, *U*. *sibogae*, *H*. *carteri*, and *H*. *hyalina* and the coccospheres *F*. *profunda*, *G*. *oceanica*, *E*. *huxleyi*, *U*. *irregularis*, and *U*. *sibogae* based on dominance index. The abundance of coccoliths and coccospheres ranged from 0.19 × 10^3^~161 × 10^3^ coccoliths l^−1^ and 0.19 × 10^3^~68 × 10^3^ cells l^−1^, with an average value of 23. × 10^3^ coccoliths l^−1^ and 9.4 × 10^3^ cells l^−1^, respectively. The mean values of the biogenic PIC, POC, and the rain ratio were 0.50 μg C l^−1^, 1.0 μg C l^−1^, and 0.10, respectively. From the ratio of coccosphere and free coccolith, we can see that coccolith experienced different levels of dissolution when transported to the deep water. The rain ratio was considered to be of great importance in predicting biomineralization and photosynthetic production so relative biovolume and carbon biomass were calculated and used to derive the values of PIC, POC and rain ratio.

The horizontal distributions of coccolithophores exhibited three patches: south of Sri Lanka, easternmost Sri Lanka, and southernmost area. An unusual phenomenon was observed at the surface water of St. I316. Vertically, coccoliths abundance was restricted to the water column west of 90°E, exactly consistent with WJs appearance region. The localities and coccosphere were ordered by MDS and all samples were clustered into four groups in the EEIO. The coccolithophore abundance in this study was relatively low and resulting from the weak winds and minimal nutrient upwelling compared to previous studies that were conducted during the summer or winter monsoon seasons. During the spring intermonsoon period, no significant oceanic circulation occurred in the EEIO except for WJs. We inferred that, in the study area, different coccolithophore species had specific environmental preferences. Thus, coccolithophore species are good indicators of oceanographic changes in the EEIO. PCA was used to study the correlation between environmental variables, indicating positive or negative relationships with nanofloral species. Coccosphere distribution was highly correlated to specific environmental variables. This was shown by the MDS ordination of response variables and PCA ordination of explanatory variables. Coccolithophores can be used as dynamic indicators of the upper ocean for their sensitivity to environmental changes. Obtaining knowledge of specific cellular physiological behaviour related to global change variables will be a future challenge Future studies are required involving laboratory experiments using axenic cultures of coccolithophores, and cell POC and other chemical parameters need to be measured to refine existing algorithms of POC:cell volume ratios, allowing better evaluation of *in situ* POC, PIC and other chemical parameters in the future.

## Materials and Methods

### Survey area and sampling strategy

**Figure 8:**
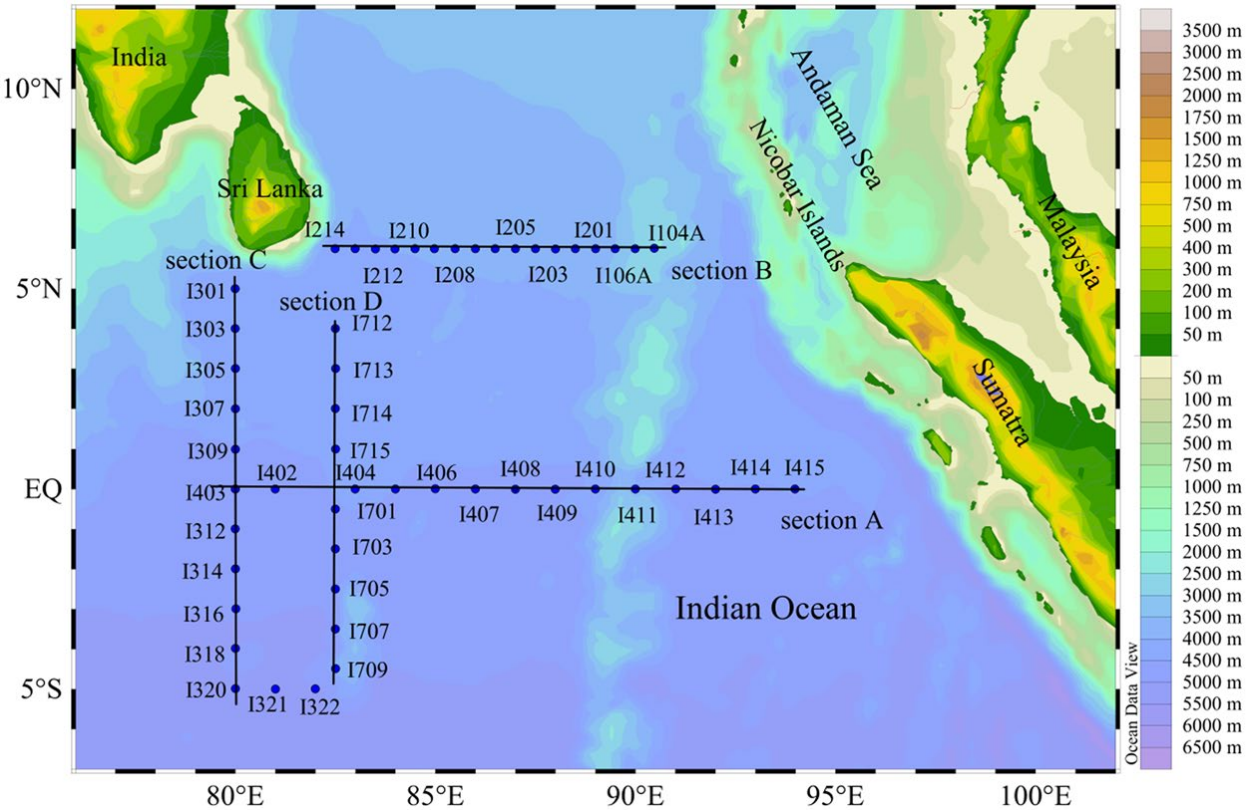
Study area in the eastern equatorial Indian Ocean showing the station locations. Plotted using Ocean Data View (ODV) 4.7.6 software (https://odv.awi.de/en/). Plates I–V.

An initial investigation cruise was conducted in the eastern equatorial Indian Ocean (EEIO) (80°~94°E, 6°N~5°S) (Fig. 8) onboard R/V “*Shiyan* 1” from March 10^th^ through April 9^th^, 2012. Seawater was collected at eight depths from the surface to 200 m using Niskin bottles on a rosette sampler (Sea-Bird SBE-911 Plus V2). At all the stations, temperature and salinity profile data were determined *in situ* with the attached sensors system (conductivity-temperature-depth, CTD) (Supplementary Table S1).

### Coccolithophore analysis

Coccolithophore samples 400–500 ml were filtered with a mixed cellulose membrane (25 mm, 0.22 μm) using a Millipore filter system connected to a vacuum pump under <100 mm Hg filtration pressure as soon as the seawater was collected onboard. After drying at room temperature in plastic Petri dishes, the filters were cut and subsequently mounted on glass slides with neutral balsam for a polarized microscope (Motic, BA300POL.) examination^74^. Totally at least 400 fields were counted by the standard of 30 coccospheres and 50 coccoliths were enumerated under a light microscope. The coccolithophore biomass (POC) was then calculated following the formula in Sun *et al*.^74^. One litter of seawater samples were gently filtered through 47 mm 0.45 μm polycarbonate filter for qualitative diagnosis under scanning electron microscope (SEM).

### Size-fractionated Chl*a* analysis

Chlorophyll *a* (Chl*a*) samples 800 ml were serially filtered using the same filtration system (vacuum <200 mm Hg) through 20 μm × 20 mm silk net (micro-class), 2 μm × 20 mm nylon membrane (nano-class) and 0.7 μm × 20 mm Whatman GF/F filters (pico-class). After filtration, Chl*a* membranes were immediately wrapped with aluminium foil and stored in a freezer −20 ed in alu and 0.7 μm × 20 mm and the measurements were made using the fluorescence method of Parsons *et al*.^75^. The primary data were displayed at (Supp. Table S6).

### Estimation of coccolith calcite, coccosphere carbon biomass

The cell size biovolume was evaluated from geometric models^76^ and then converted into carbon biomass (i.e. coccolithophore organic carbon, particulate organic carbon, POC, hereafter) using the formula of Eppley *et al*. and Guo *et al*.^77,78^. Cellular dimension was measured under SEM by scanning 20 individuals. Measured dimensions of most common species were found to be similar to those recorded in previous studies. Therefore, the determinations of common species calcite-CaCO_3_ (i.e. coccolithophore inorganic carbon, particulate inorganic carbon, PIC, hereafter) masses were based on k_s_ values (shape factor) and maximum length (diameter, μm) were recorded in previous studies^79,80^. The PIC/POC value is a potential rain ratio, which expresses the carbonate flux export to the outside of the euphotic zone. As for the irregularly shaped coccolithophores which biovolume has rare records, nearly 33% of the species (e.g. *Michaelsarsia elegans*, *Reticulofenestra sessilis*) were estimated with geometric models using SEM pictures from the literature, websites, and this study^47,81–83^. The website can be access from: http://ina.tmsoc.org/Nannotax3/index.html. It is noted that organic carbon was calculated with the exception of *Gladiolithus flabellatus* and *Reticulofenestra sessilis* by the reason of insufficient records from SEM data.

### Multivariate analysis

Box-whisker plots were prepared by the Golden Software Grapher 10.3.825 (LLC, Colorado, USA) (https://support.goldensoftware.com/hc/en-us/categories/115000653847-Grapher). Cluster analysis and non-metric multidimensional scaling^84^ on coccosphere data (after square root transformation) were simultaneously implemented using the program package PRIMER 6.0 (Plymouth Routines In Multivariate Ecological Research, developed at the Plymouth Marine Laboratory, United Kingdom, http://www.primer-e.com/). Prior to the above operations, the raw data were square root transformed. Then, principal component analysis (PCA) considering Euclidean distance was employed after data transformation and normalization. Significance testing was performed using the Analysis of Similarities (ANOSIM). In the Similarity Percentages-Species Contributions that the Percentages Routine (SIMPER) program was used for evaluating the contribution of each species to their sample group. All analyses were conducted to visualize the relations between the data abundance of phytoplankton and specific environmental factors. The spatial distribution of coccolithophores and hydrologic data were analyzed using freeware package Ocean Data View (ODV) 4.7.6 (https://odv.awi.de/)^85^.

### Coccolithophore identification guiding lines

The coccolithophore identification is principally guided by the rules and features of light microscopic pictures and scanning electronic microscopic pictures of published references^82,83,86^, and the specialized website http://www.mikrotax.org/Nannotax3/index.php?dir=Coccolithophores. Also, the species are classified based on the four general niches of coccolithophore: upwelling water species, oligotrophic water species, deep water dwellers, and miscellaneous species^87,88^.

## Electronic supplementary material

Supplementary material
