# Supplementary material for: Distribution of living coccolithophores in eastern Indian Ocean during spring intermonsoon
Source: Sci Rep. 2018 Aug 21;8:12488. doi: 10.1038/s41598-018-29688-w (PMC6104084; doi:10.1038/s41598-018-29688-w)
Supplement: Supplementary file 1 — Supplementary material [file 41598_2018_29688_MOESM1_ESM.docx]

**Scientific Reports**

**Supple information**

**Distribution of living coccolithophores in eastern Indian Ocean during spring intermonsoon**

Haijiao Liu, Jun Sun, Dongxiao Wang, Xiaodong Zhang, Cuixia Zhang, Shuqun Song^5^, Satheeswaran Thangaraj^2, 3^

**Supplementary Figures S1-S11 Tables S1-S6 Plates** **Ⅰ-Ⅴ**

**Supplementary Figure S1**

The abundance of dominant coccolithophore species in the eastern equatorial Indian Ocean. (units: coccoliths l^-1^, cells l^-1^). Plotted for Golden Software Grapher 10.3.825 software (LLC, Colorado, USA) (https://support.goldensoftware.com/hc/en-us/categories/115000653847-Grapher)

**Supplementary Figure S2**


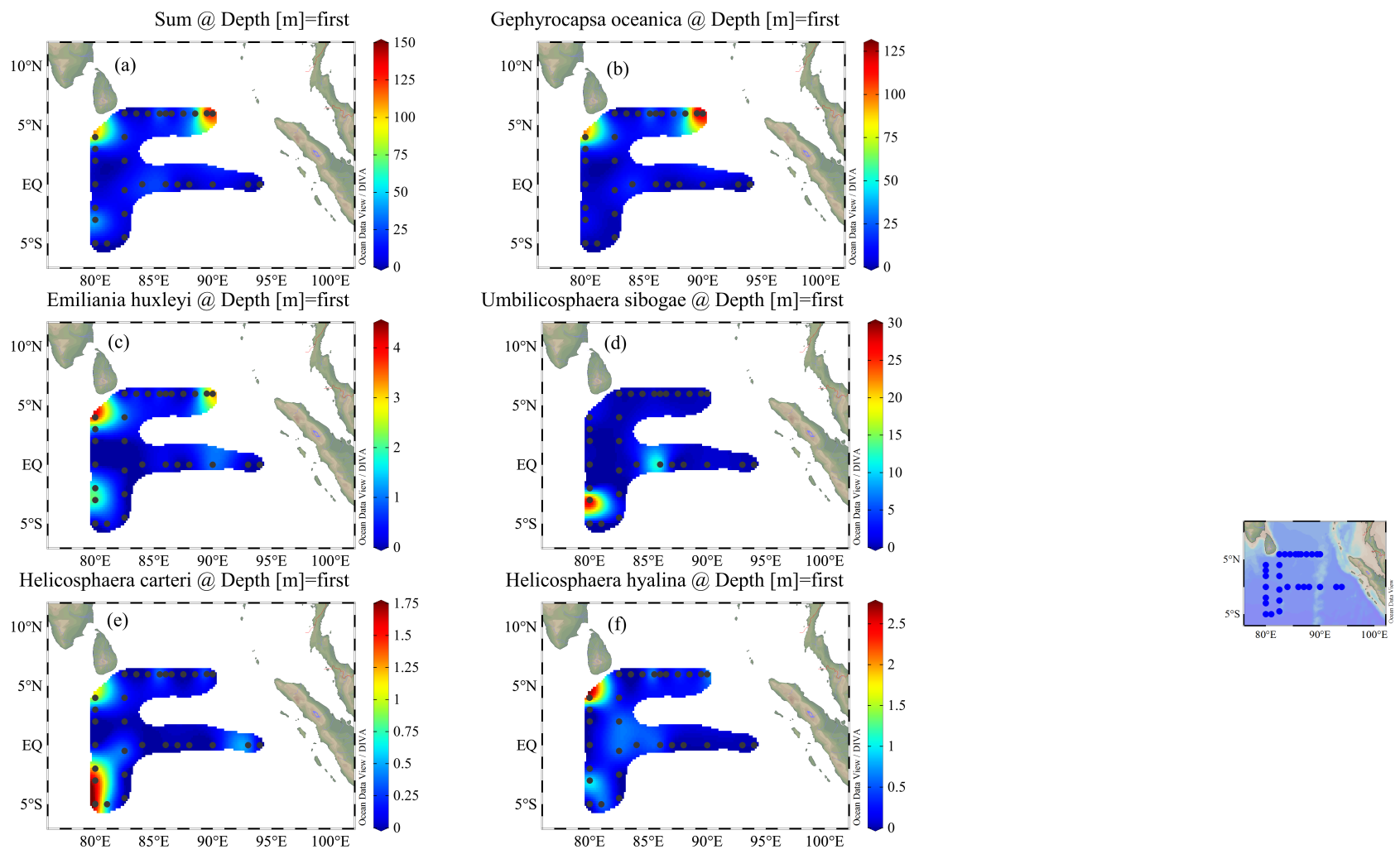


The surface distribution of dominant coccoliths (units: ×10^3^ coccoliths l^-1^) in the surveyed area. Plotted for Ocean Data View (ODV) 4.7.6 software (https://odv.awi.de/en/)

**Supplementary Figure S3**


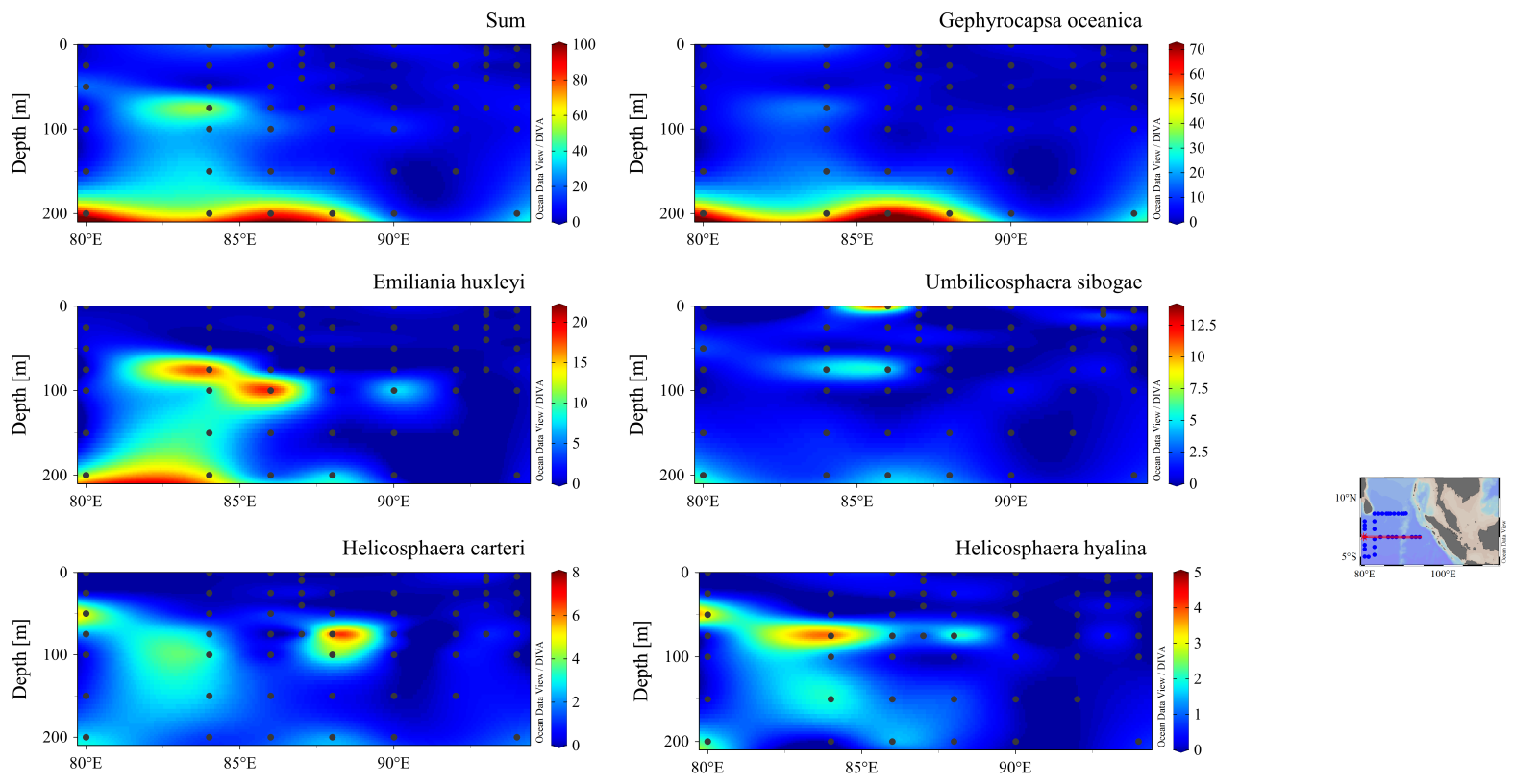


Dominant coccolith distributions (units: ×10^3^ coccoliths l^-1^) along section A of the surveyed area. Plotted for Ocean Data View (ODV) 4.7.6 software (https://odv.awi.de/en/)

**Supplementary Figure S4**


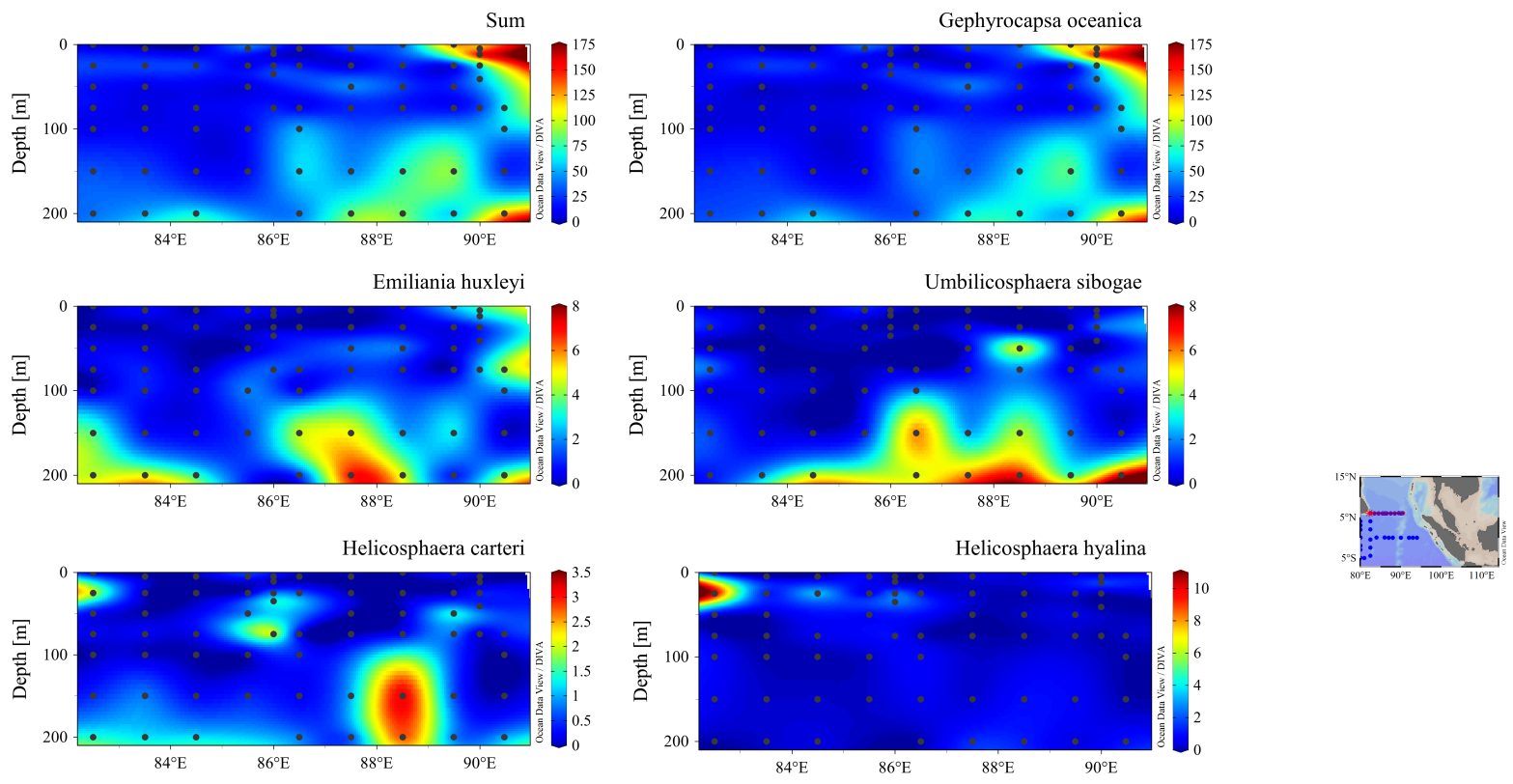


Dominant coccolith distributions (units: ×10^3^ coccoliths l^-1^) along section B of the surveyed area. Plotted for Ocean Data View (ODV) 4.7.6 software (https://odv.awi.de/en/)

**Supplementary Figure S5**


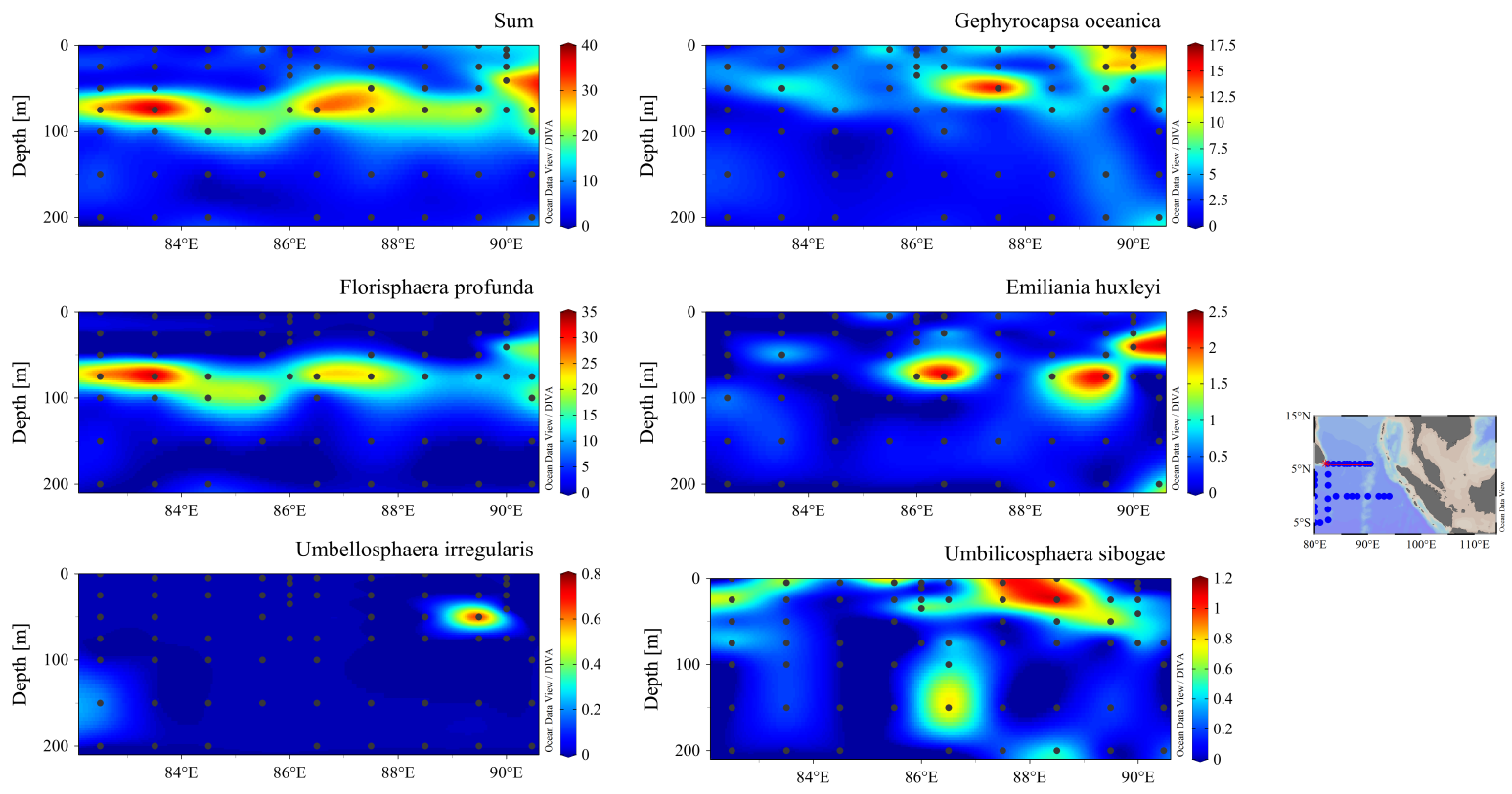


Dominant coccosphere distributions (units: ×10^3^ cells l^-1^) along section B of the surveyed area. Plotted for Ocean Data View (ODV) 4.7.6 software (https://odv.awi.de/en/)

**Supplementary Figure S6**

Vertical distributions of dominant coccoliths (units: coccoliths l^-1^) in the surveyed area. (a) Sum; (b) *Gephyrocapsa oceanica*; (c) *Emiliania huxleyi*; (d) *Umbilicosphaera sibogae*; (e) *Helicosphaera carteri*; (f) *Helicosphaera hyaline.* Plotted for Golden Software Grapher 10.3.825 software (LLC, Colorado, USA) (https://support.goldensoftware.com/hc/en-us/categories/115000653847-Grapher)

**Supplementary Figure S7**

Vertical distributions of dominant coccospheres (units: cells l^-1^) in the surveyed area. (a) Sum; (b) *Gephyrocapsa oceanica*; (c) *Florisphaera profunda*; (d) *Emiliania huxleyi*; (e) *Umbellosphaera irregularis*; (f) *Umbilicosphaera sibogae*. Plotted for Golden Software Grapher 10.3.825 software (LLC, Colorado, USA) (https://support.goldensoftware.com/hc/en-us/categories/115000653847-Grapher)

**Supplementary Figure S8**


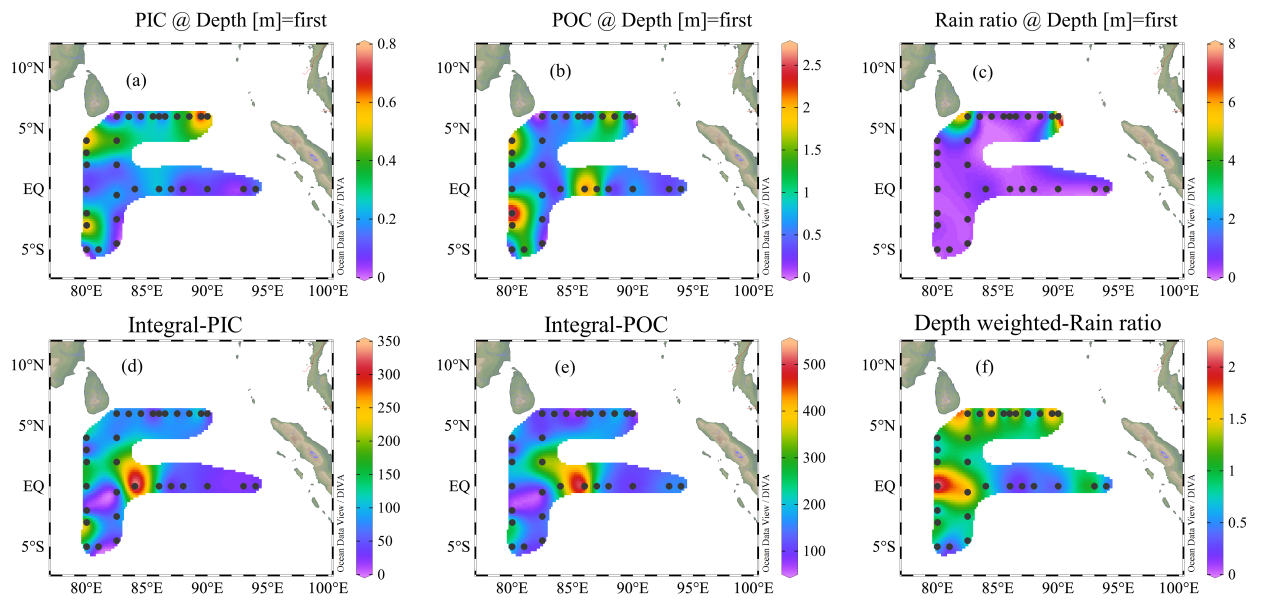


The horizontal distributions of PIC, POC (units: μgC l^-1^), and rain ratio in the surveyed area. (a)~(c): of surface layer; (d)~(f): of vertically integrated. Plotted for Ocean Data View (ODV) 4.7.6 software (https://odv.awi.de/en/)

**Supplementary Figure S9**


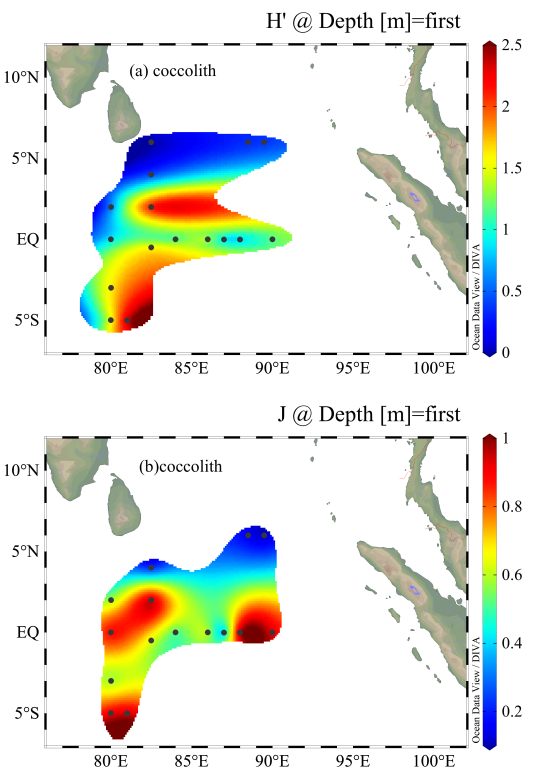

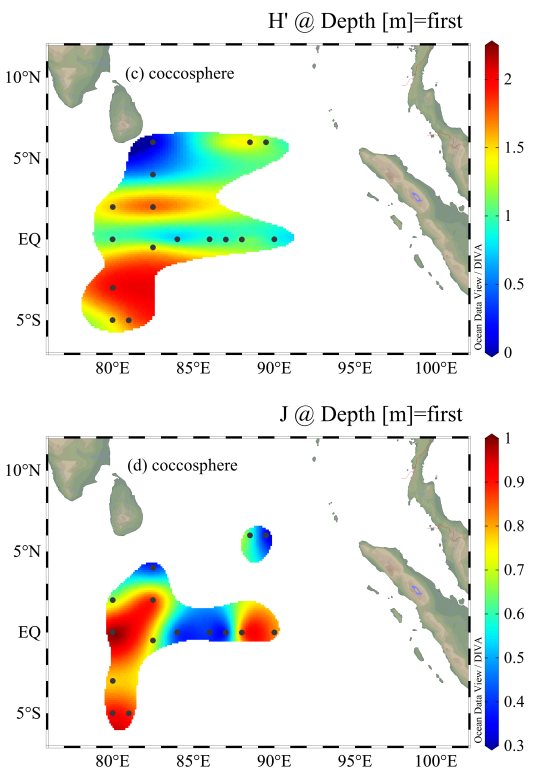


Surface distributions of biodiversity index of coccolithophore in the surveyed area. Plotted for Ocean Data View (ODV) 4.7.6 software (https://odv.awi.de/en/)

**Supplementary Figure S10**

Box and whisker diagrams of biodiversity index of coccolithophores amd coccoliths in the surveyed area. Plotted for Golden Software Grapher 10.3.825 software (LLC, Colorado, USA) (https://support.goldensoftware.com/hc/en-us/categories/115000653847-Grapher)

**Supplementary Figure S11**


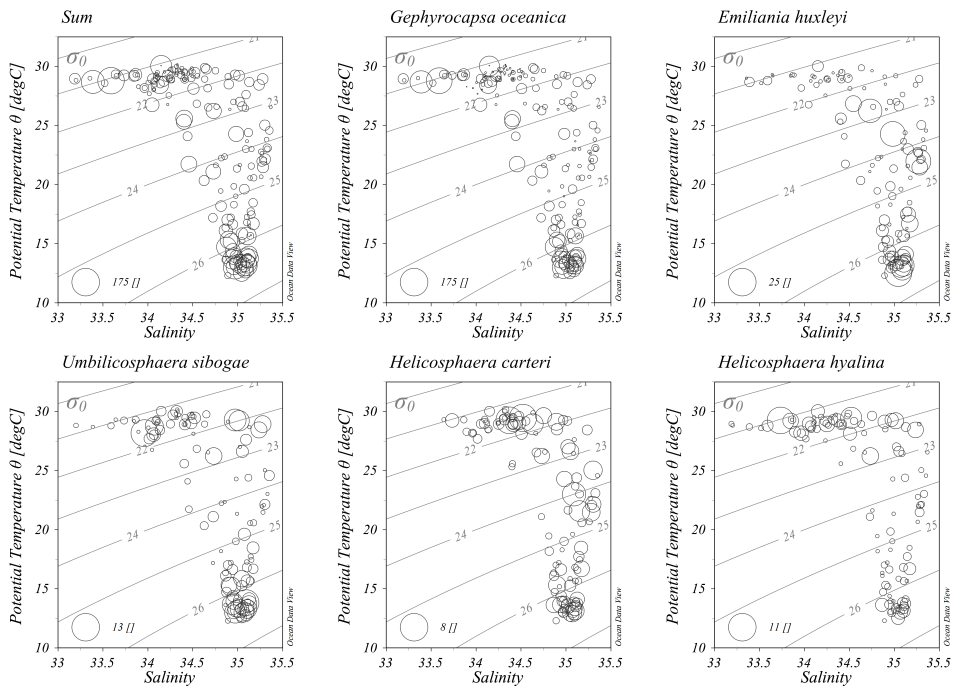


Scatter plots of coccolith distribution under T-S properties in the surveyed area. Plotted for Ocean Data View (ODV) 4.7.6 software (https://odv.awi.de/en/)**Supplementary Table S1**

| Date | Station | Longitude [degrees East] | Latitude [degrees North] | Bot. Depth [m] | Depth [m] | Salinity | Temperature |
| --- | --- | --- | --- | --- | --- | --- | --- |
| 2012/3/10 | I104A | 90.474 | 6.013 | 2645 | 0 |  |  |
| 2012/3/10 | I104A | 90.474 | 6.013 | 2645 | 25 | 33.865 | 28.8777 |
| 2012/3/10 | I104A | 90.474 | 6.013 | 2645 | 75 | 34.4055 | 25.5934 |
| 2012/3/10 | I104A | 90.474 | 6.013 | 2645 | 100 | 34.4572 | 21.751 |
| 2012/3/10 | I104A | 90.474 | 6.013 | 2645 | 150 | 34.7253 | 17.2092 |
| 2012/3/10 | I104A | 90.474 | 6.013 | 2645 | 200 | 34.97 | 13.0031 |
| 2012/3/11 | I105A | 89.998 | 5.999 | 2800 | 5 | 33.4117 | 28.7505 |
| 2012/3/11 | I105A | 89.998 | 5.999 | 2800 | 25 | 33.6372 | 28.7741 |
| 2012/3/11 | I105A | 89.998 | 5.999 | 2800 | 75 | 34.3554 | 24.7787 |
| 2012/3/11 | I105A | 89.998 | 5.999 | 2800 | 100 | 34.6332 | 21.1659 |
| 2012/3/11 | I105A | 89.998 | 5.999 | 2800 | 150 | 34.8027 | 15.6913 |
| 2012/3/11 | I105A | 89.998 | 5.999 | 2800 | 200 | 34.9944 | 12.7898 |
| 2012/3/11 | I105A | 89.998 | 5.999 | 2800 | 5 | 33.389 | 28.6959 |
| 2012/3/11 | I105A | 89.998 | 5.999 | 2800 | 12 | 33.5904 | 28.7918 |
| 2012/3/11 | I105A | 89.998 | 5.999 | 2800 | 25 | 34.1338 | 28.4461 |
| 2012/3/11 | I105A | 89.998 | 5.999 | 2800 | 41 | 34.3991 | 25.325 |
| 2012/3/11 | I105A | 89.998 | 5.999 | 2800 | 75 | 34.6267 | 20.3538 |
| 2012/3/11 | I106A | 89.493 | 6.002 | 3244 | 0 |  |  |
| 2012/3/11 | I106A | 89.493 | 6.002 | 3244 | 25 | 33.5188 | 28.5854 |
| 2012/3/11 | I106A | 89.493 | 6.002 | 3244 | 50 | 34.9004 | 28.5108 |
| 2012/3/11 | I106A | 89.493 | 6.002 | 3244 | 75 | 34.6719 | 26.5575 |
| 2012/3/11 | I106A | 89.493 | 6.002 | 3244 | 100 | 34.491 | 22.1867 |
| 2012/3/11 | I106A | 89.493 | 6.002 | 3244 | 150 | 34.8745 | 14.7398 |
| 2012/3/11 | I106A | 89.493 | 6.002 | 3244 | 200 | 34.9594 | 13.0703 |
| 2012/3/11 | I201 | 89.007 | 6.001 | 3826 | 5 | 33.6147 | 28.8025 |
| 2012/3/11 | I201 | 89.007 | 6.001 | 3826 | 25 | 34.8378 | 28.9808 |
| 2012/3/11 | I201 | 89.007 | 6.001 | 3826 | 50 | 34.8695 | 27.9141 |
| 2012/3/11 | I201 | 89.007 | 6.001 | 3826 | 75 | 34.4749 | 25.5118 |
| 2012/3/11 | I201 | 89.007 | 6.001 | 3826 | 100 | 34.8283 | 19.9067 |
| 2012/3/11 | I201 | 89.007 | 6.001 | 3826 | 150 | 34.9223 | 14.8952 |
| 2012/3/11 | I201 | 89.007 | 6.001 | 3826 | 200 | 34.9953 | 13.0367 |
| 2012/3/12 | I202 | 88.502 | 6 | 3880 | 0 |  |  |
| 2012/3/12 | I202 | 88.502 | 6 | 3880 | 25 | 34.179 | 28.744 |
| 2012/3/12 | I202 | 88.502 | 6 | 3880 | 50 | 35.278 | 28.9322 |
| 2012/3/12 | I202 | 88.502 | 6 | 3880 | 75 | 35.0913 | 27.6402 |
| 2012/3/12 | I202 | 88.502 | 6 | 3880 | 100 | 34.8946 | 21.8578 |
| 2012/3/12 | I202 | 88.502 | 6 | 3880 | 150 | 34.9032 | 15.3358 |
| 2012/3/12 | I202 | 88.502 | 6 | 3880 | 200 | 34.9423 | 13.6941 |
| 2012/3/12 | I203 | 88.006 | 5.995 | 3886 | 5 | 33.3106 | 29.1733 |
| 2012/3/12 | I203 | 88.006 | 5.995 | 3886 | 25 | 34.0496 | 28.0342 |
| 2012/3/12 | I203 | 88.006 | 5.995 | 3886 | 50 | 35.0485 | 28.5894 |
| 2012/3/12 | I203 | 88.006 | 5.995 | 3886 | 75 | 34.9327 | 26.055 |
| 2012/3/12 | I203 | 88.006 | 5.995 | 3886 | 100 | 34.6629 | 19.393 |
| 2012/3/12 | I203 | 88.006 | 5.995 | 3886 | 150 | 34.9652 | 15.5011 |
| 2012/3/12 | I203 | 88.006 | 5.995 | 3886 | 200 | 35.0085 | 13.5029 |
| 2012/3/12 | I203 | 88.006 | 5.995 | 3886 | 0 |  |  |
| 2012/3/13 | I204 | 87.502 | 6 | 3909 | 5 | 33.1892 | 28.9563 |
| 2012/3/13 | I204 | 87.502 | 6 | 3909 | 25 | 33.2001 | 28.8208 |
| 2012/3/13 | I204 | 87.502 | 6 | 3909 | 50 | 34.0468 | 26.7609 |
| 2012/3/13 | I204 | 87.502 | 6 | 3909 | 75 | 34.4432 | 24.1037 |
| 2012/3/13 | I204 | 87.502 | 6 | 3909 | 100 | 34.6103 | 19.9297 |
| 2012/3/13 | I204 | 87.502 | 6 | 3909 | 150 | 34.8671 | 16.124 |
| 2012/3/13 | I204 | 87.502 | 6 | 3909 | 200 | 35.0206 | 13.3528 |
| 2012/3/13 | I205 | 87.001 | 6.001 | 3885 | 5 | 33.1655 | 29.11 |
| 2012/3/13 | I205 | 87.001 | 6.001 | 3885 | 25 | 33.966 | 28.5005 |
| 2012/3/13 | I205 | 87.001 | 6.001 | 3885 | 50 | 35.0035 | 28.4943 |
| 2012/3/13 | I205 | 87.001 | 6.001 | 3885 | 75 | 35.1959 | 25.2175 |
| 2012/3/13 | I205 | 87.001 | 6.001 | 3885 | 100 | 34.7113 | 21.357 |
| 2012/3/13 | I205 | 87.001 | 6.001 | 3885 | 150 | 34.8605 | 16.1891 |
| 2012/3/13 | I205 | 87.001 | 6.001 | 3885 | 200 | 34.9703 | 13.5048 |
| 2012/3/14 | I206 | 86.499 | 5.999 | 3947 | 5 | 33.3549 | 28.9992 |
| 2012/3/14 | I206 | 86.499 | 5.999 | 3947 | 25 | 34.138 | 28.5309 |
| 2012/3/14 | I206 | 86.499 | 5.999 | 3947 | 75 | 34.9536 | 27.0049 |
| 2012/3/14 | I206 | 86.499 | 5.999 | 3947 | 100 | 34.7291 | 21.108 |
| 2012/3/14 | I206 | 86.499 | 5.999 | 3947 | 150 | 34.9273 | 15.2032 |
| 2012/3/14 | I206 | 86.499 | 5.999 | 3947 | 200 | 34.9828 | 13.3345 |
| 2012/3/14 | I207 | 86 | 5.999 | 3933 | 5 | 33.6423 | 29.3206 |
| 2012/3/14 | I207 | 86 | 5.999 | 3933 | 25 | 34.0431 | 28.9402 |
| 2012/3/14 | I207 | 86 | 5.999 | 3933 | 50 | 34.5283 | 27.9528 |
| 2012/3/14 | I207 | 86 | 5.999 | 3933 | 75 | 35.1371 | 27.7961 |
| 2012/3/14 | I207 | 86 | 5.999 | 3933 | 100 | 34.8732 | 21.4704 |
| 2012/3/14 | I207 | 86 | 5.999 | 3933 | 150 | 34.9064 | 16.0681 |
| 2012/3/14 | I207 | 86 | 5.999 | 3933 | 200 | 34.997 | 13.4039 |
| 2012/3/14 | I207 | 86 | 5.999 | 3933 | 5 | 33.6423 | 29.3206 |
| 2012/3/14 | I207 | 86 | 5.999 | 3933 | 11 | 33.6511 | 29.1905 |
| 2012/3/14 | I207 | 86 | 5.999 | 3933 | 25 | 34.051 | 28.9588 |
| 2012/3/14 | I207 | 86 | 5.999 | 3933 | 35 | 34.5074 | 28.1341 |
| 2012/3/14 | I207 | 86 | 5.999 | 3933 | 75 | 35.024 | 27.0317 |
| 2012/3/14 | I208 | 85.497 | 5.997 | 3928 | 5 | 33.8643 | 29.297 |
| 2012/3/14 | I208 | 85.497 | 5.997 | 3928 | 25 | 33.8611 | 29.2982 |
| 2012/3/14 | I208 | 85.497 | 5.997 | 3928 | 50 | 34.5532 | 29.199 |
| 2012/3/14 | I208 | 85.497 | 5.997 | 3928 | 75 | 34.7307 | 27.0455 |
| 2012/3/14 | I208 | 85.497 | 5.997 | 3928 | 100 | 34.9917 | 21.3603 |
| 2012/3/14 | I208 | 85.497 | 5.997 | 3928 | 150 | 34.8658 | 16.3584 |
| 2012/3/14 | I208 | 85.497 | 5.997 | 3928 | 200 | 35.0022 | 13.7134 |
| 2012/3/14 | I208 | 85.497 | 5.997 | 3928 | 0 |  |  |
| 2012/3/15 | I209 | 84.975 | 5.999 | 3946 | 5 | 33.65 | 29.1748 |
| 2012/3/15 | I209 | 84.975 | 5.999 | 3946 | 25 | 33.6933 | 29.1996 |
| 2012/3/15 | I209 | 84.975 | 5.999 | 3946 | 50 | 34.4304 | 27.7713 |
| 2012/3/15 | I209 | 84.975 | 5.999 | 3946 | 75 | 34.7412 | 25.5148 |
| 2012/3/15 | I209 | 84.975 | 5.999 | 3946 | 100 | 34.8098 | 22.9183 |
| 2012/3/15 | I209 | 84.975 | 5.999 | 3946 | 150 | 34.9124 | 17.7264 |
| 2012/3/15 | I209 | 84.975 | 5.999 | 3946 | 200 | 34.9699 | 13.6809 |
| 2012/3/15 | I210 | 84.495 | 5.997 | 3950 | 5 | 33.6784 | 29.3835 |
| 2012/3/15 | I210 | 84.495 | 5.997 | 3950 | 25 | 33.846 | 29.2076 |
| 2012/3/15 | I210 | 84.495 | 5.997 | 3950 | 75 | 35.0859 | 26.4566 |
| 2012/3/15 | I210 | 84.495 | 5.997 | 3950 | 100 | 34.8558 | 22.3706 |
| 2012/3/15 | I210 | 84.495 | 5.997 | 3950 | 150 | 34.9581 | 18.3267 |
| 2012/3/15 | I210 | 84.495 | 5.997 | 3950 | 200 | 34.9582 | 13.9708 |
| 2012/3/16 | I211 | 83.995 | 5.996 | 3993 | 5 | 33.8269 | 29.2213 |
| 2012/3/16 | I211 | 83.995 | 5.996 | 3993 | 25 | 33.8453 | 29.1755 |
| 2012/3/16 | I211 | 83.995 | 5.996 | 3993 | 50 | 34.4996 | 27.5717 |
| 2012/3/16 | I211 | 83.995 | 5.996 | 3993 | 75 | 34.7631 | 26.1212 |
| 2012/3/16 | I211 | 83.995 | 5.996 | 3993 | 100 | 34.7897 | 21.5099 |
| 2012/3/16 | I211 | 83.995 | 5.996 | 3993 | 150 | 34.836 | 17.5496 |
| 2012/3/16 | I211 | 83.995 | 5.996 | 3993 | 200 | 34.9662 | 13.5244 |
| 2012/3/16 | I212 | 83.505 | 5.996 | 4028 | 5 | 33.8861 | 29.2628 |
| 2012/3/16 | I212 | 83.505 | 5.996 | 4028 | 25 | 34.2198 | 29.1859 |
| 2012/3/16 | I212 | 83.505 | 5.996 | 4028 | 50 | 34.2343 | 27.5591 |
| 2012/3/16 | I212 | 83.505 | 5.996 | 4028 | 75 | 34.7647 | 26.6004 |
| 2012/3/16 | I212 | 83.505 | 5.996 | 4028 | 100 | 34.818 | 22.2558 |
| 2012/3/16 | I212 | 83.505 | 5.996 | 4028 | 150 | 34.8148 | 18.2078 |
| 2012/3/16 | I212 | 83.505 | 5.996 | 4028 | 200 | 34.9466 | 14.4106 |
| 2012/3/16 | I213 | 82.997 | 5.997 | 4029 | 5 | 33.7035 | 29.401 |
| 2012/3/16 | I213 | 82.997 | 5.997 | 4029 | 25 | 33.9291 | 29.2897 |
| 2012/3/16 | I213 | 82.997 | 5.997 | 4029 | 50 | 34.6368 | 28.7744 |
| 2012/3/16 | I213 | 82.997 | 5.997 | 4029 | 75 | 34.5265 | 24.2317 |
| 2012/3/16 | I213 | 82.997 | 5.997 | 4029 | 100 | 34.9731 | 21.3292 |
| 2012/3/16 | I213 | 82.997 | 5.997 | 4029 | 150 | 34.8623 | 17.31 |
| 2012/3/16 | I213 | 82.997 | 5.997 | 4029 | 200 | 35.0057 | 14.3981 |
| 2012/3/16 | I213 | 82.997 | 5.997 | 4029 | 0 |  |  |
| 2012/3/16 | I214 | 82.498 | 6.001 | 4039 | 25 | 33.7348 | 29.2406 |
| 2012/3/16 | I214 | 82.498 | 6.001 | 4039 | 50 | 34.6659 | 28.1589 |
| 2012/3/16 | I214 | 82.498 | 6.001 | 4039 | 75 | 35.353 | 24.59 |
| 2012/3/16 | I214 | 82.498 | 6.001 | 4039 | 100 | 35.3311 | 23.0449 |
| 2012/3/16 | I214 | 82.498 | 6.001 | 4039 | 150 | 34.8883 | 17.0337 |
| 2012/3/16 | I214 | 82.498 | 6.001 | 4039 | 200 | 34.9874 | 13.9147 |
| 2012/3/17 | I712 | 82.503 | 4.005 | 4192 | 0 |  |  |
| 2012/3/17 | I712 | 82.503 | 4.005 | 4192 | 25 | 34.4964 | 29.1547 |
| 2012/3/17 | I712 | 82.503 | 4.005 | 4192 | 50 | 35.1286 | 28.6919 |
| 2012/3/17 | I712 | 82.503 | 4.005 | 4192 | 75 | 34.7549 | 21.6454 |
| 2012/3/17 | I712 | 82.503 | 4.005 | 4192 | 100 | 35.0739 | 19.6248 |
| 2012/3/17 | I712 | 82.503 | 4.005 | 4192 | 150 | 35.1067 | 15.6763 |
| 2012/3/17 | I712 | 82.503 | 4.005 | 4192 | 200 | 35.1105 | 13.777 |
| 2012/3/18 | I713 | 82.507 | 3.002 | 2657 | 5 | 34.6184 | 29.5827 |
| 2012/3/18 | I713 | 82.507 | 3.002 | 2657 | 25 | 34.8041 | 29.233 |
| 2012/3/18 | I713 | 82.507 | 3.002 | 2657 | 50 | 35.111 | 28.5076 |
| 2012/3/18 | I713 | 82.507 | 3.002 | 2657 | 75 | 35.313 | 23.7739 |
| 2012/3/18 | I713 | 82.507 | 3.002 | 2657 | 100 | 35.3102 | 21.6362 |
| 2012/3/18 | I713 | 82.507 | 3.002 | 2657 | 150 | 35.0279 | 14.7526 |
| 2012/3/18 | I713 | 82.507 | 3.002 | 2657 | 200 | 35.0474 | 13.2062 |
| 2012/3/18 | I714 | 82.503 | 2.001 | 4373 | 0 |  |  |
| 2012/3/18 | I714 | 82.503 | 2.001 | 4373 | 25 | 34.3176 | 29.3127 |
| 2012/3/18 | I714 | 82.503 | 2.001 | 4373 | 50 | 34.9326 | 27.5919 |
| 2012/3/18 | I714 | 82.503 | 2.001 | 4373 | 75 | 35.3012 | 25.0527 |
| 2012/3/18 | I714 | 82.503 | 2.001 | 4373 | 100 | 35.3043 | 22.1613 |
| 2012/3/18 | I714 | 82.503 | 2.001 | 4373 | 150 | 34.9457 | 16.638 |
| 2012/3/18 | I714 | 82.503 | 2.001 | 4373 | 200 | 35.0274 | 13.5523 |
| 2012/3/18 | I715 | 82.506 | 1.004 | 4464 | 5 | 34.264 | 29.649 |
| 2012/3/18 | I715 | 82.506 | 1.004 | 4464 | 25 | 34.2595 | 29.3523 |
| 2012/3/18 | I715 | 82.506 | 1.004 | 4464 | 50 | 34.323 | 29.1822 |
| 2012/3/18 | I715 | 82.506 | 1.004 | 4464 | 75 | 35.0589 | 25.9277 |
| 2012/3/18 | I715 | 82.506 | 1.004 | 4464 | 100 | 35.1212 | 22.964 |
| 2012/3/18 | I715 | 82.506 | 1.004 | 4464 | 150 | 35.1002 | 17.5184 |
| 2012/3/18 | I715 | 82.506 | 1.004 | 4464 | 200 | 35.1298 | 13.888 |
| 2012/3/19 | I701 | 82.499 | -0.499 | 4476 | 0 |  |  |
| 2012/3/19 | I701 | 82.499 | -0.499 | 4476 | 10 | 34.2526 | 29.0824 |
| 2012/3/19 | I701 | 82.499 | -0.499 | 4476 | 25 | 34.246 | 28.9873 |
| 2012/3/19 | I701 | 82.499 | -0.499 | 4476 | 40 | 34.2519 | 28.9521 |
| 2012/3/19 | I701 | 82.499 | -0.499 | 4476 | 75 | 35.2623 | 22.7823 |
| 2012/3/19 | I701 | 82.499 | -0.499 | 4476 | 100 | 35.2607 | 20.6868 |
| 2012/3/19 | I701 | 82.499 | -0.499 | 4476 | 150 | 35.1461 | 15.7441 |
| 2012/3/19 | I701 | 82.499 | -0.499 | 4476 | 200 | 35.1128 | 13.6343 |
| 2012/3/19 | I703 | 82.502 | -1.494 | 4700 | 0 |  |  |
| 2012/3/19 | I703 | 82.502 | -1.494 | 4700 | 25 | 34.2612 | 28.6783 |
| 2012/3/19 | I703 | 82.502 | -1.494 | 4700 | 50 | 34.4408 | 28.7978 |
| 2012/3/19 | I703 | 82.502 | -1.494 | 4700 | 75 | 35.3637 | 25.761 |
| 2012/3/19 | I703 | 82.502 | -1.494 | 4700 | 100 | 35.2662 | 21.2515 |
| 2012/3/19 | I703 | 82.502 | -1.494 | 4700 | 150 | 35.1363 | 16.2229 |
| 2012/3/19 | I703 | 82.502 | -1.494 | 4700 | 200 | 35.1104 | 13.4051 |
| 2012/3/19 | I703 | 82.502 | -1.494 | 4700 | 0 |  |  |
| 2012/3/20 | I705 | 82.5 | -2.498 | 4620 | 5 | 34.0059 | 28.6238 |
| 2012/3/20 | I705 | 82.5 | -2.498 | 4620 | 25 | 34.0773 | 28.4794 |
| 2012/3/20 | I705 | 82.5 | -2.498 | 4620 | 50 | 35.3673 | 26.5524 |
| 2012/3/20 | I705 | 82.5 | -2.498 | 4620 | 75 | 35.3288 | 23.1014 |
| 2012/3/20 | I705 | 82.5 | -2.498 | 4620 | 100 | 35.2845 | 21.056 |
| 2012/3/20 | I705 | 82.5 | -2.498 | 4620 | 150 | 35.1406 | 15.6846 |
| 2012/3/20 | I705 | 82.5 | -2.498 | 4620 | 200 | 35.0695 | 13.0584 |
| 2012/3/20 | I707 | 82.502 | -3.499 | 4071 | 5 | 33.8813 | 28.5901 |
| 2012/3/20 | I707 | 82.502 | -3.499 | 4071 | 25 | 33.8923 | 28.3853 |
| 2012/3/20 | I707 | 82.502 | -3.499 | 4071 | 50 | 35.121 | 25.4241 |
| 2012/3/20 | I707 | 82.502 | -3.499 | 4071 | 75 | 35.176 | 20.9623 |
| 2012/3/20 | I707 | 82.502 | -3.499 | 4071 | 100 | 35.1984 | 18.2435 |
| 2012/3/20 | I707 | 82.502 | -3.499 | 4071 | 150 | 35.048 | 15.0022 |
| 2012/3/20 | I707 | 82.502 | -3.499 | 4071 | 200 | 35.0283 | 12.9084 |
| 2012/3/20 | I707 | 82.502 | -3.499 | 4071 | 0 |  |  |
| 2012/3/21 | I709 | 82.505 | -4.491 | 3788 | 5 | 34.0684 | 28.0409 |
| 2012/3/21 | I709 | 82.505 | -4.491 | 3788 | 25 | 34.0643 | 28.0381 |
| 2012/3/21 | I709 | 82.505 | -4.491 | 3788 | 50 | 34.2218 | 26.7754 |
| 2012/3/21 | I709 | 82.505 | -4.491 | 3788 | 75 | 34.8107 | 19.48 |
| 2012/3/21 | I709 | 82.505 | -4.491 | 3788 | 100 | 35.1001 | 17.2698 |
| 2012/3/21 | I709 | 82.505 | -4.491 | 3788 | 150 | 34.989 | 13.9624 |
| 2012/3/21 | I709 | 82.505 | -4.491 | 3788 | 200 | 35.044 | 12.5418 |
| 2012/3/21 | I322 | 82.001 | -4.997 | 5112 | 5 | 34.075 | 28.1868 |
| 2012/3/21 | I322 | 82.001 | -4.997 | 5112 | 25 | 34.0691 | 28.1687 |
| 2012/3/21 | I322 | 82.001 | -4.997 | 5112 | 50 | 34.2573 | 26.3327 |
| 2012/3/21 | I322 | 82.001 | -4.997 | 5112 | 75 | 35.0121 | 19.3489 |
| 2012/3/21 | I322 | 82.001 | -4.997 | 5112 | 100 | 35.0678 | 16.9911 |
| 2012/3/21 | I322 | 82.001 | -4.997 | 5112 | 150 | 34.9483 | 13.5021 |
| 2012/3/21 | I322 | 82.001 | -4.997 | 5112 | 200 | 35.0148 | 12.2231 |
| 2012/3/21 | I322 | 82.001 | -4.997 | 5112 | 0 |  |  |
| 2012/3/22 | I321 | 81.003 | -5 | 5102 | 10 | 33.9653 | 28.1757 |
| 2012/3/22 | I321 | 81.003 | -5 | 5102 | 25 | 33.9622 | 28.1812 |
| 2012/3/22 | I321 | 81.003 | -5 | 5102 | 40 | 34.017 | 27.7013 |
| 2012/3/22 | I321 | 81.003 | -5 | 5102 | 75 | 34.9572 | 19.8533 |
| 2012/3/22 | I321 | 81.003 | -5 | 5102 | 100 | 34.9792 | 16.283 |
| 2012/3/22 | I321 | 81.003 | -5 | 5102 | 150 | 34.882 | 12.9196 |
| 2012/3/22 | I321 | 81.003 | -5 | 5102 | 200 | 34.9722 | 11.9897 |
| 2012/3/22 | I320 | 80.004 | -4.991 | 5118 | 0 |  |  |
| 2012/3/22 | I320 | 80.004 | -4.991 | 5118 | 25 | 33.8955 | 28.2956 |
| 2012/3/22 | I320 | 80.004 | -4.991 | 5118 | 50 | 34.9833 | 24.303 |
| 2012/3/22 | I320 | 80.004 | -4.991 | 5118 | 75 | 34.9793 | 19.0245 |
| 2012/3/22 | I320 | 80.004 | -4.991 | 5118 | 100 | 34.9217 | 17.2171 |
| 2012/3/22 | I320 | 80.004 | -4.991 | 5118 | 150 | 34.8649 | 13.6622 |
| 2012/3/22 | I320 | 80.004 | -4.991 | 5118 | 200 | 34.89 | 12.3202 |
| 2012/3/23 | I318 | 80.002 | -3.986 | 4603 | 5 | 33.987 | 28.5362 |
| 2012/3/23 | I318 | 80.002 | -3.986 | 4603 | 25 | 33.9832 | 28.4391 |
| 2012/3/23 | I318 | 80.002 | -3.986 | 4603 | 50 | 34.9447 | 27.5964 |
| 2012/3/23 | I318 | 80.002 | -3.986 | 4603 | 75 | 35.2449 | 21.6522 |
| 2012/3/23 | I318 | 80.002 | -3.986 | 4603 | 100 | 35.2236 | 19.2209 |
| 2012/3/23 | I318 | 80.002 | -3.986 | 4603 | 150 | 35.0597 | 15.1204 |
| 2012/3/23 | I318 | 80.002 | -3.986 | 4603 | 200 | 34.9643 | 12.5765 |
| 2012/3/23 | I318 | 80.002 | -3.986 | 4603 | 0 |  |  |
| 2012/3/23 | I316 | 80.013 | -2.996 | 4937 | 25 |  |  |
| 2012/3/23 | I316 | 80.013 | -2.996 | 4937 | 50 |  |  |
| 2012/3/23 | I316 | 80.013 | -2.996 | 4937 | 75 |  |  |
| 2012/3/23 | I316 | 80.013 | -2.996 | 4937 | 100 | 35.3202 | 23.8381 |
| 2012/3/23 | I316 | 80.013 | -2.996 | 4937 | 150 | 35.1535 | 17.475 |
| 2012/3/23 | I316 | 80.013 | -2.996 | 4937 | 200 | 35.0365 | 13.2994 |
| 2012/3/24 | I314 | 80.005 | -1.996 | 4883 | 5 | 34.4113 | 28.9108 |
| 2012/3/24 | I314 | 80.005 | -1.996 | 4883 | 10 | 34.4114 | 28.9091 |
| 2012/3/24 | I314 | 80.005 | -1.996 | 4883 | 25 | 34.4104 | 28.9125 |
| 2012/3/24 | I314 | 80.005 | -1.996 | 4883 | 40 | 34.4159 | 28.9212 |
| 2012/3/24 | I314 | 80.005 | -1.996 | 4883 | 75 | 35.1047 | 23.6771 |
| 2012/3/24 | I314 | 80.005 | -1.996 | 4883 | 100 | 35.2227 | 21.559 |
| 2012/3/24 | I314 | 80.005 | -1.996 | 4883 | 150 | 35.1694 | 17.8538 |
| 2012/3/24 | I314 | 80.005 | -1.996 | 4883 | 200 | 35.0784 | 13.6085 |
| 2012/3/24 | I312 | 80 | -0.994 | 4709 | 5 | 34.5695 | 29.1859 |
| 2012/3/24 | I312 | 80 | -0.994 | 4709 | 25 | 34.5667 | 29.0602 |
| 2012/3/24 | I312 | 80 | -0.994 | 4709 | 50 | 34.5578 | 28.9098 |
| 2012/3/24 | I312 | 80 | -0.994 | 4709 | 75 | 34.9757 | 25.0408 |
| 2012/3/24 | I312 | 80 | -0.994 | 4709 | 100 | 35.2797 | 22.2472 |
| 2012/3/24 | I312 | 80 | -0.994 | 4709 | 150 | 35.1688 | 16.78 |
| 2012/3/24 | I312 | 80 | -0.994 | 4709 | 200 | 35.1156 | 13.658 |
| 2012/3/25 | I401 | 80.003 | 0.002 | 4656 | 0 |  |  |
| 2012/3/25 | I401 | 80.003 | 0.002 | 4656 | 25 | 34.4155 | 29.4824 |
| 2012/3/25 | I401 | 80.003 | 0.002 | 4656 | 50 | 34.4843 | 29.0221 |
| 2012/3/25 | I401 | 80.003 | 0.002 | 4656 | 75 | 34.9128 | 26.3858 |
| 2012/3/25 | I401 | 80.003 | 0.002 | 4656 | 100 | 35.2708 | 22.6629 |
| 2012/3/25 | I401 | 80.003 | 0.002 | 4656 | 150 | 35.1275 | 15.6375 |
| 2012/3/25 | I401 | 80.003 | 0.002 | 4656 | 200 | 35.1023 | 13.1703 |
| 2012/3/26 | I309 | 80.001 | 0.999 | 4610 | 5 | 34.5179 | 29.6982 |
| 2012/3/26 | I309 | 80.001 | 0.999 | 4610 | 25 | 34.5083 | 29.6188 |
| 2012/3/26 | I309 | 80.001 | 0.999 | 4610 | 50 | 34.5995 | 29.2142 |
| 2012/3/26 | I309 | 80.001 | 0.999 | 4610 | 75 | 34.7593 | 28.1274 |
| 2012/3/26 | I309 | 80.001 | 0.999 | 4610 | 100 | 35.2398 | 21.0053 |
| 2012/3/26 | I309 | 80.001 | 0.999 | 4610 | 150 | 35.0966 | 15.1605 |
| 2012/3/26 | I309 | 80.001 | 0.999 | 4610 | 200 | 35.0966 | 13.0815 |
| 2012/3/26 | I307 | 80.006 | 1.998 | 3903 | 0 |  |  |
| 2012/3/26 | I307 | 80.006 | 1.998 | 3903 | 25 | 34.7526 | 29.6451 |
| 2012/3/26 | I307 | 80.006 | 1.998 | 3903 | 50 | 34.8919 | 29.1445 |
| 2012/3/26 | I307 | 80.006 | 1.998 | 3903 | 75 | 35.0501 | 26.6326 |
| 2012/3/26 | I307 | 80.006 | 1.998 | 3903 | 100 | 35.0893 | 23.0119 |
| 2012/3/26 | I307 | 80.006 | 1.998 | 3903 | 150 | 35.0479 | 15.2796 |
| 2012/3/26 | I307 | 80.006 | 1.998 | 3903 | 200 | 35.1086 | 13.5632 |
| 2012/4/1 | I301 | 80 | 4.999 | 4211 | 5 | 34.2787 | 30.0177 |
| 2012/4/1 | I301 | 80 | 4.999 | 4211 | 25 | 34.2843 | 29.9634 |
| 2012/4/1 | I301 | 80 | 4.999 | 4211 | 50 | 35.1183 | 28.8617 |
| 2012/4/1 | I301 | 80 | 4.999 | 4211 | 75 | 34.7322 | 22.4564 |
| 2012/4/1 | I301 | 80 | 4.999 | 4211 | 100 | 35.1397 | 20.2415 |
| 2012/4/1 | I301 | 80 | 4.999 | 4211 | 150 | 35.0003 | 16.4917 |
| 2012/4/1 | I301 | 80 | 4.999 | 4211 | 200 | 35.0641 | 14.2422 |
| 2012/4/1 | I301 | 80 | 4.999 | 4211 | 0 |  |  |
| 2012/4/1 | I303 | 80 | 4.004 | 4325 | 5 | 34.1495 | 30.025 |
| 2012/4/1 | I303 | 80 | 4.004 | 4325 | 25 | 35.0166 | 29.2191 |
| 2012/4/1 | I303 | 80 | 4.004 | 4325 | 50 | 35.2376 | 28.443 |
| 2012/4/1 | I303 | 80 | 4.004 | 4325 | 75 | 35.1523 | 23.0254 |
| 2012/4/1 | I303 | 80 | 4.004 | 4325 | 100 | 35.1789 | 20.5937 |
| 2012/4/1 | I303 | 80 | 4.004 | 4325 | 150 | 35.0473 | 15.8646 |
| 2012/4/1 | I303 | 80 | 4.004 | 4325 | 200 | 35.0914 | 13.8595 |
| 2012/4/2 | I305 | 80.002 | 3.002 | 2657 | 5 | 34.6347 | 29.8728 |
| 2012/4/2 | I305 | 80.002 | 3.002 | 2657 | 10 | 34.6529 | 29.7609 |
| 2012/4/2 | I305 | 80.002 | 3.002 | 2657 | 25 | 34.6642 | 29.6677 |
| 2012/4/2 | I305 | 80.002 | 3.002 | 2657 | 40 | 34.9714 | 29.2995 |
| 2012/4/2 | I305 | 80.002 | 3.002 | 2657 | 75 | 35.1189 | 24.4026 |
| 2012/4/2 | I305 | 80.002 | 3.002 | 2657 | 100 | 35.0915 | 20.9242 |
| 2012/4/2 | I305 | 80.002 | 3.002 | 2657 | 150 | 35.0362 | 16.5963 |
| 2012/4/2 | I305 | 80.002 | 3.002 | 2657 | 200 | 35.0963 | 13.6707 |
| 2012/4/3 | I402 | 80.999 | 0.002 | 4591 | 5 | 34.4559 | 29.8332 |
| 2012/4/3 | I402 | 80.999 | 0.002 | 4591 | 25 | 34.4974 | 29.8425 |
| 2012/4/3 | I402 | 80.999 | 0.002 | 4591 | 50 | 34.5691 | 29.6371 |
| 2012/4/3 | I402 | 80.999 | 0.002 | 4591 | 75 | 34.9263 | 25.7159 |
| 2012/4/3 | I402 | 80.999 | 0.002 | 4591 | 100 | 35.2867 | 20.7882 |
| 2012/4/3 | I402 | 80.999 | 0.002 | 4591 | 150 | 35.1369 | 16.7918 |
| 2012/4/3 | I402 | 80.999 | 0.002 | 4591 | 200 | 35.1023 | 13.2676 |
| 2012/4/3 | I403 | 80 | 0 | 4576 | 0 |  |  |
| 2012/4/3 | I403 | 80 | 0 | 4576 | 25 | 34.4897 | 29.8277 |
| 2012/4/3 | I403 | 80 | 0 | 4576 | 50 | 34.5217 | 29.4755 |
| 2012/4/3 | I403 | 80 | 0 | 4576 | 75 | 34.632 | 28.9472 |
| 2012/4/3 | I403 | 80 | 0 | 4576 | 100 | 35.2895 | 22.0969 |
| 2012/4/3 | I403 | 80 | 0 | 4576 | 150 | 35.1711 | 18.4954 |
| 2012/4/3 | I403 | 80 | 0 | 4576 | 200 | 35.1057 | 13.3181 |
| 2012/4/4 | I404 | 82.996 | 0 | 4365 | 5 | 34.3766 | 29.7305 |
| 2012/4/4 | I404 | 82.996 | 0 | 4365 | 25 | 34.3498 | 29.5917 |
| 2012/4/4 | I404 | 82.996 | 0 | 4365 | 50 | 34.3009 | 29.3141 |
| 2012/4/4 | I404 | 82.996 | 0 | 4365 | 75 | 34.4629 | 28.3705 |
| 2012/4/4 | I404 | 82.996 | 0 | 4365 | 100 | 35.3552 | 21.6292 |
| 2012/4/4 | I404 | 82.996 | 0 | 4365 | 150 | 35.1789 | 17.0481 |
| 2012/4/4 | I404 | 82.996 | 0 | 4365 | 200 | 35.1051 | 13.3144 |
| 2012/4/4 | I404 | 82.996 | 0 | 4365 | 0 |  |  |
| 2012/4/4 | I405 | 84.002 | 0.003 | 4537 | 0 |  |  |
| 2012/4/4 | I405 | 84.002 | 0.003 | 4537 | 25 | 34.278 | 29.717 |
| 2012/4/4 | I405 | 84.002 | 0.003 | 4537 | 50 | 34.3191 | 29.4517 |
| 2012/4/4 | I405 | 84.002 | 0.003 | 4537 | 75 | 34.7329 | 26.2496 |
| 2012/4/4 | I405 | 84.002 | 0.003 | 4537 | 100 | 35.2827 | 21.4833 |
| 2012/4/4 | I405 | 84.002 | 0.003 | 4537 | 150 | 35.1716 | 16.7235 |
| 2012/4/4 | I405 | 84.002 | 0.003 | 4537 | 200 | 35.0891 | 12.8022 |
| 2012/4/5 | I406 | 84.999 | 0.006 | 4521 | 5 | 34.3994 | 29.7285 |
| 2012/4/5 | I406 | 84.999 | 0.006 | 4521 | 25 | 34.388 | 29.7045 |
| 2012/4/5 | I406 | 84.999 | 0.006 | 4521 | 50 | 34.3156 | 29.3878 |
| 2012/4/5 | I406 | 84.999 | 0.006 | 4521 | 75 | 34.9385 | 24.7675 |
| 2012/4/5 | I406 | 84.999 | 0.006 | 4521 | 100 | 35.261 | 21.7739 |
| 2012/4/5 | I406 | 84.999 | 0.006 | 4521 | 150 | 35.1162 | 16.9613 |
| 2012/4/5 | I406 | 84.999 | 0.006 | 4521 | 200 | 35.0975 | 13.0786 |
| 2012/4/5 | I407 | 86.002 | 0.003 | 4505 | 0 |  |  |
| 2012/4/5 | I407 | 86.002 | 0.003 | 4505 | 25 | 34.1109 | 29.0966 |
| 2012/4/5 | I407 | 86.002 | 0.003 | 4505 | 50 | 34.0947 | 28.9925 |
| 2012/4/5 | I407 | 86.002 | 0.003 | 4505 | 75 | 34.0707 | 28.786 |
| 2012/4/5 | I407 | 86.002 | 0.003 | 4505 | 100 | 35.2684 | 21.9925 |
| 2012/4/5 | I407 | 86.002 | 0.003 | 4505 | 150 | 35.1455 | 17.7421 |
| 2012/4/5 | I407 | 86.002 | 0.003 | 4505 | 200 | 35.1253 | 13.7736 |
| 2012/4/5 | I407 | 86.002 | 0.003 | 4505 | 0 |  |  |
| 2012/4/6 | I408 | 87.005 | -0.002 | 4535 | 5 | 34.1007 | 29.5081 |
| 2012/4/6 | I408 | 87.005 | -0.002 | 4535 | 25 | 34.0966 | 29.3429 |
| 2012/4/6 | I408 | 87.005 | -0.002 | 4535 | 50 | 34.0892 | 29.1615 |
| 2012/4/6 | I408 | 87.005 | -0.002 | 4535 | 75 | 34.2299 | 28.8058 |
| 2012/4/6 | I408 | 87.005 | -0.002 | 4535 | 100 | 35.2562 | 21.7201 |
| 2012/4/6 | I408 | 87.005 | -0.002 | 4535 | 150 | 35.1548 | 17.4298 |
| 2012/4/6 | I408 | 87.005 | -0.002 | 4535 | 200 | 35.1101 | 13.4748 |
| 2012/4/6 | I408 | 87.005 | -0.002 | 4535 | 5 | 34.1007 | 29.5081 |
| 2012/4/6 | I408 | 87.005 | -0.002 | 4535 | 10 | 34.1055 | 29.4815 |
| 2012/4/6 | I408 | 87.005 | -0.002 | 4535 | 25 | 34.0966 | 29.3429 |
| 2012/4/6 | I408 | 87.005 | -0.002 | 4535 | 40 | 34.0913 | 29.2176 |
| 2012/4/6 | I408 | 87.005 | -0.002 | 4535 | 75 | 34.2668 | 28.8328 |
| 2012/4/7 | I409 | 88 | -0.006 | 4498 | 0 |  |  |
| 2012/4/7 | I409 | 88 | -0.006 | 4498 | 25 | 34.187 | 29.5341 |
| 2012/4/7 | I409 | 88 | -0.006 | 4498 | 50 | 34.3027 | 29.4482 |
| 2012/4/7 | I409 | 88 | -0.006 | 4498 | 75 | 34.3705 | 29.2759 |
| 2012/4/7 | I409 | 88 | -0.006 | 4498 | 100 | 35.1501 | 21.5886 |
| 2012/4/7 | I409 | 88 | -0.006 | 4498 | 150 | 35.1025 | 16.8224 |
| 2012/4/7 | I409 | 88 | -0.006 | 4498 | 200 | 35.1249 | 14.1317 |
| 2012/4/7 | I410 | 89.001 | -0.001 | 4386 | 5 | 34.2144 | 29.9901 |
| 2012/4/7 | I410 | 89.001 | -0.001 | 4386 | 25 | 34.2049 | 29.783 |
| 2012/4/7 | I410 | 89.001 | -0.001 | 4386 | 50 | 34.2794 | 29.605 |
| 2012/4/7 | I410 | 89.001 | -0.001 | 4386 | 75 | 34.3457 | 29.385 |
| 2012/4/7 | I410 | 89.001 | -0.001 | 4386 | 100 | 35.1386 | 21.5463 |
| 2012/4/7 | I410 | 89.001 | -0.001 | 4386 | 150 | 35.1229 | 16.7649 |
| 2012/4/7 | I410 | 89.001 | -0.001 | 4386 | 200 | 35.1173 | 13.9907 |
| 2012/4/7 | I410 | 89.001 | -0.001 | 4386 | 0 |  |  |
| 2012/4/7 | I411 | 90.003 | 0 | 4193 | 0 |  |  |
| 2012/4/7 | I411 | 90.003 | 0 | 4193 | 25 | 34.2261 | 29.7307 |
| 2012/4/7 | I411 | 90.003 | 0 | 4193 | 50 | 34.2804 | 29.5434 |
| 2012/4/7 | I411 | 90.003 | 0 | 4193 | 75 | 34.3899 | 29.4261 |
| 2012/4/7 | I411 | 90.003 | 0 | 4193 | 100 | 34.5449 | 26.8659 |
| 2012/4/7 | I411 | 90.003 | 0 | 4193 | 150 | 35.1418 | 16.3194 |
| 2012/4/7 | I411 | 90.003 | 0 | 4193 | 200 | 35.1122 | 13.9001 |
| 2012/4/8 | I412 | 91.008 | 0.004 | 4525 | 5 | 34.1852 | 30.0191 |
| 2012/4/8 | I412 | 91.008 | 0.004 | 4525 | 25 | 34.2358 | 29.8036 |
| 2012/4/8 | I412 | 91.008 | 0.004 | 4525 | 50 | 34.3329 | 29.5203 |
| 2012/4/8 | I412 | 91.008 | 0.004 | 4525 | 75 | 34.4253 | 29.3962 |
| 2012/4/8 | I412 | 91.008 | 0.004 | 4525 | 100 | 35.0797 | 21.7274 |
| 2012/4/8 | I412 | 91.008 | 0.004 | 4525 | 150 | 35.1488 | 16.8632 |
| 2012/4/8 | I412 | 91.008 | 0.004 | 4525 | 200 | 35.1041 | 14.133 |
| 2012/4/8 | I413 | 92.007 | -0.005 | 4513 | 0 |  |  |
| 2012/4/8 | I413 | 92.007 | -0.005 | 4513 | 25 | 34.255 | 29.7831 |
| 2012/4/8 | I413 | 92.007 | -0.005 | 4513 | 50 | 34.3617 | 29.6125 |
| 2012/4/8 | I413 | 92.007 | -0.005 | 4513 | 75 | 34.5087 | 29.5807 |
| 2012/4/8 | I413 | 92.007 | -0.005 | 4513 | 100 | 35.0246 | 21.6574 |
| 2012/4/8 | I413 | 92.007 | -0.005 | 4513 | 150 | 35.1322 | 16.6013 |
| 2012/4/8 | I413 | 92.007 | -0.005 | 4513 | 200 | 35.109 | 14.0478 |
| 2012/4/9 | I414 | 93 | 0 | 4490 | 5 | 34.3276 | 30.1555 |
| 2012/4/9 | I414 | 93 | 0 | 4490 | 25 | 34.3137 | 29.808 |
| 2012/4/9 | I414 | 93 | 0 | 4490 | 50 | 34.3793 | 29.6475 |
| 2012/4/9 | I414 | 93 | 0 | 4490 | 75 | 34.4772 | 29.6241 |
| 2012/4/9 | I414 | 93 | 0 | 4490 | 100 | 35.1946 | 20.4475 |
| 2012/4/9 | I414 | 93 | 0 | 4490 | 150 | 35.1186 | 15.5804 |
| 2012/4/9 | I414 | 93 | 0 | 4490 | 200 | 35.084 | 13.2354 |
| 2012/4/9 | I414 | 93 | 0 | 4490 | 5 | 34.3276 | 30.1555 |
| 2012/4/9 | I414 | 93 | 0 | 4490 | 10 | 34.3191 | 30.0104 |
| 2012/4/9 | I414 | 93 | 0 | 4490 | 25 | 34.3137 | 29.808 |
| 2012/4/9 | I414 | 93 | 0 | 4490 | 40 | 34.3462 | 29.7163 |
| 2012/4/9 | I414 | 93 | 0 | 4490 | 75 | 34.4772 | 29.6241 |
| 2012/4/9 | I415 | 93.993 | 0 | 4469 | 5 | 34.1477 | 30.0765 |
| 2012/4/9 | I415 | 93.993 | 0 | 4469 | 25 | 34.3336 | 30.0213 |
| 2012/4/9 | I415 | 93.993 | 0 | 4469 | 50 | 34.3781 | 29.8557 |
| 2012/4/9 | I415 | 93.993 | 0 | 4469 | 75 | 34.4069 | 29.591 |
| 2012/4/9 | I415 | 93.993 | 0 | 4469 | 100 | 35.1365 | 20.578 |
| 2012/4/9 | I415 | 93.993 | 0 | 4469 | 150 | 35.1245 | 16.3353 |
| 2012/4/9 | I415 | 93.993 | 0 | 4469 | 200 | 35.0804 | 13.0901 |
| 2012/4/9 | I415 | 93.993 | 0 | 4469 | 0 |  |  |

The basic information of latitude, longitude, sample depth, temperature, and salinity in the surveyed area.

**Supplementary Table S2**

| **Species** | Frequency of occurrence (%fi) | Relative  abundance(%P) | Dominance degree(Y) |
| --- | --- | --- | --- |
| Dominant coccoliths | | | |
| *Gephyrocapsa oceanica* | 96.5 | 71.76 | 0.6925 |
| *Emiliania huxleyi* | 64.0 | 8.00 | 0.0512 |
| *Umbilicosphaera sibogae* | 62.5 | 6.26 | 0.0391 |
| *Helicosphaera carteri* | 63.5 | 3.50 | 0.0222 |
| *Helicosphaera hyalina* | 61.5 | 3.02 | 0.0186 |
| Dominant coccospheres | | | |
| *Gephyrocapsa oceanica* | 44.5 | 26.18 | 0.2330 |
| *Florisphaera profunda* | 22.0 | 40.78 | 0.1794 |
| *Emiliania huxleyi* | 31.0 | 6.46 | 0.0400 |
| *Umbellosphaera irregularis* | 15.3 | 11.75 | 0.0358 |
| *Umbilicosphaera sibogae* | 30.0 | 4.05 | 0.0243 |

Living coccolithophores composition in the eastern equatorial Indian Ocean during spring intermonsoon period of 2012.

**Supplementary Table S3**

| **Dominant coccoliths** | Min, Max (Mean)  Units (coccoliths ml^-1^) |
| --- | --- |
| *Gephyrocapsa oceanica* | 0, 154.955 (16.260) |
| *Emiliania huxleyi* | 0, 23.706 (1.814) |
| *Umbilicosphaera sibogae* | 0, 29.04 (1.418) |
| *Helicosphaera carteri* | 0, 7.829 (0.793) |
| *Helicosphaera hyalina* | 0, 10.307 (0.685) |
| **Dominant coccospheres** | Min, Max (Mean)  Units (cells ml^-1^) |
| *Gephyrocapsa oceanica* | 0, 24.805 (2.458) |
| *Florisphaera profunda* | 0, 53.845 (3.828) |
| *Emiliania huxleyi* | 0, 20.167 (0.606) |
| *Umbellosphaera irregularis* | 0, 24.675 (1.103) |
| *Umbilicosphaera sibogae* | 0, 3.609 (0.381) |

Predominant species abundance in the eastern equatorial Indian Ocean during spring intermonsoon period of 2012.

**Supplementary Table S4**

| Group | Average similarity | Dominant species contribution |
| --- | --- | --- |
| Coccospheres | | |
| d | 40.49 | *Gephyrocapsa oceanica* (99.52) |
| b | 53.78 | *Gephyrocapsa oceanica* (40.38); *Emiliania huxleyi* (28.62); *Oolithotus fragilis* (11.63); *Florisphaera profunda*(7.97); *Helicosphaera carteri*(4.18) |
| c | 59.53 | *Umbellosphaera irregularis*(43.67); *Umbilicosphaera sibogae*(27.06); *Gephyrocapsa oceanica* (10.28); *Helicosphaera hyaline* (8.07); *Emiliania huxleyi* (5.36) |
| a | 61.21 | *Florisphaera profunda*(61.89); *Gephyrocapsa oceanica* (22.20);*Algirosphaera robusta* (7.02) |

Dominant coccosphere and their contribution to each group revealed by means of SIMPER analysis. Note: the number in parentheses represented abundance percentage.

**Supplementary Table S5**

| Eigenvectors | | | | | |
| --- | --- | --- | --- | --- | --- |
| Variable | PC1 | PC2 | PC3 | PC4 | PC5 |
| Temperature | -0.423 | 0.468 | 0.019 | 0.302 | -0.34 |
| Salinity | 0.468 | -0.102 | 0.137 | 0.311 | -0.787 |
| Density | 0.459 | -0.455 | 0.084 | -0.016 | 0.163 |
| Chl*a* | 0.42 | 0.488 | 0.089 | 0.241 | 0.305 |
| Micro | 0.307 | 0.413 | -0.284 | -0.755 | -0.251 |
| Nano | 0.202 | 0.348 | 0.682 | -0.007 | 0.199 |
| Pico | 0.282 | 0.186 | -0.648 | 0.429 | 0.206 |

The statistical values by PCA analysis in coccosphere matrix.

**Plates Ⅰ-Ⅴ**

Plate Ⅰ Noëlaerhabdaceae: *Emiliania* & *Gephyrocapsa*

*
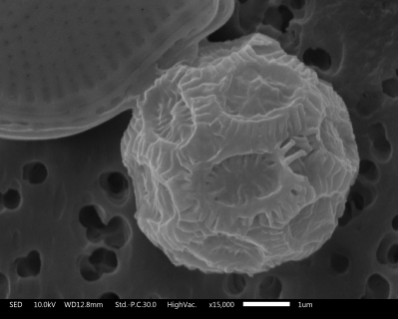
*
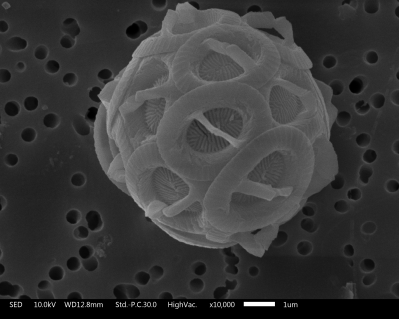

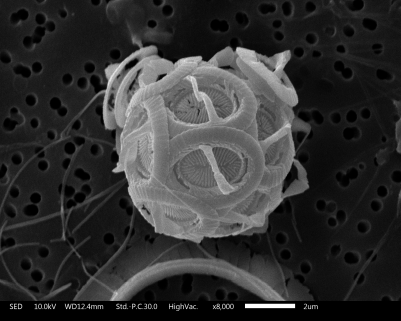


2 μm

1μm

3

2

1

*E. huxleyi* type A overcalcified *G. oceanica*

*
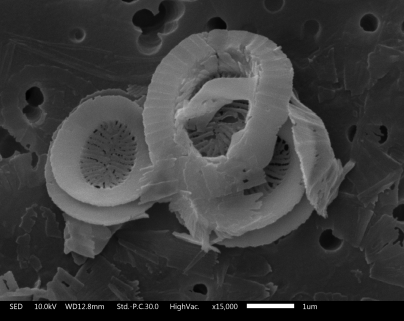

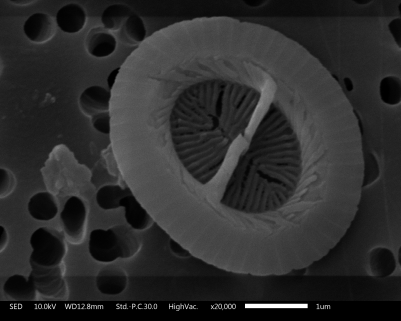
*
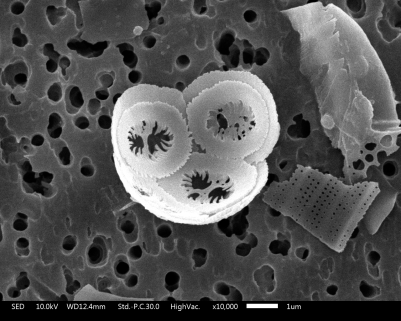


1 μm

1 μm

1 μm

6

5

4

*G. oceanica* coccolith


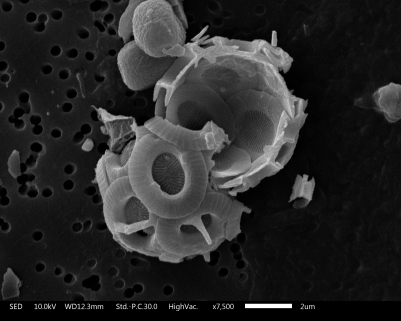

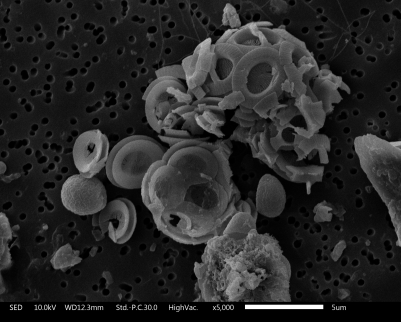

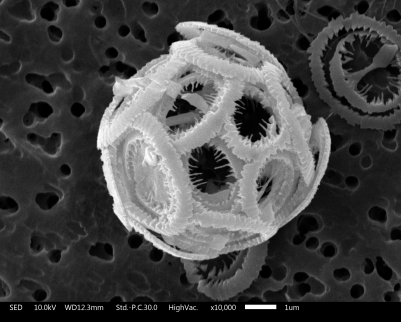


1 μm

9

5 μm

2 μm

8

7

*G. oceanica* collapsed

PlateⅡ. Umbellosphaeraceae: *Umbellosphaera*


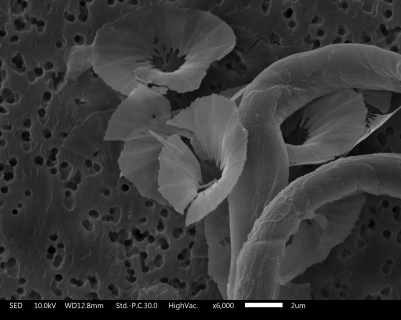
*
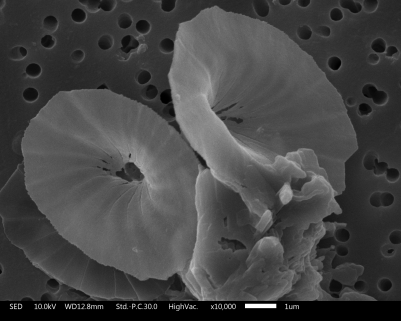

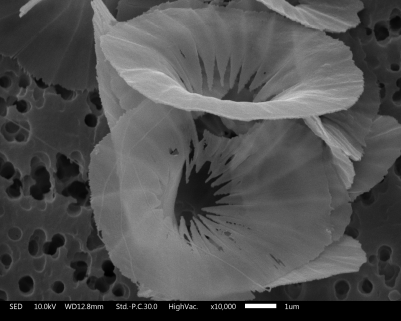
*

3

2

1 μm

2 μm

1

*U. irregularis*

*
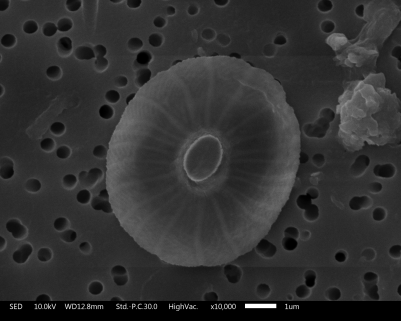

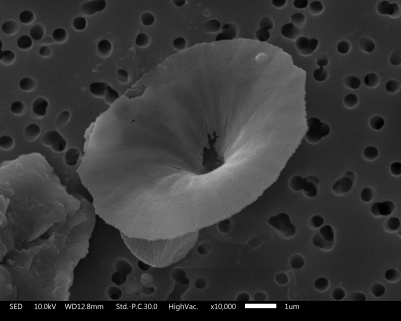

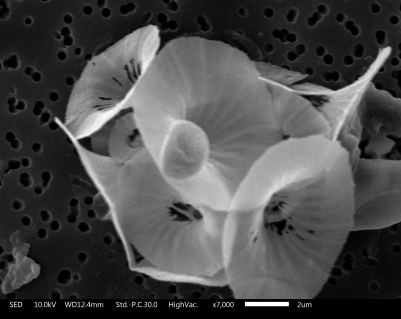
*

2 μm

6

5

1 μm

4

*U. irregularis*


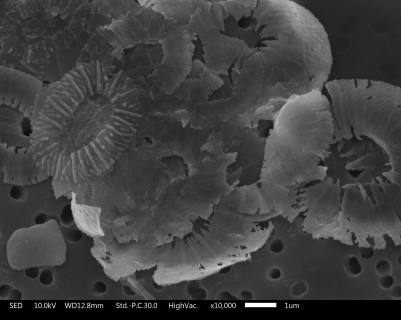

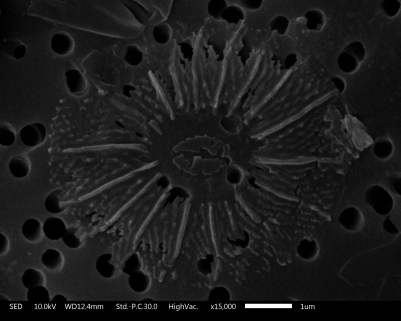

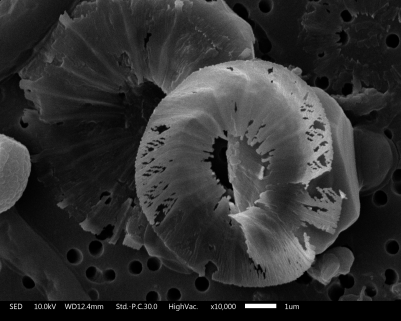


1 μm

1 μm

9

8

1 μm

7

*U. tenuisU. tenuis* type I

Plate Ⅲ Calcidiscaceae: *Umbilicosphaera* & *Calcidiscus*

*
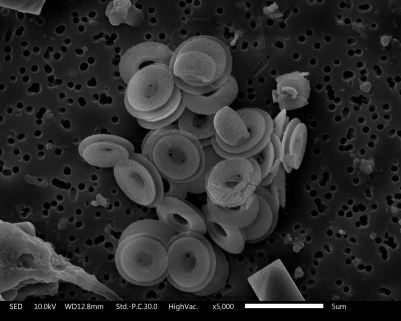

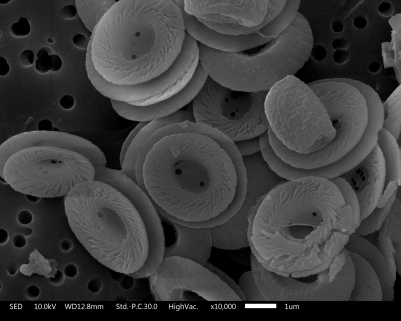

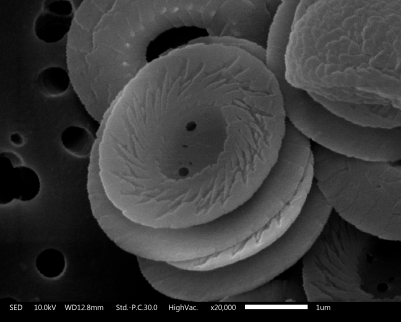
*

1 μm

3

5 μm

1

1 μm

2

*U. hulburtiana*

*
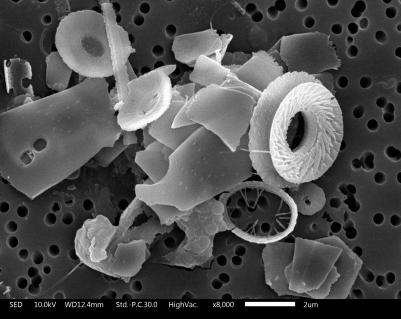
* *
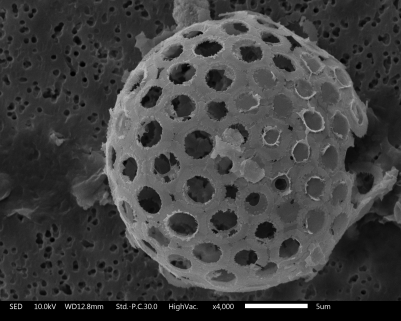
*
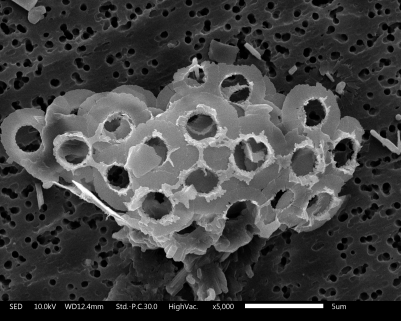


5 μm

2 μm

5 μm

6

4

5

*U. sibogae* coccolith *U. sibogae* cell


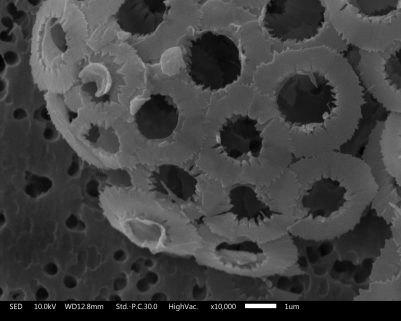

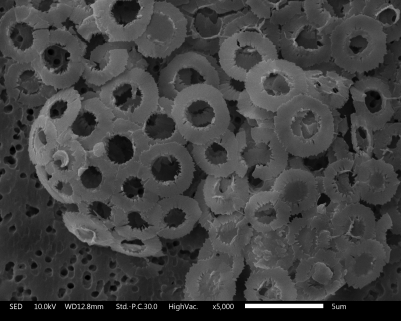

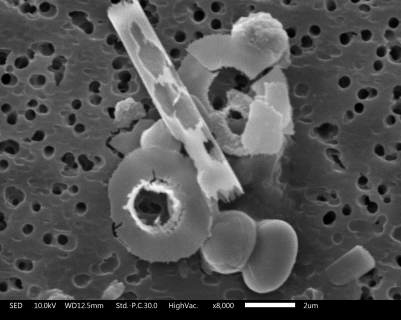


2 μm

9

1 μm

5 μm

8

7

*U. sibogae* cell collapsed *U.* sp. 1 coccolith detached


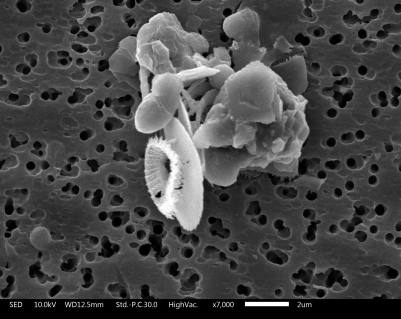

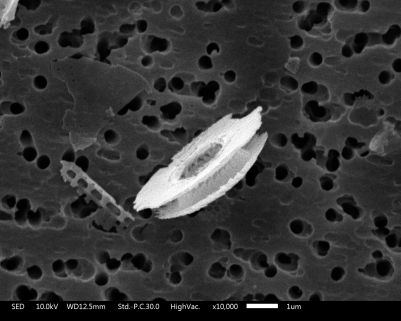

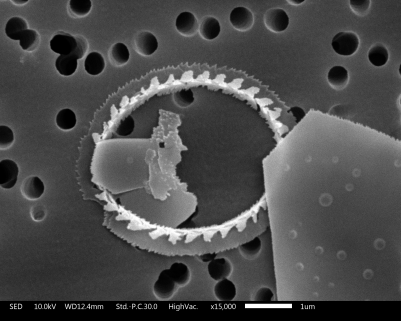


1 μm

1 μm

2 μm

10

12

11

*U.* sp. 2


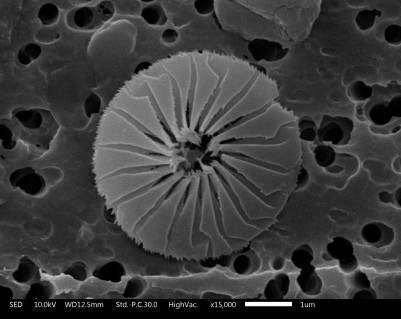


13

15

*C. leptoporus*

Plate Ⅳ *Ceratolithus & Tetraparma & Discosphaera*

*
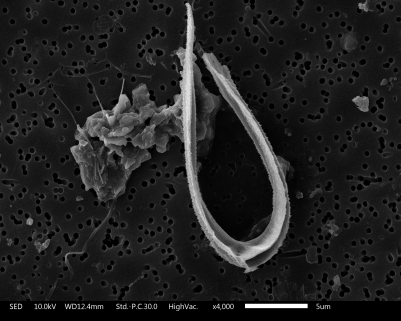

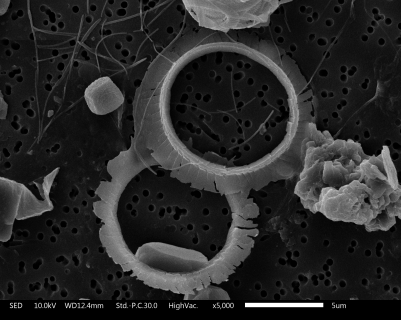

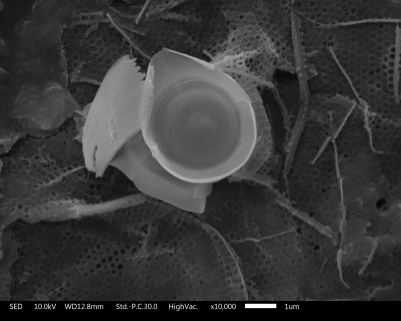
*

3

1 μm

5 μm

2

1

5 μm

*C. cristatus* CER *telesmus* type *C. cristatus* HET coccolithomorpha type *T. trulifera*


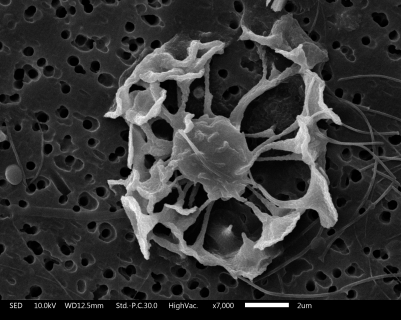


2 μm

4

9

8

*D. tubifera*

Plate Ⅴ. Mixed group

*
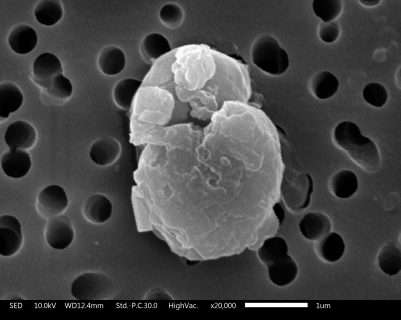

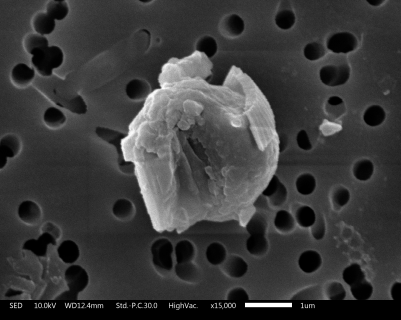

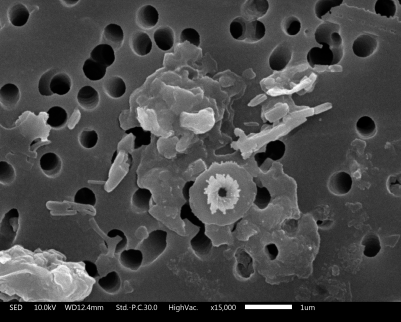
*

1 μm

2 μm

3

2

1

Coccolith-missed coccosphere Cell collapsed


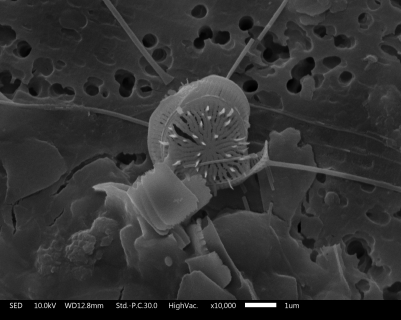
*
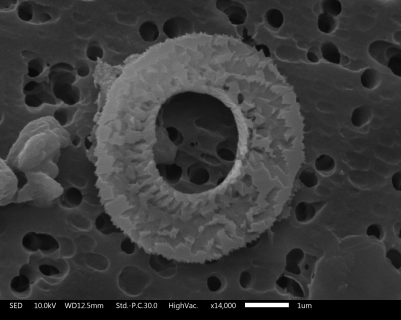
*
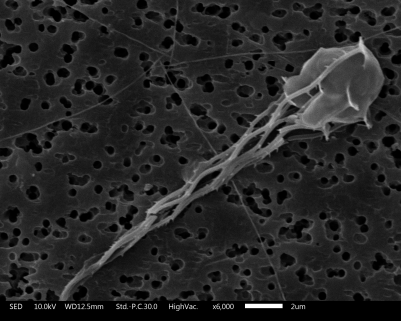


2 μm

1 μm

4

6

5

1 μm

Unknownsp. 1 Unknown sp. 2 Unknown sp. 3


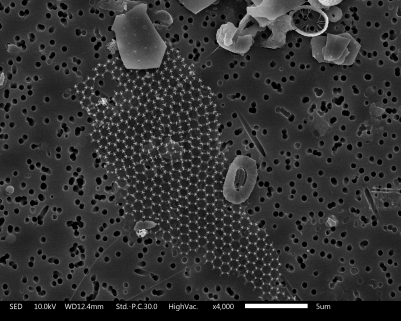

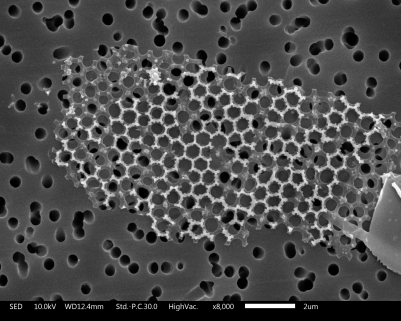

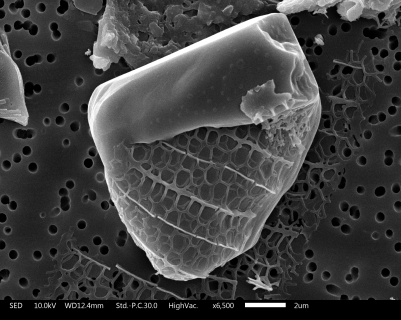


2 μm

8

7

9

2 μm

5 μm

Unknownsp. 3 Unknown sp. 4


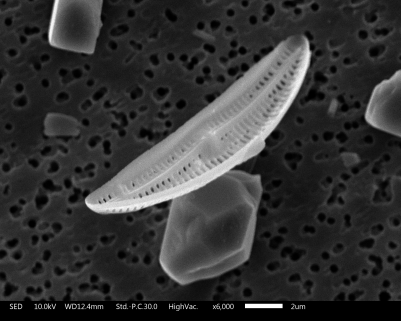
*
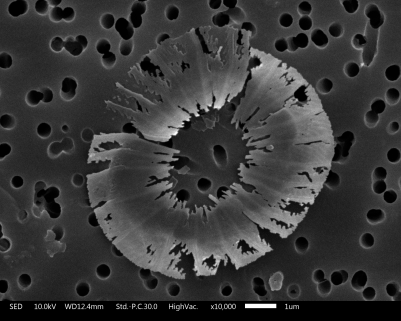
*

1 μm

2 μm

10

11

12

Unknown sp. 4 Coccolith deformed

**Supplementary Table S6**

| Station | Depth | Size fraction | Vol filtration/mL | concentration（μg/L） |
| --- | --- | --- | --- | --- |
| I104A | 0 | m | 800 | 0.013 |
| I104A | 0 | n | 800 | 0.038 |
| I104A | 0 | p | 800 | 0.104 |
| I104A | 25 | m | 800 | 0.005 |
| I104A | 25 | n | 800 | 0.053 |
| I104A | 25 | p | 800 | 0.160 |
| I104A | 75 | m | 800 | 0.011 |
| I104A | 75 | n | 800 | 0.068 |
| I104A | 75 | p | 800 | 0.255 |
| I104A | 100 | m | 500 | 0.003 |
| I104A | 100 | n | 500 | 0.059 |
| I104A | 100 | p | 500 | 0.035 |
| I104A | 150 | m | 800 | 0.002 |
| I104A | 150 | n | 800 | 0.014 |
| I104A | 150 | p | 800 | 0.009 |
| I104A | 200 | m | 800 | 0.002 |
| I104A | 200 | n | 800 | 0.007 |
| I104A | 200 | p | 800 | 0.002 |
| I105A | 5 | total | 800 | 0.130 |
| I105A | 25 | total | 800 | 0.239 |
| I105A | 75 | total | 800 | 0.257 |
| I105A | 100 | total | 800 | 0.112 |
| I105A | 150 | total | 800 | 0.016 |
| I105A | 200 | total | 1000 | 0.009 |
| I105A | 5 | total | 500 | 0.167 |
| I105A | 12 | total | 500 | 0.238 |
| I105A | 25 | total | 500 | 0.000 |
| I105A | 41 | total | 500 | 0.297 |
| I105A | 75 | total | 500 | 0.125 |
| I106A | 0 | m | 800 | 0.003 |
| I106A | 0 | n | 800 | 0.048 |
| I106A | 0 | p | 800 | 0.087 |
| I106A | 25 | m | 800 | 0.003 |
| I106A | 25 | n | 800 | 0.031 |
| I106A | 25 | p | 800 | 0.150 |
| I106A | 50 | m | 800 | 0.004 |
| I106A | 50 | n | 800 | 0.131 |
| I106A | 50 | p | 800 | 0.202 |
| I106A | 75 | m | 800 | 0.011 |
| I106A | 75 | n | 800 | 0.264 |
| I106A | 75 | p | 800 | 0.315 |
| I106A | 100 | m | 800 | 0.003 |
| I106A | 100 | n | 800 | 0.019 |
| I106A | 100 | p | 800 | 0.115 |
| I106A | 150 | m | 800 | 0.002 |
| I106A | 150 | n | 800 | 0.007 |
| I106A | 150 | p | 800 | 0.002 |
| I106A | 200 | m | 800 | 0.002 |
| I106A | 200 | n | 800 | 0.007 |
| I106A | 200 | p | 800 | 0.002 |
| I201 | 5 | total | 800 | 0.117 |
| I201 | 25 | total | 800 | 0.151 |
| I201 | 50 | total | 800 | 0.413 |
| I201 | 75 | total | 800 | 0.443 |
| I201 | 100 | total | 800 | 0.115 |
| I201 | 150 | total | 800 | 0.010 |
| I201 | 200 | total | 800 | 0.008 |
| I202 | 0 | m | 800 | 0.007 |
| I202 | 0 | n | 800 | 0.068 |
| I202 | 0 | p | 800 | 0.061 |
| I202 | 25 | m | 800 | 0.002 |
| I202 | 25 | n | 800 | 0.012 |
| I202 | 25 | p | 800 | 0.125 |
| I202 | 50 | m | 500 | 0.003 |
| I202 | 50 | n | 500 | 0.036 |
| I202 | 50 | p | 500 | 0.216 |
| I202 | 75 | m | 800 | 0.020 |
| I202 | 75 | n | 800 | 0.151 |
| I202 | 75 | p | 800 | 0.411 |
| I202 | 100 | m | 800 | 0.005 |
| I202 | 100 | n | 800 | 0.026 |
| I202 | 100 | p | 800 | 0.187 |
| I202 | 150 | m | 800 | 0.001 |
| I202 | 150 | n | 800 | 0.007 |
| I202 | 150 | p | 800 | 0.006 |
| I202 | 200 | m | 800 | 0.001 |
| I202 | 200 | n | 800 | 0.005 |
| I202 | 200 | p | 800 | 0.005 |
| I203 | 5 | total | 500 | 0.143 |
| I203 | 25 | total | 500 | 0.476 |
| I203 | 50 | total | 500 | 0.588 |
| I203 | 75 | total | 500 | 0.496 |
| I203 | 100 | total | 500 | 0.095 |
| I203 | 150 | total | 500 | 0.013 |
| I203 | 200 | total | 500 | 0.010 |
| I203 | 0 | m | 800 | 0.004 |
| I203 | 0 | n | 800 | 0.038 |
| I203 | 0 | p | 800 | 0.108 |
| I204 | 5 | m | 800 | 0.005 |
| I204 | 5 | n | 800 | 0.013 |
| I204 | 5 | p | 800 | 0.134 |
| I204 | 25 | m | 800 | 0.003 |
| I204 | 25 | n | 800 | 0.024 |
| I204 | 25 | p | 800 | 0.151 |
| I204 | 50 | m | 800 | 0.021 |
| I204 | 50 | n | 800 | 0.080 |
| I204 | 50 | p | 800 | 0.417 |
| I204 | 75 | m | 800 | 0.011 |
| I204 | 75 | n | 800 | 0.114 |
| I204 | 75 | p | 800 | 0.189 |
| I204 | 100 | m | 800 | 0.003 |
| I204 | 100 | n | 800 | 0.022 |
| I204 | 100 | p | 800 | 0.080 |
| I204 | 150 | m | 800 | 0.002 |
| I204 | 150 | n | 800 | 0.008 |
| I204 | 150 | p | 800 | 0.006 |
| I204 | 200 | m | 800 | 0.001 |
| I204 | 200 | n | 800 | 0.007 |
| I204 | 200 | p | 800 | 0.001 |
| I205 | 5 | total | 800 | 0.110 |
| I205 | 25 | total | 800 | 0.263 |
| I205 | 50 | total | 800 | 0.681 |
| I205 | 75 | total | 800 | 0.349 |
| I205 | 100 | total | 800 | 0.215 |
| I205 | 150 | total | 800 | 0.020 |
| I205 | 200 | total | 800 | 0.008 |
| I206 | 5 | m | 800 | 0.004 |
| I206 | 5 | n | 800 | 0.065 |
| I206 | 5 | p | 800 | 0.076 |
| I206 | 25 | m | 800 | 0.005 |
| I206 | 25 | n | 800 | 0.048 |
| I206 | 25 | p | 800 | 0.112 |
| I206 | 75 | m | 800 | 0.012 |
| I206 | 75 | n | 800 | 0.132 |
| I206 | 75 | p | 800 | 0.147 |
| I206 | 100 | m | 800 | 0.008 |
| I206 | 100 | n | 800 | 0.059 |
| I206 | 100 | p | 800 | 0.085 |
| I206 | 150 | m | 800 | 0.005 |
| I206 | 150 | n | 800 | 0.015 |
| I206 | 150 | p | 800 | 0.002 |
| I206 | 200 | m | 800 | 0.010 |
| I206 | 200 | n | 800 | 0.021 |
| I206 | 200 | p | 800 | 0.004 |
| I207 | 5 | total | 500 | 0.132 |
| I207 | 25 | total | 500 | 0.208 |
| I207 | 50 | total | 500 | 0.400 |
| I207 | 75 | total | 500 | 0.655 |
| I207 | 100 | total | 500 | 0.088 |
| I207 | 150 | total | 500 | 0.012 |
| I207 | 200 | total | 500 | 0.014 |
| I208 | 5 | m | 800 | 0.001 |
| I208 | 5 | n | 800 | 0.026 |
| I208 | 5 | p | 800 | 0.067 |
| I208 | 25 | m | 800 | 0.001 |
| I208 | 25 | n | 800 | 0.023 |
| I208 | 25 | p | 800 | 0.071 |
| I208 | 50 | m | 800 | 0.003 |
| I208 | 50 | n | 800 | 0.045 |
| I208 | 50 | p | 800 | 0.113 |
| I208 | 75 | m | 800 | 0.024 |
| I208 | 75 | n | 800 | 0.191 |
| I208 | 75 | p | 800 | 0.313 |
| I208 | 100 | m | 800 | 0.004 |
| I208 | 100 | n | 800 | 0.056 |
| I208 | 100 | p | 800 | 0.040 |
| I208 | 150 | m | 800 | 0.002 |
| I208 | 150 | n | 800 | 0.007 |
| I208 | 150 | p | 800 | 0.003 |
| I208 | 200 | m | 800 | 0.001 |
| I208 | 200 | n | 800 | 0.005 |
| I208 | 200 | p | 800 | 0.003 |
| I208 | 0 | m | 800 | 0.005 |
| I208 | 0 | n | 800 | 0.031 |
| I208 | 0 | p | 800 | 0.069 |
| I208 | 0 | total | 800 | 0.096 |
| I209 | 5 | total | 500 | 0.137 |
| I209 | 25 | total | 500 | 0.148 |
| I209 | 50 | total | 500 | 0.333 |
| I209 | 75 | total | 500 | 0.435 |
| I209 | 100 | total | 500 | 0.202 |
| I209 | 150 | total | 500 | 0.016 |
| I209 | 200 | total | 500 | 0.011 |
| I210 | 5 | m | 500 | 0.003 |
| I210 | 5 | n | 500 | 0.018 |
| I210 | 5 | p | 500 | 0.115 |
| I210 | 25 | m | 800 | 0.004 |
| I210 | 25 | n | 800 | 0.121 |
| I210 | 25 | p | 800 | 0.083 |
| I210 | 75 | m | 800 | 0.022 |
| I210 | 75 | n | 800 | 0.648 |
| I210 | 75 | p | 800 | 0.069 |
| I210 | 100 | m | 800 | 0.004 |
| I210 | 100 | n | 800 | 0.084 |
| I210 | 100 | p | 800 | 0.039 |
| I210 | 150 | m | 800 | 0.002 |
| I210 | 150 | n | 800 | 0.018 |
| I210 | 150 | p | 800 | 0.004 |
| I210 | 200 | m | 800 | 0.001 |
| I210 | 200 | n | 800 | 0.007 |
| I210 | 200 | p | 800 | 0.001 |
| I211 | 5 | total | 800 | 0.134 |
| I211 | 25 | total | 800 | 0.163 |
| I211 | 50 | total | 800 | 0.405 |
| I211 | 75 | total | 800 | 0.462 |
| I211 | 100 | total | 800 | 0.098 |
| I211 | 150 | total | 800 | 0.012 |
| I211 | 200 | total | 800 | 0.008 |
| I212 | 5 | m | 800 | 0.002 |
| I212 | 5 | n | 800 | 0.016 |
| I212 | 5 | p | 800 | 0.122 |
| I212 | 25 | m | 800 | 0.002 |
| I212 | 25 | n | 800 | 0.031 |
| I212 | 25 | p | 800 | 0.161 |
| I212 | 50 | m | 800 | 0.013 |
| I212 | 50 | n | 800 | 0.044 |
| I212 | 50 | p | 800 | 0.410 |
| I212 | 75 | m | 800 | 0.010 |
| I212 | 75 | n | 800 | 0.095 |
| I212 | 75 | p | 800 | 0.324 |
| I212 | 100 | m | 800 | 0.004 |
| I212 | 100 | n | 800 | 0.014 |
| I212 | 100 | p | 800 | 0.086 |
| I212 | 150 | m | 800 | 0.002 |
| I212 | 150 | n | 800 | 0.007 |
| I212 | 150 | p | 800 | 0.007 |
| I212 | 200 | m | 410 | 0.002 |
| I212 | 200 | n | 410 | 0.003 |
| I212 | 200 | p | 410 | 0.004 |
| I213 | 5 | total | 800 | 0.125 |
| I213 | 25 | total | 800 | 0.129 |
| I213 | 50 | total | 800 | 0.314 |
| I213 | 75 | total | 800 | 0.447 |
| I213 | 100 | total | 800 | 0.031 |
| I213 | 150 | total | 800 | 0.015 |
| I213 | 200 | total | 800 | 0.008 |
| I213 | 0 | m | 800 | 0.002 |
| I213 | 0 | n | 800 | 0.017 |
| I213 | 0 | p | 800 | 0.104 |
| I214 | 0 | m | 800 | 0.001 |
| I214 | 0 | n | 800 | 0.015 |
| I214 | 0 | p | 800 | 0.083 |
| I214 | 25 | m | 800 | 0.002 |
| I214 | 25 | n | 800 | 0.014 |
| I214 | 25 | p | 800 | 0.128 |
| I214 | 50 | m | 500 | 0.021 |
| I214 | 50 | n | 500 | 0.041 |
| I214 | 50 | p | 500 | 0.176 |
| I214 | 75 | m | 800 | 0.003 |
| I214 | 75 | n | 800 | 0.014 |
| I214 | 75 | p | 800 | 0.167 |
| I214 | 100 | m | 800 | 0.002 |
| I214 | 100 | n | 800 | 0.009 |
| I214 | 100 | p | 800 | 0.043 |
| I214 | 150 | m | 800 | 0.002 |
| I214 | 150 | n | 800 | 0.007 |
| I214 | 150 | p | 800 | 0.012 |
| I214 | 200 | m | 690 | 0.002 |
| I214 | 200 | n | 690 | 0.006 |
| I214 | 200 | p | 690 | 0.003 |
| I712 | 0 | m | 800 | 0.005 |
| I712 | 0 | n | 800 | 0.014 |
| I712 | 0 | p | 800 | 0.167 |
| I712 | 25 | m | 800 | 0.004 |
| I712 | 25 | n | 800 | 0.014 |
| I712 | 25 | p | 800 | 0.173 |
| I712 | 50 | m | 510 | 0.007 |
| I712 | 50 | n | 510 | 0.017 |
| I712 | 50 | p | 510 | 0.298 |
| I712 | 75 | m | 800 | 0.012 |
| I712 | 75 | n | 800 | 0.027 |
| I712 | 75 | p | 800 | 0.267 |
| I712 | 100 | m | 800 | 0.004 |
| I712 | 100 | n | 800 | 0.020 |
| I712 | 100 | p | 800 | 0.130 |
| I712 | 150 | m | 800 | 0.002 |
| I712 | 150 | n | 800 | 0.005 |
| I712 | 150 | p | 800 | 0.004 |
| I712 | 200 | m | 800 | 0.002 |
| I712 | 200 | n | 800 | 0.006 |
| I712 | 200 | p | 800 | 0.001 |
| I713 | 5 | total | 500 | 0.087 |
| I713 | 25 | total | 500 | 0.104 |
| I713 | 50 | total | 500 | 0.305 |
| I713 | 75 | total | 500 | 0.383 |
| I713 | 100 | total | 500 | 0.152 |
| I713 | 150 | total | 500 | 0.009 |
| I713 | 200 | total | 500 | 0.009 |
| I714 | 0 | m | 800 | 0.002 |
| I714 | 0 | n | 800 | 0.010 |
| I714 | 0 | p | 800 | 0.106 |
| I714 | 25 | m | 700 | 0.002 |
| I714 | 25 | n | 700 | 0.020 |
| I714 | 25 | p | 700 | 0.126 |
| I714 | 50 | m | 400 | 0.018 |
| I714 | 50 | n | 400 | 0.095 |
| I714 | 50 | p | 400 | 0.402 |
| I714 | 75 | m | 500 | 0.017 |
| I714 | 75 | n | 500 | 0.172 |
| I714 | 75 | p | 500 | 0.318 |
| I714 | 100 | m | 700 | 0.005 |
| I714 | 100 | n | 700 | 0.120 |
| I714 | 100 | p | 700 | 0.107 |
| I714 | 150 | m | 500 | 0.004 |
| I714 | 150 | n | 500 | 0.030 |
| I714 | 150 | p | 500 | 0.029 |
| I714 | 200 | m | 500 | 0.001 |
| I714 | 200 | n | 500 | 0.007 |
| I714 | 200 | p | 500 | 0.004 |
| I715 | 5 | total | 500 | 0.078 |
| I715 | 25 | total | 500 | 0.078 |
| I715 | 50 | total | 500 | 0.120 |
| I715 | 75 | total | 500 | 0.346 |
| I715 | 100 | total | 500 | 0.392 |
| I715 | 150 | total | 500 | 0.061 |
| I715 | 200 | total | 500 | 0.009 |
| I701 | 0 | m | 800 | 0.002 |
| I701 | 0 | n | 800 | 0.008 |
| I701 | 0 | p | 800 | 0.086 |
| I701 | 10 | m | 800 | 0.001 |
| I701 | 10 | n | 800 | 0.007 |
| I701 | 10 | p | 800 | 0.077 |
| I701 | 25 | m | 800 | 0.001 |
| I701 | 25 | n | 800 | 0.029 |
| I701 | 25 | p | 800 | 0.066 |
| I701 | 50 | m | 800 | 0.002 |
| I701 | 50 | n | 800 | 0.023 |
| I701 | 50 | p | 800 | 0.103 |
| I701 | 75 | m | 800 | 0.009 |
| I701 | 75 | n | 800 | 0.256 |
| I701 | 75 | p | 800 | 0.164 |
| I701 | 100 | m | 800 | 0.004 |
| I701 | 100 | n | 800 | 0.044 |
| I701 | 100 | p | 800 | 0.188 |
| I701 | 150 | m | 800 | 0.001 |
| I701 | 150 | n | 800 | 0.023 |
| I701 | 150 | p | 800 | 0.014 |
| I701 | 200 | m | 800 | 0.001 |
| I701 | 200 | n | 800 | 0.006 |
| I701 | 200 | p | 800 | 0.006 |
| I703 | 0 | total | 800 | 0.079 |
| I703 | 25 | total | 500 | 0.091 |
| I703 | 50 | total | 500 | 0.192 |
| I703 | 75 | total | 500 | 0.568 |
| I703 | 100 | total | 500 | 0.344 |
| I703 | 150 | total | 500 | 0.038 |
| I703 | 200 | total | 500 | 0.013 |
| I703 | 0 | m | 800 | 0.002 |
| I703 | 0 | n | 800 | 0.009 |
| I703 | 0 | p | 800 | 0.069 |
| I705 | 5 | m | 800 | 0.001 |
| I705 | 5 | n | 800 | 0.006 |
| I705 | 5 | p | 800 | 0.081 |
| I705 | 25 | m | 800 | 0.001 |
| I705 | 25 | n | 800 | 0.010 |
| I705 | 25 | p | 800 | 0.089 |
| I705 | 50 | m | 800 | 0.006 |
| I705 | 50 | n | 800 | 0.019 |
| I705 | 50 | p | 800 | 0.305 |
| I705 | 75 | m | 800 | 0.009 |
| I705 | 75 | n | 800 | 0.036 |
| I705 | 75 | p | 800 | 0.369 |
| I705 | 100 | m | 800 | 0.004 |
| I705 | 100 | n | 800 | 0.022 |
| I705 | 100 | p | 800 | 0.236 |
| I705 | 150 | m | 800 | 0.001 |
| I705 | 150 | n | 800 | 0.007 |
| I705 | 150 | p | 800 | 0.012 |
| I705 | 200 | m | 800 | 0.001 |
| I705 | 200 | n | 800 | 0.004 |
| I705 | 200 | p | 800 | 0.003 |
| I707 | 5 | total | 500 | 0.070 |
| I707 | 25 | total | 500 | 0.096 |
| I707 | 50 | total | 500 | 0.403 |
| I707 | 75 | total | 500 | 0.285 |
| I707 | 100 | total | 500 | 0.099 |
| I707 | 150 | total | 500 | 0.012 |
| I707 | 200 | total | 500 | 0.007 |
| I707 | 0 | m | 800 | 0.001 |
| I707 | 0 | n | 800 | 0.006 |
| I707 | 0 | p | 800 | 0.062 |
| I709 | 5 | m | 800 | 0.002 |
| I709 | 5 | n | 800 | 0.008 |
| I709 | 5 | p | 800 | 0.108 |
| I709 | 25 | m | 800 | 0.002 |
| I709 | 25 | n | 800 | 0.008 |
| I709 | 25 | p | 800 | 0.124 |
| I709 | 50 | m | 800 | 0.007 |
| I709 | 50 | n | 800 | 0.019 |
| I709 | 50 | p | 800 | 0.264 |
| I709 | 75 | m | 800 | 0.010 |
| I709 | 75 | n | 800 | 0.045 |
| I709 | 75 | p | 800 | 0.336 |
| I709 | 100 | m | 800 | 0.005 |
| I709 | 100 | n | 800 | 0.022 |
| I709 | 100 | p | 800 | 0.173 |
| I709 | 150 | m | 800 | 0.002 |
| I709 | 150 | n | 800 | 0.007 |
| I709 | 150 | p | 800 | 0.004 |
| I709 | 200 | m | 800 | 0.002 |
| I709 | 200 | n | 800 | 0.005 |
| I709 | 200 | p | 800 | 0.003 |
| I322 | 5 | total | 500 | 0.097 |
| I322 | 25 | total | 500 | 0.112 |
| I322 | 50 | total | 500 | 0.528 |
| I322 | 75 | total | 500 | 0.331 |
| I322 | 100 | total | 500 | 0.153 |
| I322 | 150 | total | 500 | 0.011 |
| I322 | 200 | total | 500 | 0.009 |
| I322 | 0 | m | 800 | 0.001 |
| I322 | 0 | n | 800 | 0.007 |
| I322 | 0 | p | 800 | 0.090 |
| I321 | 0 | total | 500 | 0.142 |
| I321 | 10 | total | 500 | 0.140 |
| I321 | 25 | total | 500 | 0.146 |
| I321 | 40 | total | 500 | 0.245 |
| I321 | 75 | total | 500 | 0.381 |
| I321 | 100 | total | 500 | 0.134 |
| I321 | 150 | total | 500 | 0.009 |
| I321 | 200 | total | 500 | 0.009 |
| I320 | 0 | m | 800 | 0.002 |
| I320 | 0 | n | 800 | 0.011 |
| I320 | 0 | p | 800 | 0.093 |
| I320 | 25 | m | 800 | 0.002 |
| I320 | 25 | n | 800 | 0.014 |
| I320 | 25 | p | 800 | 0.136 |
| I320 | 50 | m | 800 | 0.021 |
| I320 | 50 | n | 800 | 0.060 |
| I320 | 50 | p | 800 | 0.367 |
| I320 | 75 | m | 800 | 0.015 |
| I320 | 75 | n | 800 | 0.044 |
| I320 | 75 | p | 800 | 0.354 |
| I320 | 100 | m | 800 | 0.006 |
| I320 | 100 | n | 800 | 0.023 |
| I320 | 100 | p | 800 | 0.215 |
| I320 | 150 | m | 500 | 0.002 |
| I320 | 150 | n | 500 | 0.009 |
| I320 | 150 | p | 500 | 0.008 |
| I320 | 200 | m | 800 | 0.001 |
| I320 | 200 | n | 800 | 0.006 |
| I320 | 200 | p | 800 | 0.005 |
| I318 | 5 | total | 500 | 0.109 |
| I318 | 25 | total | 500 | 0.126 |
| I318 | 50 | total | 500 | 0.317 |
| I318 | 75 | total | 500 | 0.341 |
| I318 | 100 | total | 500 | 0.159 |
| I318 | 150 | total | 500 | 0.013 |
| I318 | 200 | total | 500 | 0.010 |
| I318 | 0 | m | 800 | 0.001 |
| I318 | 0 | n | 800 | 0.009 |
| I318 | 0 | p | 800 | 0.108 |
| I316 | 0 | m | 800 | 0.002 |
| I316 | 0 | n | 800 | 0.008 |
| I316 | 0 | p | 800 | 0.087 |
| I316 | 25 | m | 510 | 0.004 |
| I316 | 25 | n | 510 | 0.012 |
| I316 | 25 | p | 510 | 0.101 |
| I316 | 50 | m | 755 | 0.009 |
| I316 | 50 | n | 755 | 0.027 |
| I316 | 50 | p | 755 | 0.223 |
| I316 | 75 | m | 705 | 0.017 |
| I316 | 75 | n | 705 | 0.045 |
| I316 | 75 | p | 705 | 0.328 |
| I316 | 100 | m | 690 | 0.006 |
| I316 | 100 | n | 680 | 0.030 |
| I316 | 100 | p | 680 | 0.243 |
| I316 | 150 | m | 800 | 0.003 |
| I316 | 150 | n | 800 | 0.011 |
| I316 | 150 | p | 800 | 0.041 |
| I316 | 200 | m | 800 | 0.001 |
| I316 | 200 | n | 800 | 0.005 |
| I316 | 200 | p | 800 | 0.003 |
| I314 | 5 | total | 500 | 0.129 |
| I314 | 10 | total | 500 | 0.135 |
| I314 | 25 | total | 500 | 0.133 |
| I314 | 40 | total | 500 | 0.146 |
| I314 | 75 | total | 500 | 0.310 |
| I314 | 100 | total | 500 | 0.282 |
| I314 | 150 | total | 500 | 0.089 |
| I314 | 200 | total | 500 | 0.010 |
| I312 | 5 | m | 800 | 0.001 |
| I312 | 5 | n | 800 | 0.009 |
| I312 | 5 | p | 800 | 0.091 |
| I312 | 25 | m | 800 | 0.001 |
| I312 | 25 | n | 800 | 0.006 |
| I312 | 25 | p | 800 | 0.096 |
| I312 | 50 | m | 800 | 0.002 |
| I312 | 50 | n | 800 | 0.017 |
| I312 | 50 | p | 800 | 0.154 |
| I312 | 75 | m | 800 | 0.029 |
| I312 | 75 | n | 800 | 0.154 |
| I312 | 75 | p | 800 | 0.072 |
| I312 | 100 | m | 800 | 0.013 |
| I312 | 100 | n | 800 | 0.030 |
| I312 | 100 | p | 800 | 0.296 |
| I312 | 150 | m | 800 | 0.003 |
| I312 | 150 | n | 800 | 0.011 |
| I312 | 150 | p | 800 | 0.030 |
| I312 | 200 | m | 800 | 0.002 |
| I312 | 200 | n | 800 | 0.005 |
| I312 | 200 | p | 800 | 0.004 |
| I401 | 0 | m | 800 | 0.002 |
| I401 | 0 | n | 800 | 0.014 |
| I401 | 0 | p | 800 | 0.206 |
| I401 | 25 | m | 800 | 0.002 |
| I401 | 25 | n | 800 | 0.012 |
| I401 | 25 | p | 800 | 0.197 |
| I401 | 50 | m | 800 | 0.002 |
| I401 | 50 | n | 800 | 0.019 |
| I401 | 50 | p | 800 | 0.248 |
| I401 | 75 | m | 800 | 0.042 |
| I401 | 75 | n | 800 | 0.061 |
| I401 | 75 | p | 800 | 0.488 |
| I401 | 100 | m | 800 | 0.017 |
| I401 | 100 | n | 800 | 0.044 |
| I401 | 100 | p | 800 | 0.291 |
| I401 | 150 | m | 800 | 0.002 |
| I401 | 150 | n | 800 | 0.007 |
| I401 | 150 | p | 800 | 0.010 |
| I401 | 200 | m | 800 | 0.001 |
| I401 | 200 | n | 800 | 0.004 |
| I401 | 200 | p | 800 | 0.003 |
| I309 | 5 | total | 500 | 0.107 |
| I309 | 25 | total | 500 | 0.110 |
| I309 | 50 | total | 500 | 0.219 |
| I309 | 75 | total | 500 | 0.682 |
| I309 | 100 | total | 500 | 0.246 |
| I309 | 150 | total | 500 | 0.013 |
| I309 | 200 | total | 500 | 0.014 |
| I307 | 0 | m | 800 | 0.002 |
| I307 | 0 | n | 800 | 0.009 |
| I307 | 0 | p | 800 | 0.098 |
| I307 | 25 | m | 800 | 0.002 |
| I307 | 25 | n | 800 | 0.013 |
| I307 | 25 | p | 800 | 0.111 |
| I307 | 50 | m | 800 | 0.004 |
| I307 | 50 | n | 800 | 0.016 |
| I307 | 50 | p | 800 | 0.202 |
| I307 | 75 | m | 800 | 0.024 |
| I307 | 75 | n | 800 | 0.053 |
| I307 | 75 | p | 800 | 0.503 |
| I307 | 100 | m | 800 | 0.007 |
| I307 | 100 | n | 800 | 0.023 |
| I307 | 100 | p | 800 | 0.222 |
| I307 | 150 | m | 800 | 0.001 |
| I307 | 150 | n | 800 | 0.007 |
| I307 | 150 | p | 800 | 0.006 |
| I307 | 200 | m | 800 | 0.001 |
| I307 | 200 | n | 800 | 0.007 |
| I307 | 200 | p | 800 | 0.003 |
| I301 | 5 | total | 500 | 0.130 |
| I301 | 25 | total | 500 | 0.144 |
| I301 | 50 | total | 500 | 0.341 |
| I301 | 75 | total | 500 | 0.393 |
| I301 | 100 | total | 500 | 0.193 |
| I301 | 150 | total | 500 | 0.034 |
| I301 | 200 | total | 500 | 0.010 |
| I301 | 0 | m | 800 | 0.004 |
| I301 | 0 | n | 800 | 0.014 |
| I301 | 0 | p | 800 | 0.128 |
| I303 | 5 | m | 800 | 0.003 |
| I303 | 5 | n | 800 | 0.014 |
| I303 | 5 | p | 800 | 0.097 |
| I303 | 25 | m | 800 | 0.004 |
| I303 | 25 | n | 800 | 0.020 |
| I303 | 25 | p | 800 | 0.142 |
| I303 | 50 | m | 800 | 0.040 |
| I303 | 50 | n | 800 | 0.064 |
| I303 | 50 | p | 800 | 0.388 |
| I303 | 75 | m | 800 | 0.051 |
| I303 | 75 | n | 800 | 0.086 |
| I303 | 75 | p | 800 | 0.356 |
| I303 | 100 | m | 800 | 0.005 |
| I303 | 100 | n | 800 | 0.024 |
| I303 | 100 | p | 800 | 0.122 |
| I303 | 150 | m | 800 | 0.002 |
| I303 | 150 | n | 800 | 0.010 |
| I303 | 150 | p | 800 | 0.019 |
| I303 | 200 | m | 800 | 0.002 |
| I303 | 200 | n | 800 | 0.006 |
| I303 | 200 | p | 800 | 0.002 |
| I305 | 5 | total | 500 | 0.133 |
| I305 | 10 | total | 500 | 0.125 |
| I305 | 25 | total | 500 | 0.139 |
| I305 | 40 | total | 500 | 0.204 |
| I305 | 75 | total | 500 | 0.460 |
| I305 | 100 | total | 500 | 0.205 |
| I305 | 150 | total | 500 | 0.050 |
| I305 | 200 | total | 500 | 0.013 |
| I402 | 5 | total | 500 | 0.126 |
| I402 | 25 | total | 500 | 0.152 |
| I402 | 50 | total | 500 | 0.217 |
| I402 | 75 | total | 500 | 0.568 |
| I402 | 100 | total | 500 | 0.249 |
| I402 | 150 | total | 500 | 0.032 |
| I402 | 200 | total | 500 | 0.012 |
| I403 | 0 | m | 800 | 0.002 |
| I403 | 0 | n | 800 | 0.010 |
| I403 | 0 | p | 800 | 0.087 |
| I403 | 25 | m | 800 | 0.002 |
| I403 | 25 | n | 800 | 0.010 |
| I403 | 25 | p | 800 | 0.094 |
| I403 | 50 | m | 800 | 0.003 |
| I403 | 50 | n | 800 | 0.016 |
| I403 | 50 | p | 800 | 0.150 |
| I403 | 75 | m | 800 | 0.005 |
| I403 | 75 | n | 800 | 0.078 |
| I403 | 75 | p | 800 | 0.270 |
| I403 | 100 | m | 800 | 0.015 |
| I403 | 100 | n | 800 | 0.069 |
| I403 | 100 | p | 800 | 0.337 |
| I403 | 150 | m | 800 | 0.003 |
| I403 | 150 | n | 800 | 0.015 |
| I403 | 150 | p | 800 | 0.066 |
| I403 | 200 | m | 800 | 0.001 |
| I403 | 200 | n | 800 | 0.006 |
| I403 | 200 | p | 800 | 0.003 |
| I404 | 5 | total | 500 | 0.029 |
| I404 | 25 | total | 500 | 0.151 |
| I404 | 50 | total | 500 | 0.232 |
| I404 | 75 | total | 500 | 0.565 |
| I404 | 100 | total | 500 | 0.286 |
| I404 | 150 | total | 500 | 0.043 |
| I404 | 200 | total | 500 | 0.010 |
| I404 | 0 | m | 800 | 0.003 |
| I404 | 0 | n | 800 | 0.013 |
| I404 | 0 | p | 800 | 0.106 |
| I405 | 0 | m | 800 | 0.001 |
| I405 | 0 | n | 800 | 0.012 |
| I405 | 0 | p | 800 | 0.068 |
| I405 | 25 | m | 800 | 0.003 |
| I405 | 25 | n | 800 | 0.011 |
| I405 | 25 | p | 800 | 0.068 |
| I405 | 50 | m | 800 | 0.004 |
| I405 | 50 | n | 800 | 0.017 |
| I405 | 50 | p | 800 | 0.111 |
| I405 | 75 | m | 800 | 0.061 |
| I405 | 75 | n | 800 | 0.109 |
| I405 | 75 | p | 800 | 0.353 |
| I405 | 100 | m | 800 | 0.014 |
| I405 | 100 | n | 800 | 0.049 |
| I405 | 100 | p | 800 | 0.240 |
| I405 | 150 | m | 800 | 0.001 |
| I405 | 150 | n | 800 | 0.009 |
| I405 | 150 | p | 800 | 0.025 |
| I405 | 200 | m | 800 | 0.001 |
| I405 | 200 | n | 800 | 0.007 |
| I405 | 200 | p | 800 | 0.003 |
| I406 | 5 | total | 500 | 0.108 |
| I406 | 25 | total | 500 | 0.106 |
| I406 | 50 | total | 500 | 0.122 |
| I406 | 75 | total | 500 | 0.516 |
| I406 | 100 | total | 500 | 0.359 |
| I406 | 150 | total | 500 | 0.042 |
| I406 | 200 | total | 500 | 0.010 |
| I407 | 0 | m | 800 | 0.005 |
| I407 | 0 | n | 800 | 0.019 |
| I407 | 0 | p | 800 | 0.058 |
| I407 | 25 | m | 800 | 0.001 |
| I407 | 25 | n | 800 | 0.016 |
| I407 | 25 | p | 800 | 0.078 |
| I407 | 50 | m | 800 | 0.002 |
| I407 | 50 | n | 800 | 0.015 |
| I407 | 50 | p | 800 | 0.108 |
| I407 | 75 | m | 800 | 0.009 |
| I407 | 75 | n | 800 | 0.041 |
| I407 | 75 | p | 800 | 0.185 |
| I407 | 100 | m | 800 | 0.019 |
| I407 | 100 | n | 800 | 0.070 |
| I407 | 100 | p | 800 | 0.363 |
| I407 | 150 | m | 800 | 0.003 |
| I407 | 150 | n | 800 | 0.014 |
| I407 | 150 | p | 800 | 0.057 |
| I407 | 200 | m | 800 | 0.001 |
| I407 | 200 | n | 800 | 0.007 |
| I407 | 200 | p | 800 | 0.002 |
| I407 | 0 | total | 500 | 0.079 |
| I408 | 5 | total | 500 | 0.091 |
| I408 | 25 | total | 500 | 0.098 |
| I408 | 50 | total | 500 | 0.120 |
| I408 | 75 | total | 500 | 0.227 |
| I408 | 100 | total | 500 | 0.340 |
| I408 | 150 | total | 500 | 0.066 |
| I408 | 200 | total | 500 | 0.010 |
| I408 | 5 | total | 500 | 0.102 |
| I408 | 10 | total | 500 | 0.107 |
| I408 | 25 | total | 500 | 0.109 |
| I408 | 40 | total | 500 | 0.110 |
| I408 | 75 | total | 500 | 0.174 |
| I409 | 0 | m | 800 | 0.001 |
| I409 | 0 | n | 800 | 0.010 |
| I409 | 0 | p | 800 | 0.063 |
| I409 | 25 | m | 800 | 0.001 |
| I409 | 25 | n | 800 | 0.011 |
| I409 | 25 | p | 800 | 0.061 |
| I409 | 50 | m | 800 | 0.002 |
| I409 | 50 | n | 800 | 0.013 |
| I409 | 50 | p | 800 | 0.085 |
| I409 | 75 | m | 800 | 0.003 |
| I409 | 75 | n | 800 | 0.020 |
| I409 | 75 | p | 800 | 0.170 |
| I409 | 100 | m | 800 | 0.028 |
| I409 | 100 | n | 800 | 0.058 |
| I409 | 100 | p | 800 | 0.286 |
| I409 | 150 | m | 800 | 0.002 |
| I409 | 150 | n | 800 | 0.013 |
| I409 | 150 | p | 800 | 0.034 |
| I409 | 200 | m | 800 | 0.002 |
| I409 | 200 | n | 800 | 0.011 |
| I409 | 200 | p | 800 | 0.003 |
| I410 | 5 | total | 500 | 0.096 |
| I410 | 25 | total | 500 | 0.091 |
| I410 | 50 | total | 500 | 0.135 |
| I410 | 75 | total | 500 | 0.167 |
| I410 | 100 | total | 500 | 0.513 |
| I410 | 150 | total | 500 | 0.055 |
| I410 | 200 | total | 500 | 0.011 |
| I410 | 0 | m | 800 | 0.001 |
| I410 | 0 | n | 800 | 0.011 |
| I410 | 0 | p | 800 | 0.089 |
| I411 | 0 | m | 800 | 0.039 |
| I411 | 0 | n | 800 | 0.017 |
| I411 | 0 | p | 800 | 0.065 |
| I411 | 25 | m | 800 | 0.002 |
| I411 | 25 | n | 800 | 0.014 |
| I411 | 25 | p | 800 | 0.067 |
| I411 | 50 | m | 800 | 0.003 |
| I411 | 50 | n | 800 | 0.012 |
| I411 | 50 | p | 800 | 0.094 |
| I411 | 75 | m | 800 | 0.007 |
| I411 | 75 | n | 800 | 0.022 |
| I411 | 75 | p | 800 | 0.189 |
| I411 | 100 | m | 800 | 0.022 |
| I411 | 100 | n | 800 | 0.079 |
| I411 | 100 | p | 800 | 0.633 |
| I411 | 150 | m | 800 | 0.002 |
| I411 | 150 | n | 800 | 0.029 |
| I411 | 150 | p | 800 | 0.026 |
| I411 | 200 | m | 800 | 0.001 |
| I411 | 200 | n | 800 | 0.007 |
| I411 | 200 | p | 800 | 0.005 |
| I412 | 5 | total | 800 | 0.100 |
| I412 | 25 | total | 800 | 0.101 |
| I412 | 50 | total | 800 | 0.129 |
| I412 | 75 | total | 800 | 0.194 |
| I412 | 100 | total | 800 | 0.680 |
| I412 | 150 | total | 800 | 0.079 |
| I412 | 200 | total | 800 | 0.014 |
| I413 | 0 | m | 800 | 0.004 |
| I413 | 0 | n | 800 | 0.015 |
| I413 | 0 | p | 800 | 0.074 |
| I413 | 25 | m | 800 | 0.002 |
| I413 | 25 | n | 800 | 0.010 |
| I413 | 25 | p | 800 | 0.073 |
| I413 | 50 | m | 800 | 0.002 |
| I413 | 50 | n | 800 | 0.014 |
| I413 | 50 | p | 800 | 0.111 |
| I413 | 75 | m | 800 | 0.004 |
| I413 | 75 | n | 800 | 0.020 |
| I413 | 75 | p | 800 | 0.147 |
| I413 | 100 | m | 800 | 0.020 |
| I413 | 100 | n | 800 | 0.098 |
| I413 | 100 | p | 800 | 0.527 |
| I413 | 150 | m | 800 | 0.003 |
| I413 | 150 | n | 800 | 0.013 |
| I413 | 150 | p | 800 | 0.065 |
| I413 | 200 | m | 800 | 0.003 |
| I413 | 200 | n | 800 | 0.008 |
| I413 | 200 | p | 800 | 0.006 |
| I414 | 5 | total | 500 | 0.096 |
| I414 | 25 | total | 500 | 0.080 |
| I414 | 50 | total | 500 | 0.118 |
| I414 | 75 | total | 500 | 0.160 |
| I414 | 100 | total | 500 | 0.325 |
| I414 | 150 | total | 500 | 0.061 |
| I414 | 200 | total | 500 | 0.008 |
| I414 | 5 | total | 500 | 0.092 |
| I414 | 10 | total | 500 | 0.086 |
| I414 | 25 | total | 500 | 0.080 |
| I414 | 40 | total | 500 | 0.104 |
| I414 | 75 | total | 500 | 0.131 |
| I415 | 5 | m | 500 | 0.007 |
| I415 | 5 | n | 500 | 0.022 |
| I415 | 5 | p | 500 | 0.107 |
| I415 | 25 | m | 800 | 0.002 |
| I415 | 25 | n | 800 | 0.032 |
| I415 | 25 | p | 800 | 0.111 |
| I415 | 50 | m | 800 | 0.002 |
| I415 | 50 | n | 800 | 0.020 |
| I415 | 50 | p | 800 | 0.180 |
| I415 | 75 | m | 800 | 0.003 |
| I415 | 75 | n | 800 | 0.058 |
| I415 | 75 | p | 800 | 0.375 |
| I415 | 100 | m | 800 | 0.031 |
| I415 | 100 | n | 800 | 0.149 |
| I415 | 100 | p | 800 | 0.366 |
| I415 | 150 | m | 800 | 0.003 |
| I415 | 150 | n | 800 | 0.021 |
| I415 | 150 | p | 800 | 0.062 |
| I415 | 200 | m | 800 | 0.001 |
| I415 | 200 | n | 800 | 0.007 |
| I415 | 200 | p | 800 | 0.002 |
| I415 | 0 | total | 500 | 0.213 |

The size-fractionated chlorophyll *a* (chla) data in the surveyed area. Note: m: micro-sized fractionated chla, n: nano-sized fractionated chla, p: pico-sized fractionated chla, total: total chla.

**Supplementary Table S7**

Taxonomy criteria. See website access (http://www.mikrotax.org/Nannotax3/index.php?dir=Coccolithophores/Isochrysidales/Noelaerhabdaceae)

| *Emiliania huxleyi* | LITHS: placolith, skeletal, elliptical, RIM: slits, CA: grill, vacant, CSPH: spherical, monomorphic, V-UNITS: none, R-UNITS: all, c-str, distal shield, prox shield, tube, DETAILS: ca_conjunct, isogyres kinked, isogyres oblique, rim-unicyclic,  Lith size: 2->5µm; Coccosphere size: 4->10µm; Liths per sphere: 10->50 |
| --- | --- |
| *Gephyrocapsa oceanica* | LITHS: placolith, elliptical, CA: bridge, bridge-diagonal, grill,  CSPH: spherical, monomorphic, V-UNITS: none, R-UNITS: all, c-str, distal shield, prox shield, tube, DETAILS: ca_conjunct, isogyres kinked, isogyres oblique, rim-unicyclic,  Lith size: 3.5->6µm; Coccosphere size: 6->10µm; Liths per sphere: 9->35 |
| *Helicosphaera carteri* | LITHS: placolith, asymmetric, RIM: spine(s), wing, CA: bar-transverse,  CSPH: ellipsoidal, varimorphic, V-UNITS: distal shield, R-UNITS: blanket, c-str, px-plate, DETAILS: ca_conjunct, rim-bicyclic, isogyres kinked, isogyres oblique, isogyres separated,  Lith size: 7->12µm; Coccosphere size: 15->25µm; Liths per sphere: 16->40 |
| *Helicosphaera hyalina* | LITHS: helicolith,  No pores, central-area filled with large sized needle-shaped elements  Lith size: 5.5->7.5µm; Coccosphere size: 10->16µm; Liths per sphere: 12->22 (Cros& Fortuño, 2002) |
| *Calcidiscus leptoporus* | LITHS: placolith, circular, sub-circular, RIM: rim-broad, CA: Placoliths: closed,  CSPH: spherical, monomorphic, V-UNITS: distal shield, tube, R-UNITS: prox shield, DETAILS: isogyres oblique, rim-unicyclic,  Lith size: 3->10µm; Coccosphere size: 5->20µm; Liths per sphere: 20->50 |
| *Umbilicosphaera sibogae* | LITHS: placolith, circular, RIM: psh-wider-than-dsh, CA: vacant,  CSPH: spherical, monomorphic, V-UNITS: distal shield, tube, R-UNITS: prox shield, DETAILS: rim-bicyclic, isogyres oblique,  Lith size: 3->6µm; Coccosphere size: 20->30µm; Liths per sphere: 40->200 |
| *Algirosphaera robusta* | LITHS: planolith, elliptical, CA: grill, PROCESS: bulbous, hollow, low, spine, x-section-flat,  CSPH: spherical, varimorphic, V-UNITS: spine, DETAILS: ca_disjunct,  Lith size: 1.5->4.5µm; Coccosphere size: 7->12µm; Liths per sphere: 69->90 |
| *Syracosphaera pulchra* | LITHS: murolith, elliptical, RIM: flange-distal, flange-midwall, outer wall non-imbricate, flange-proximal, ridges, rim-narrow, CA: grill,  CSPH: obpyriform, ovoid, CFC, CFC+process, XC, XC-domal, polymorphic, V-UNITS: lower-rim, R-UNITS: rim, T-UNITS: c-str, DETAILS: ca_disjunct, rim-bicyclic,  Lith size: 4->8µm; Coccosphere size: 15->25µm; Liths per sphere: 20->60 |
| *Umbellosphaera tenuis* | LITHS: placolith, elliptical, irregular, oblong, RIM: ridges, CA: plate,  CSPH: spherical, BC-dimorphic, varimorphic, V-UNITS: prox shield, R-UNITS: distal shield, tube, DETAILS: ca_conjunct, rim-unicyclic,  Lith size: 2->9µm; Coccosphere size: 8->12µm; Liths per sphere: 15->25 |
| *Umbellosphaera irregularis* | LITHS: placolith, elliptical, irregular, oblong, RIM: ridges, CA: plate,  CSPH: spherical, BC-dimorphic, varimorphic, V-UNITS: prox shield, R-UNITS: distal shield, tube, DETAILS: ca_conjunct, rim-unicyclic,  Lith size: 1.4->10µm; Coccosphere size: 10->15µm; Liths per sphere: 14->30 |
| *Oolithotus fragilis* | LITHS: placolith, asymmetric, sub-circular, CA: Placoliths: closed,  CSPH: bowl-shaped, spherical, monomorphic, V-UNITS: distal shield, tube, R-UNITS: prox shield, DETAILS: isogyres oblique, rim-unicyclic,  Lith size: 5->9µm; Coccosphere size: 15->20µm; Liths per sphere: 30->60 |
| *Umbilicosphaera hulburtiana* | LITHS: placolith, elliptical, RIM: psh-bicyclic, CA: vacant,  CSPH: spherical, monomorphic, V-UNITS: distal shield, tube, R-UNITS: prox shield, DETAILS: rim-bicyclic, isogyres oblique,  Lith size: 4->6µm; Coccosphere size: 8->10µm; Liths per sphere: 14->30 |
| *Michaelsarsia elegans* | LITHS: murolith, elliptical, RIM: outer wall non-imbricate, flange-proximal, rim-narrow, CA: grill, axial ridge or plate,  CSPH: ellipsoidal, AAC, Osteolith, CFC, CFC+process, CFC-planolith, polymorphic, R-UNITS: rim, DETAILS: ca_disjunct,  Lith size: 1.8->2.5µm; Coccosphere size: 10->16µm; Liths per sphere: 45->90 |
| *Michaelsarsia adriaticus* | LITHS: murolith, elliptical, RIM: outer wall non-imbricate, flange-proximal, rim-narrow, CA: grill, axial ridge or plate,  CSPH: ellipsoidal, AAC, Osteolith, CFC, CFC+process, CFC-planolith, polymorphic, R-UNITS: rim, DETAILS: ca_disjunct,  Lith size: 1.8->2.5µm; Coccosphere size: 10->20µm; Liths per sphere: 80->150 |
| *Coronosphaera mediterranea* | LITHS: murolith, elliptical, RIM: outer wall acw-imbricate, rim-narrow, CA: grill, axial ridge or plate, PROCESS: boss,  CSPH: spherical, CFC, CFC+process, polymorphic, V-UNITS: c-str, R-UNITS: rim, DETAILS: ca_disjunct, rim-unicyclic,  Lith size: 3->4.5µm; Coccosphere size: 13->16µm; Liths per sphere: 35->80 |
| *Coronosphaera* *binodata* | LITHS: caneolith  Lith size: 2.5->3.5µm; Coccosphere size: 14.6->15.6µm; Liths per sphere: 40->75 (Cros& Fortuño, 2002) |
| *Florisphaera profunda* | LITHS: nannolith-other, lath-shaped, RIM: ridges,  CSPH: bowl-shaped, monomorphic, V-UNITS: none, R-UNITS: all, DETAILS: 1ou, length-fast,  Lith size: 1.5->6µm; Coccosphere size: 6->16µm; |
| *Calciosolenia brasiliensis* | LITHS: murolith, quadrate, RIM: outer wall non-imbricate, rim-narrow, CA: grill,  CSPH: fusiform, varimorphic, V-UNITS: outer-rim, R-UNITS: inner-rim, T-UNITS: c-str, DETAILS: ca_disjunct, rim-bicyclic,  Lith size: 5->7µm; Coccosphere size: 45->95µm; Liths per sphere: 80->190 |
| *Discosphaera tubifera* | LITHS: planolith, elliptical, CA: grill, PROCESS: flaring, open distally, spine,  CSPH: spherical, monomorphic, V-UNITS: calyx, R-UNITS: column, DETAILS: ca_disjunct,  Lith size: 3->8µm; Coccosphere size: 12->20µm; Liths per sphere: 35->70 |
| *Ceratolithus cristatus* | LITHS: nannolith-other, horseshoe-shaped, RIM: spine(s),  CSPH: spherical, V-UNITS: none, R-UNITS: all, DETAILS: 1ou, length-slow,  Lith size: 0->0µm; Segments: 1->1; |
| *Gladiolithus flabellatus* | LITHS: murolith, nannolith-other, tubular, elliptical, RIM: outer wall non-imbricate, CA: plate,  CSPH: bowl-shaped, AAC, AAC-planolith, polymorphic, V-UNITS: all, rim, R-UNITS: none, DETAILS: ca_disjunct, rim-unicyclic,  Lith size: 5->10µm; Coccosphere size: 8->15µm; Liths per sphere: 20->120 |
| *Reticulofenestra sessilis* | LITHS: placolith, elliptical, RIM: inner-tube-broad, CA: Placoliths: closed,  CSPH: spherical, monomorphic, V-UNITS: none, R-UNITS: all, c-str, distal shield, prox shield, tube, DETAILS: isogyres kinked, isogyres oblique, rim-unicyclic,  Lith size: 2.5->4µm; Coccosphere size: 6->10µm; Liths per sphere: 10->30 |
| *Rhabdosphaera clavigera* | LITHS: planolith, elliptical, sub-circular, PROCESS: length-slow, parallel-sided, spine,  CSPH: spherical, BC+process, BC-dimorphic, polymorphic, R-UNITS: spine, DETAILS: ca_disjunct,  Lith size: 3->10µm; Coccosphere size: 20->35µm; Liths per sphere: 30->40 |
| *Coccolithus braarudii* | The holococcolithophore phases of *Coccolithus pelagicus* |
| *Scyphosphaera apsteinii* | LITHS: murolith, tubular, elliptical, RIM: outer wall acw-imbricate, rim-low, CA: plate, pores,  CSPH: spherical, EqC, EqC-tubular, polymorphic, V-UNITS: outer-rim, R-UNITS: c-str, inner-rim, px-plate, DETAILS: rim-bicyclic, isogyres oblique, isogyres separated,  Lith size: 10->25µm; Coccosphere size: 25->45µm; Liths per sphere: 30->40 |
| *Coronosphaera mediterranea* | LITHS: murolith, elliptical, RIM: outer wall acw-imbricate, rim-narrow, CA: grill, axial ridge or plate, PROCESS: boss,  CSPH: spherical, CFC, CFC+process, polymorphic, V-UNITS: c-str, R-UNITS: rim, DETAILS: ca_disjunct, rim-unicyclic,  Lith size: 3->4.5µm; Coccosphere size: 13->16µm; Liths per sphere: 35->80 |

**Supplementary Table S8**

Coccolith enumeration of each station during the investigation.

| Station | Depth(m) | Coccoliths(inds/L) |
| --- | --- | --- |
| I104A | 75 | 65654 |
| I104A | 100 | 59693 |
| I104A | 150 | 18352 |
| I104A | 200 | 124832 |
| I106A | 0 | 134915 |
| I106A | 25 | 10890 |
| I106A | 50 | 21827 |
| I106A | 75 | 13374 |
| I106A | 150 | 91614 |
| I106A | 200 | 58080 |
| I202 | 0 | 35852 |
| I202 | 25 | 23976 |
| I202 | 50 | 27785 |
| I202 | 75 | 17407 |
| I202 | 150 | 72600 |
| I202 | 200 | 86598 |
| I105A | 5 | 121896 |
| I105A | 12 | 161709 |
| I105A | 25 | 71592 |
| I105A | 41 | 51160 |
| I105A | 75 | 24603 |
| I204 | 5 | 27427 |
| I204 | 25 | 3832 |
| I204 | 50 | 44367 |
| I204 | 75 | 19698 |
| I204 | 150 | 42753 |
| I204 | 200 | 85337 |
| I206 | 5 | 3809 |
| I206 | 25 | 15730 |
| I206 | 75 | 7849 |
| I206 | 100 | 51372 |
| I206 | 150 | 61349 |
| I206 | 200 | 41395 |
| I207 | 5 | 19530 |
| I207 | 11 | 5042 |
| I207 | 25 | 22714 |
| I207 | 35 | 30048 |
| I207 | 75 | 20570 |
| I208 | 5 | 36076 |
| I208 | 25 | 5954 |
| I208 | 50 | 5848 |
| I208 | 100 | 12907 |
| I208 | 150 | 13374 |
| I210 | 5 | 605 |
| I210 | 25 | 28395 |
| I210 | 75 | 10285 |
| I210 | 100 | 8470 |
| I210 | 150 | 11251 |
| I210 | 200 | 65138 |
| I212 | 5 | 4246 |
| I212 | 25 | 28233 |
| I212 | 50 | 8067 |
| I212 | 75 | 7592 |
| I212 | 100 | 8470 |
| I212 | 150 | 27023 |
| I212 | 200 | 50215 |
| I214 | 0 | 202 |
| I214 | 25 | 28681 |
| I214 | 50 | 14318 |
| I214 | 75 | 13915 |
| I214 | 100 | 7683 |
| I214 | 150 | 31258 |
| I214 | 200 | 38922 |
| I712 | 0 | 22926 |
| I712 | 25 | 1569 |
| I712 | 50 | 21377 |
| I712 | 75 | 6050 |
| I712 | 100 | 22385 |
| I712 | 150 | 72216 |
| I714 | 0 | 2218 |
| I714 | 25 | 3227 |
| I714 | 75 | 25410 |
| I714 | 100 | 29847 |
| I714 | 150 | 22587 |
| I714 | 200 | 37913 |
| I701 | 0 | 13310 |
| I701 | 10 | 2218 |
| I701 | 25 | 7490 |
| I701 | 40 | 16940 |
| I701 | 75 | 7260 |
| I701 | 150 | 576 |
| I701 | 100 | 3832 |
| I705 | 5 | 5732 |
| I705 | 25 | 7394 |
| I705 | 50 | 2123 |
| I705 | 75 | 16581 |
| I705 | 150 | 11876 |
| I705 | 200 | 77171 |
| I709 | 5 | 1936 |
| I709 | 25 | 3832 |
| I709 | 50 | 1412 |
| I709 | 75 | 5519 |
| I709 | 100 | 6252 |
| I709 | 150 | 2823 |
| I709 | 200 | 64698 |
| I321 | 0 | 2547 |
| I321 | 10 | 6793 |
| I321 | 25 | 18743 |
| I321 | 40 | 3821 |
| I321 | 75 | 8279 |
| I320 | 0 | 3457 |
| I320 | 25 | 3630 |
| I320 | 50 | 54450 |
| I320 | 75 | 4840 |
| I320 | 100 | 13220 |
| I320 | 150 | 12812 |
| I320 | 200 | 9187 |
| I316 | 0 | 45173 |
| I316 | 25 | 23563 |
| I316 | 50 | 54569 |
| I316 | 75 | 7170 |
| I316 | 100 | 5378 |
| I316 | 150 | 14117 |
| I316 | 200 | 32267 |
| I314 | 5 | 15422 |
| I314 | 10 | 3832 |
| I314 | 25 | 5457 |
| I314 | 40 | 712 |
| I314 | 75 | 2420 |
| I401 | 0 | 2622 |
| I401 | 25 | 7260 |
| I401 | 50 | 13893 |
| I401 | 75 | 1643 |
| I401 | 100 | 4033 |
| I401 | 150 | 192 |
| I401 | 200 | 97069 |
| I307 | 0 | 5220 |
| I307 | 25 | 10840 |
| I307 | 50 | 9965 |
| I307 | 75 | 19410 |
| I307 | 100 | 11524 |
| I307 | 150 | 9490 |
| I307 | 200 | 45790 |
| I303 | 5 | 103593 |
| I303 | 25 | 27561 |
| I303 | 50 | 27172 |
| I303 | 75 | 7118 |
| I303 | 100 | 10202 |
| I303 | 150 | 32267 |
| I303 | 200 | 84476 |
| I305 | 5 | 20167 |
| I305 | 10 | 21590 |
| I305 | 40 | 23251 |
| I305 | 75 | 16346 |
| I403 | 25 | 3322 |
| I403 | 50 | 27759 |
| I403 | 75 | 9965 |
| I403 | 100 | 9016 |
| I403 | 150 | 17619 |
| I403 | 200 | 78045 |
| I405 | 0 | 21287 |
| I405 | 25 | 8491 |
| I405 | 50 | 2241 |
| I405 | 75 | 54198 |
| I405 | 100 | 29474 |
| I405 | 150 | 34149 |
| I405 | 200 | 60025 |
| I407 | 0 | 21679 |
| I407 | 25 | 6302 |
| I407 | 50 | 8541 |
| I407 | 75 | 10756 |
| I407 | 100 | 23014 |
| I407 | 150 | 13286 |
| I407 | 200 | 83893 |
| I408 | 0 | 3781 |
| I408 | 10 | 4271 |
| I408 | 25 | 3361 |
| I408 | 40 | 7118 |
| I408 | 75 | 7260 |
| I414 | 5 | 1008 |
| I414 | 10 | 4302 |
| I414 | 25 | 538 |
| I414 | 40 | 3277 |
| I414 | 75 | 5546 |
| I409 | 0 | 504 |
| I409 | 25 | 1898 |
| I409 | 50 | 1274 |
| I409 | 75 | 12352 |
| I409 | 100 | 9831 |
| I409 | 150 | 11151 |
| I409 | 200 | 62265 |
| I411 | 0 | 1613 |
| I411 | 25 | 448 |
| I411 | 50 | 896 |
| I411 | 75 | 2689 |
| I413 | 0 | 15909 |
| I411 | 100 | 13512 |
| I411 | 150 | 202 |
| I411 | 200 | 13524 |
| I413 | 25 | 4745 |
| I413 | 50 | 5457 |
| I413 | 75 | 3277 |
| I413 | 100 | 3084 |
| I413 | 150 | 1661 |
| I415 | 5 | 224 |
| I415 | 25 | 807 |
| I415 | 50 | 4033 |
| I415 | 75 | 7843 |
| I415 | 100 | 5378 |
| I415 | 200 | 27981 |
